# Utilization of Psychiatric Hospital Services Following Intensive Home Treatment: A Nonrandomized Clinical Trial

**DOI:** 10.1001/jamanetworkopen.2024.45042

**Published:** 2024-11-15

**Authors:** Andreas Bechdolf, Konstantinos Nikolaidis, Sebastian von Peter, Gerhard Längle, Peter Brieger, Jürgen Timm, Reinhold Killian, Lasse Fischer, Svenja Raschmann, Julian Schwarz, Martin Holzke, Sandeep Rout, Constance Hirschmeier, Johannes Hamann, Uwe Herwig, Janina Richter, Johanna Baumgardt, Stefan Weinmann

**Affiliations:** 1 Department of Psychiatry, Psychotherapy, and Psychosomatics incorporating FRITZ am Urban and soulspace, Vivantes Hospital am Urban and Vivantes Hospital im Friedrichshain, Berlin, Germany; 2 Department of Psychiatry and Psychotherapy, CCM, Charité - Universitätsmedizin Berlin, corporate member of Freie Universität Berlin and Humboldt-Universität zu Berlin, Germany; 3 German Center for Mental Health (DZPG), Berlin-Potsdam site, Germany; 4 Faculty of Health Sciences Brandenburg, Brandenburg Medical University Theodor Fontane, Neuruppin, Germany; 5 Department of Psychiatry and Psychotherapy, Center for Mental Health, Immanuel Hospital Rüdersdorf, Brandenburg Medical School Theodor Fontane, Rüdersdorf, Germany; 6 Center for Psychiatry South Württemberg, Department of Psychiatry and Psychotherapy Zwiefalten, Zwiefalten, Germany; 7 Clinic for Psychiatry and Psychosomatics of Reutlingen (PP.rt), Academic Teaching Hospital of the University of Tübingen, Reutlingen, Germany; 8 General Psychiatry and Psychotherapy Division, Department of Psychiatry and Psychotherapy, University Hospital Tübingen and Medical Faculty of the University of Tübingen, Tübingen, Germany; 9 kbo-Isar-Amper Hospital Munich Region, Academic Teaching Hospital of Ludwig-Maximilians-University Munich, Haar near Munich, Germany; 10 Competence Center for Clinical Studies Bremen, Biometrics Department, University of Bremen, Bremen, Germany; 11 Section of Health Economics and Health Services Research, Department of Psychiatry and Psychotherapy II of Ulm University at Bezirkskrankenhaus Günzburg, Günzburg, Germany; 12 Center for Psychiatry South Württemberg, Department of Psychiatry and Psychotherapy I, University of Ulm, Weissenau, Germany; 13 Department of Psychiatry, Psychotherapy, and Psychosomatics, Vivantes Neukölln Hospital, Berlin, Germany; 14 Mainkofen District Hospital, Deggendorf, Germany; 15 Reichenau Center for Psychiatry, Academic Teaching Hospital University of Konstanz, Reichenau, Germany; 16 Department of Psychiatry and Psychotherapy III, University of Ulm, Ulm, Germany; 17 Psychiatric University Hospital Zurich, Zurich, Switzerland; 18 Department of Psychiatry and Psychotherapy, University Hospital Tübingen, Tübingen, Germany; 19 Scientific Institute of the AOK (WIdO), Berlin, Germany; 20 University Psychiatric Clinics (UPK) Basel, Faculty of Medicine University of Basel, Basel, Switzerland; 21 Center for Integrative Psychiatry, University Hospital Schleswig-Holstein, Germany

## Abstract

**Importance:**

Home treatment (HT) has been associated with fewer inpatient treatment (IT) readmission days but lacks evidence on reducing combined psychiatric hospital service use (IT, HT, day clinic).

**Objective:**

To assess the association of intensive home treatment (IHT) compared with IT regarding readmission rate, social outcomes, and clinical outcomes.

**Design, Setting, and Participants:**

This quasi-experimental, nonrandomized trial was conducted from 2020 to 2022 in 10 psychiatric hospitals in Germany. Propensity score (PS) matching was used to compare both treatment models at the 12-month follow-up using standardized instruments and routine hospital data. All patients were screened until the target sample size was reached, based on these criteria: stable residence with privacy for sessions, no child welfare risk, primary diagnosis within *International Statistical Classification of Diseases and Related Health Problems, Tenth Revision* codes F0X to F6X, residence in the catchment area, no commitment order, no acute suicidality or severe aggression requiring hospitalization, capacity to consent, not participating in other interventional studies, sufficient German language skills, no substantial cognitive deficits or intellectual impairment, and no more than 7 days in IHT or IT before recruitment. Statistical analysis was performed from February to November 2023.

**Intervention:**

IHT provided daily acute psychiatric treatment at home, while IT was psychiatric inpatient treatment as usual. The mean treatment duration of the index treatment was 37.2 days for IHT and 28.2 days for IT.

**Main Outcomes and Measures:**

The inpatient readmission rate was the primary outcome. Secondary outcomes were combined readmission rate, total inpatient days, job integration, quality of life, psychosocial functioning, symptom severity, and recovery.

**Results:**

Of 1396 individuals, 200 patients receiving IHT and 200 patients receiving IT were included (264 female [65%]; mean [SD] age, 45.45 [15.83] years [range, 18-88 years]). Baseline sociodemographic and psychometric characteristics did not differ significantly between the groups. At 12-month follow-up, patients in the IHT group had lower inpatient readmission rate (IHT vs IT: 31.12% vs 49.74% IT; mean difference, 18% [95% CI, 9%-28%; *P* < .001), combined readmission rate (mean difference, 13% [95% CI, 4%-24%; *P* < .001), and fewer inpatient days (mean difference, 6.82 days; *P* < .001) than the IT group.

**Conclusions and Relevance:**

This nonrandomized clinical trial found that patients receiving IHT had a lower likelihood of utilizing hospital-based psychiatric services and spent fewer inpatient days, suggesting that IHT is a viable alternative to IT.

**Trial Registration:**

ClinicalTrials.gov Identifier: NCT04745507

## Key Points

**Question:**

What are the outcomes of intensive home treatment (IHT) compared with inpatient treatment (IT) in terms of utilization of psychiatric hospital services?

**Findings:**

In this 12-month follow-up nonrandomized trial involving 400 participants from 10 psychiatric hospitals in Germany, IHT was associated with lower inpatient readmission rate (−18%), lower rate of readmission to either inpatient, day clinic, or IHT (−13%) as well as fewer inpatient treatment days (−6.82 days) compared with IT. There were no significant group differences in clinical and social outcomes at the 12-month follow-up.

**Meaning:**

These results suggest that IHT is a viable alternative to IT for individuals with psychiatric crises otherwise requiring hospital admission.

## Teaser

This nonrandomized clinical trial investigates the association of intensive home treatment vs inpatient treatment with readmission rate, social outcomes, and clinical outcomes.

## Introduction

Home treatment (HT) models, such as crisis resolution teams or intensive home treatment (IHT),^1^ are implemented in a variety of mental health systems.^2^ HT has emerged as an evidence-based alternative to hospital admission in individuals with acute mental illness.^3^ However, all studies included in the most recent Cochrane Review^3^ were older than 20 years,^4,5,6,7,8,9,10^ examined small sample sizes,^6,9,10,11^ and included patients who were not solely treated at home but also in acute residential facilities.^10^

More recent randomized clinical trials (RCTs) of HT models^12,13^ demonstrated a reduction of inpatient days without worsening of symptoms or other clinical or social parameters but were unable to substantiate earlier findings of a reduction in inpatient readmission rate.^4^ Moreover, none of those trials addressed the combined utilization of psychiatric hospital services (IT, HT, day clinic) as an outcome, although contemporary psychiatric care offers more flexible support than just IT.^14^ Therefore, large-scale, multicenter trials evaluating HT models are needed to evaluate HT as part of a contemporary mental health care system and explore the utilization of all psychiatric hospital services as an outcome, rather than only IT.

After the limited availability of HT services in Germany,^15,16^ a specific form of IHT, known as inpatient equivalent home treatment (IEHT), was introduced in 2017 and was made available to German psychiatric hospitals through reimbursement by public health insurance.^17^ It shares specific characteristics with various HT models, however, unlike other HT models, it is highly regulated. IEHT teams need to provide daily visits and meet predefined team and procedure standards, which offers an opportunity to conduct a high-quality multicenter evaluation due to its standardization across sites.

Recently, a retrospective pilot study on the German IHT model with matched pairs^18^ showed fewer inpatient readmissions and inpatient days at 12-month-follow-up in the IHT group compared with patients with initial inpatient treatment. However, the study was conducted only in 1 treatment center, and matching was limited due to the retrospective nature of the trial. To evaluate IHT comprehensively, we conducted a prospective large-scale, multicenter, quasi-experimental trial, called the AKtiV-trial (German: Aufsuchende Krisenbehandlung mit teambasierter und integrierter Versorgung: Evaluation der stationsäquivalenten psychiatrischen Behandlung [StäB nach §115d SGB V]; English: “Outreach Crisis Intervention With a Team-Based and Integrative Model of Treatment: Evaluation of the Inpatient Equivalent Home Treatment [IEHT according to the German Social Code Book V §115d]”).^19^ The first findings indicated that the use of IHT was associated with more treatment satisfaction and higher involvement in clinical decision-making compared with those receiving IT.^20^

This study presents the results of the AKtiV trial comparing IHT with IT, focusing on the inpatient readmission rate and the combined readmission rate (IT plus day clinic plus IHT) within 12 months after admission to the index treatment. Additional outcomes, including total inpatient days, job integration, quality of life, psychosocial functioning, symptom severity, and recovery orientation within 12 months following admission are also examined.

## Methods

### Study Design

We report a 12-month follow-up quasi-experimental, nonrandomized clinical trial with 2 cohorts conducted between January 2021 and December 2022 in 10 psychiatric hospitals throughout various regions in Germany where IHT had been implemented (eTable in Supplement 1). The study was approved by the ethics committee of the Brandenburg Medical School Theodor Fontane and by all ethics committees of the participating study sites (see Supplement 2 for the study protocol). Written informed consent was obtained from participants or their legal guardians, accompanied by trial information. The Transparent Reporting of Evaluations With Nonrandomized Designs (TREND) reporting guideline was followed.

### Study Population

The study included patients who received either IHT or IT. Participants were admitted to IHT or IT due to an acute mental health crisis without acute suicidality or extensive aggressive behavior requiring hospitalization. Crises typically included substantial deterioration of positive, negative, or affective symptoms or anxiety with impaired psychosocial functioning and substantial distress. To be eligible for IHT, participants needed a stable residence providing privacy for conducting psychiatric/psychotherapeutic sessions and, if children lived in the household, there should be no risk of endangering child welfare. Additional inclusion criteria were the presence of a primary diagnosis within *International Statistical Classification of Diseases and Related Health Problems, Tenth Revision (ICD-10)* codes F0X, F1X, F2X, F3X, F4X, F5X, or F6X, living in the catchment area, no commitment order, capacity for informed consent, nonparticipation in additional interventional studies, sufficient German language skills for study participation, no substantial cognitive deficits from severe organic brain disease, no diagnosis of intellectual impairment, and patients should not have spent more than 7 days in either IHT or IT before recruitment.

### Interventions

IHT, in the form of IEHT, is a team-based home treatment for psychiatric crises that would otherwise require IT. The framework for IHT is determined, regulated, and monitored by the umbrella organizations of the health insurance.^17^ The IHT team includes at least 1 psychiatrist, 1 nurse, and a third profession (psychologist, social worker, occupational or physiotherapist). The IHT team is responsible 24/7 and ensures daily face-to-face contact, weekly team meetings, and offers comprehensive psychiatric and somatic care, including diagnostics, medications, psychotherapy, and psychosocial interventions. Daily contacts with team members as well as 1 weekly meeting with a psychiatrist are needed to get reimbursement by health insurance. The psychiatrist assesses eligibility and creates a treatment plan. Discharge management resembles IT, following established guidelines for psychiatric care in German hospitals. IT followed German hospital guidelines for psychiatric care.

### Sample Size

The sample size calculation for the primary outcome (readmission rate at 12-month follow-up) used international studies^3,4,10,21^ and routine data analysis from the participating study centers. The calculation was based on their 12-month inpatient readmission rate of 52.1% (N = 37 007). A value for the reduction rate of IHT readmission rate was calculated from a weighted mean of IHT effects in literature^3^ and a pilot study^22^ with IHT reduction ratios between 0.20 and 0.33 resulting in an anticipated IHT rate of 37.4% for a 2-sided χ^2^ test with 5% α error and 80% power, nQuery Advisor 7.0 calculated a minimum required sample size of 360 patients, 180 in each group, to detect the anticipated difference. Factoring in a 10% nonresponder rate, approximately 400 patients would need to be included in the study.

### Recruitment Process

Until the planned sample size was achieved, all patients were screened for inclusion criteria. If inclusion criteria were met and written consent was obtained, patients were enrolled in the study. Each enrolled individual in the IHT study group, after receiving a propensity score (PS) estimating the probability of receiving IHT in the specific study center, was matched with a patient in the IT group based on this PS.

This PS matching process identified the most similar PS, resulting in the formation of pairs with comparable clinical and sociodemographic characteristics. The PS for each potential participant was derived from a logistic regression analysis considering the following variables: total number of days in psychiatric IT or IHT in the study center in the last 2 years, main psychiatric diagnosis, age, and gender. Previous simulations showed that a 0.1 difference was a suitable criterion for prospective matching. IT patients whose PS differed by less than 0.1 from the IHT patient’s PS were considered a match, indicating a similar likelihood of admission to the IEHT. The main diagnosis was the key variable for PS matching, so matches were made within the same diagnostic group.

The recruitment of patients proceeded according to the study protocol^19^ with no major modifications except the following: an alternative PS matching protocol, applied for 48 dyads in total, was included if no matching could be achieved within 5 months, allowing to match with a larger PS difference but within the same diagnostic group. The stipulation of equal recruitment numbers for all centers was lifted.

### Outcomes and Assessments

The primary outcome was the inpatient readmission rate within 12 months. Secondary outcomes included readmission rate to any psychiatric service (IT, IHT, or day clinic), inpatient hospital days, job integration, quality of life, psychosocial functioning, symptom severity, and recovery within 12 months. Hypotheses and rationales for these outcomes were formulated in the study protocol and the statistical analysis plan.

All assessments were conducted in the hospital or at the patient’s homes. Assessments were conducted at baseline (within 1 week after admission and at the end of the index treatment), at 6-month follow-up (6 months after admission), and 12-month follow-up (12 months after admission). Patients received 50 € for study participation after completing the 12-month follow-up assessment.

Readmission rate, combined readmission rate, and inpatient hospital days were assessed using the German version of the client sociodemographic and service receipt inventory^23^ and hospital routine data. Symptom severity was assessed by the total score of the German version of the Health of the Nations Outcome Scale (HoNOS-D),^24^ psychosocial functioning with the German version of the Personal and Social Performance Scale (PSP),^25^ quality of life with the German version of the EQ-5D-5L and the German utility value set^26^ and recovery with the German version of the Recovery Assessment Scale.^27^ Sociodemographic and clinical variables were also collected at baseline.

Dropout was defined as participants withdrawing consent. Raters were not blinded to study condition assignments. Serious adverse events (SAE) were assessed according to the German Drug Law and the Ordinance on GCP,^28^ excluding the case of rehospitalization in a mental health facility.

The study staff, trained in standard operating procedures (SOP) and good scientific practices, were not involved in clinical care. Raters’ interrater reliability for the HoNOS-D and the PSP scale was monitored using clinical case vignettes, showing an intraclass correlation coefficient of 0.773.

### Data Management

Data collection adhered to the study’s data protection concept and the European General Data Protection Regulation.^29^ Data was entered into an electronic case report form and transferred to the Competence Center for Clinical Studies Bremen (KKSB), where plausibility, completeness checks, data preparation, and analysis using SAS version 9.4 were conducted. Questionable entries initiated a query process according to the KKSB SOP.

### Statistical Analysis

Statistical analyses were performed from February to November 2023 using SAS version 9.4 (SAS Institute) and SYSTAT 13.2 (Grafiti LLC), based on the statistical analysis plan (Supplement 3). Analyses were conducted on an intention-to-treat (ITT) basis, evaluating all patients as per their initial treatment assignment. The main results were obtained by full case analyses (FCA) including only patients with observed values for all variables. To support the findings, the same analyses were conducted using a multiple imputation (MI) scheme, replacing missing values with randomized estimates of an appropriate regression model 100 times and combining the results by Rubin rules.^30^ Regarding the primary outcome analyses of a best-case scenario, considering all missing IHT cases as nonreadmissions and all missing IT cases as readmissions, and a worst-case scenario, considering all missing IHT cases as readmissions and all missing IC cases as nonreadmissions, were conducted. Descriptive statistics were calculated for all quantitative data. For categorical variables, numerical and percentage data were separately determined for the 2 treatment groups. For metric variables, the mean, SD, median, minimum, and maximum were calculated. For the primary outcome, the null hypothesis was tested at a significance level of 5% using a *z*-test. Secondary outcomes were tested exploratorily at a significance level of 5%. For binary data, a *z*-test was used. For numerical variables, the assumption of normality was assessed graphically using pilot data. If the data were normally distributed, *t* tests for unequal variances (Welch tests) were used; otherwise, the Mann-Whitney *U* test was used.

## Results

### Sample Characteristics

There were 1396 patients approached between January 1, 2021, and December 31, 2021 (Figure). The ITT sample consisted of 400 patients (264 female [65%]; mean [SD] age, 45.45 [15.83] years [range, 18-88 years]) divided into 2 equal cohorts; 200 IHT patients and 200 statistically matched IT patients. There were no statistically significant differences between both treatment groups regarding all variables used for PS matching (Table 1) as well as all baseline sociodemographic, psychiatric health care utilization, and psychometric characteristics (Table 2).

**Figure.**
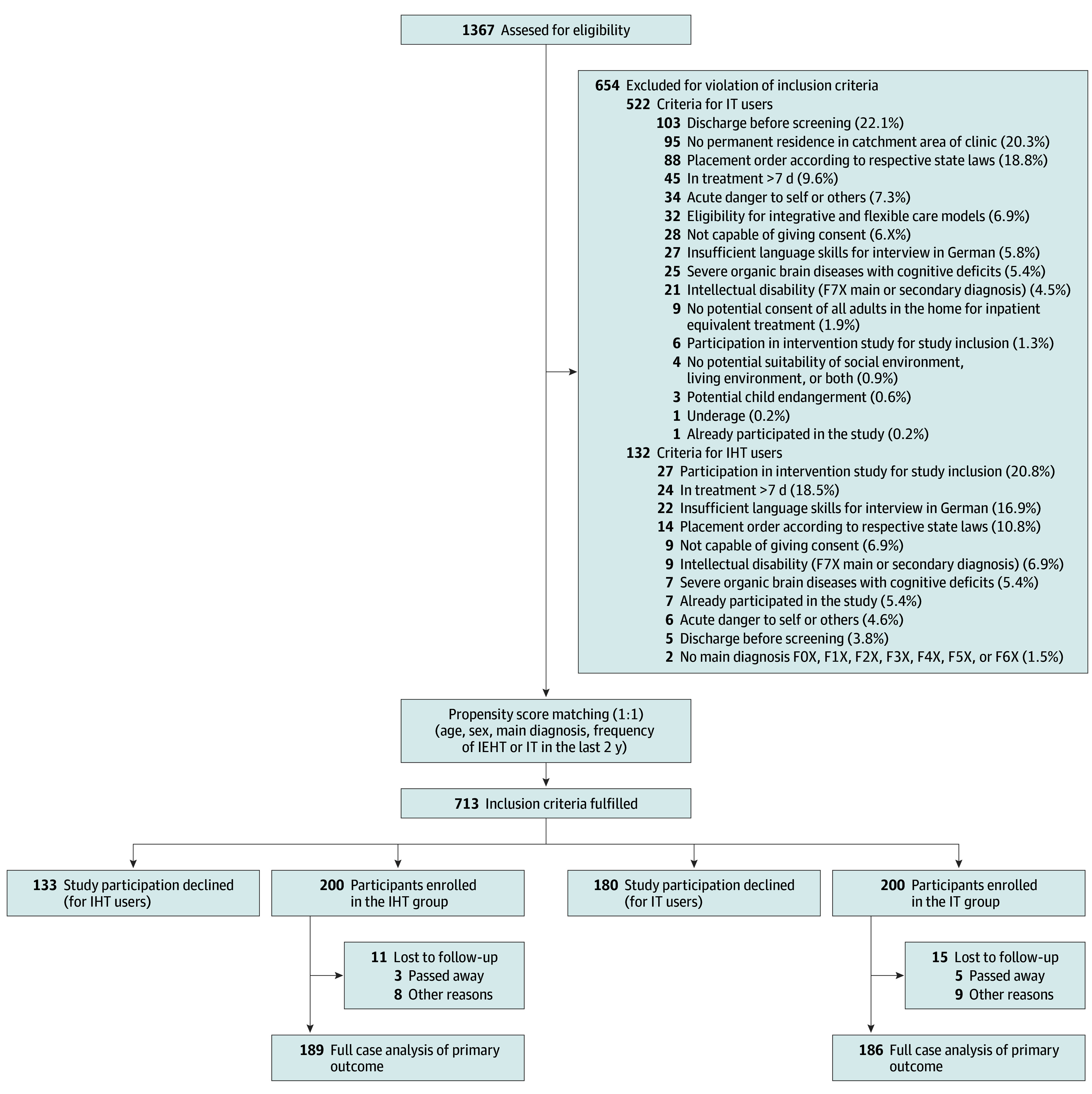
Flowchart of Study Participants IHT indicates intensive home treatment; IT, inpatient treatment.

**Table 1.** Propensity Score Variables of Participants in the IHT Group vs the IT Group at Recruitment

Propensity score variables	Group		*P* value
	IHT (n = 200)	IT (n = 200)	
Sex, No. (%)			
Female	136 (68.0)	128 (64.0)	.40^a^
Male	164 (32.0)	72 (36.0)	
Age, mean (SD), y	45.32 (15.8)	45.49 (15.9)	.72^b^
No. of IHT or IT in the 2 y prior to the start of the index treatment, mean (SD)	1.59 (2.9)	1.21 (2.4)	.05^b^
*ICD-10* code for main diagnosis on admission to index treatment, No. (%)	1:1 Matching		
F0X	1 (0.5)	0	
F1X	13 (6.5)	13 (6.5)	
F2X	43 (21.5)	43 (21.5)	
F3X	94 (47.0)	95 (47.5)	
F4X	31 (15.5)	31 (15.5)	
F5X	2 (1.0)	2 (1.0)	
F6X	16 (8.0)	16 (8.0)	

Abbreviations: *ICD-10, International Statistical Classification of Diseases and Related Health Problems, Tenth Revision*; IHT, intensive home treatment; IT, inpatient treatment.

^a^ Calculated using the χ^2^ test.

^b^ Calculated using the Mann-Whitney *U* test.

**Table 2.** Characteristics of Participants in the IHT Group vs the IT Group at Recruitment

Sociodemographic characteristics	IHT (n = 200)	IT (n = 200)	*P* value
Mother tongue German, No. (%)	167 (83.5)	166 (83.0)	.89^a^
Marital status, No. (%)			
Single	107 (53.5)	129 (64.5)	.11^a^
Married	63 (31.6)	45 (22.5)	
In a relationship	28 (15.0)	26 (13.0)	
No information	2 (1.0)	0 (0.0)	
Highest school-leaving qualification, No. (%)			
No school-leaving qualification	2 (1.0)	7 (3.5)	.13^c^
Elementary school	41 (20.5)	36 (18.0)	
Secondary school leaving certificate	60 (30.0)	79 (39.5)	
University entrance qualification	26 (13.0)	18 (9.0)	
High school diploma	65 (32.5)	57 (28.5)	
Other	6 (3.0)	3 (1.5)	
Highest professional qualification, No. (%)			
No training	43 (21.5)	48 (24.0)	.31^c^
Apprenticeship	82 (41.0)	76 (38.0)	
Vocational school	23 (11.5)	24 (12.0)	
University diploma	43 (21.5)	33 (16.5)	
Doctorate	0 (0.0)	3 (1.5)	
Other	9 (4.5)	15 (7.5)	
Missing data	0 (0.0)	1 (0.5)	
Employment situation			
Employed in the primary labor market	51 (25.5)	65 (32.5)	.55^a^
Employed in the secondary labor market	9 (4.5)	12 (6.0)	
Unemployed	43 (21.5)	39 (19.5)	
Disabled or occupationally disabled	45 (22.5)	34 (17.0)	
On pension	26 (13.0)	22 (11.0)	
Undergoing training/retraining	14 (7.0)	10 (5.0)	
Other	14 (7.0)	16 (8.0)	
Place of residence [urban area], No. (%)	95 (47.5)	95 (47.5)	>.99^a^
Net household income [monthly], mean (SD), €	1764 (1449)	1605 (1238)	.23^b^
Source of income, No. (%)			
Salary/wage	40 (20.0)	56 (28.0)	.39^a^
Pension	43 (21.5)	45 (22.5)	
Social benefits,	46 (23.0)	52 (26.0)	
Family support	23 (11.5)	19 (9.5)	
Other	40 (20.0)	33 (16.5)	
Missing data	2 (1.0)	1 (0.5)	
Utilization of psychiatric services			
At least 1 inpatient treatment before index treatment, No. (%)	156 (78.0)	153 (76.5)	.55^a^
Age at first inpatient treatment, mean (SD), y	34.82 (16.1)	34.94 (16.6)	.91^b^
No. of previous inpatient treatments, mean (SD)	6.70 (18.3)	5.32 (8.7)	.92^b^
Psychometric characteristics			
HoNOS-D score, mean (SD)	14.66 (5.4)	15.34 (5.5)	.17^b^
PSP score, mean (SD)	56.04 (12.3)	56.11 (12.9)	.76^b^
EQ-5D-5L score, mean (SD)	0.62 (0.3)	0.64 (0.3)	.38^b^
RAS-G score, mean (SD)	47.29 (9.7)	45.79 (9.5)	.14^b^

Abbreviations: EQ-5D-5L, German version of the European Quality of Life 5 Dimensions 5 Level Version questionnaire; HoNOS-D, German version of the Health of the Nation Outcome Scale; IHT, intensive home treatment; IT, inpatient treatment; PSP, German version of the Personal and Social Performance questionnaire; RAS-G, German version of the Recovery Assessment Scale.

^a^ Calculated using the χ^2^ test.

^b^ Calculated using the Mann-Whitney U test.

^c^ Calculated using the Fisher exact test.

### Index Treatment

The duration of the index treatments differed significantly between the groups. The mean (SD) duration of the IHT group was 37.2 days (24.9) days vs 28.2 (30.6) days for the IT group (*P* < .001).

### Primary Outcome

In the FCA, IHT was associated with a significantly lower inpatient readmission rate compared with IT (IHT vs IT: 31.12% vs 49.74%; mean difference, 18% [95% CI, 9%-28%]; *P* < .001). As there were only 25 (6.25%) missing values for the primary outcome, which were evenly distributed (IHT n = 14 [7%]; IT n = 11 [5.5%]) between both groups, the risk of bias is low. The distribution test of the PS variables concerning missing values did not show significant differences. Thus, missing values were assumed to be missing completely at random (MCAR), implying that the requirements for conducting an MI are fulfilled. The MI analysis yielded the same *P* value as FCA. Best-case (mean difference, 24% [95% CI, 14%-33%]; *P* < .001) and worst-case (mean difference, 11% [95% CI, 1%-21%]; *P* = .02) scenarios regarding missing values also showed IHT’s significantly lower readmission rate compared with IT (Table 3). Adjusting for key covariates, such as PS and differences between study sites, did not change the statistical significance results between treatment groups.

**Table 3.** Inpatient Readmissions Within 12 Months After Index Treatment

	Group				*P* value
	IHT		IT		
	Total No.	At least 1 inpatient readmission, No. (%)	Total No.	At least 1 inpatient readmission, No. (%)	
Inpatient readmissions					
Full-case analysis	186	58 (31.18)	189	94 (49.74)	<.001^c^
Best-case scenario^a^	200	58 (29.00)	200	105 (52.50)	<.001^c^
Worst-case scenario^b^	200	72 (36.00)	200	94 (47.00)	.03^c^
Combined readmissions					
Full-case analysis	190	86 (45.26)	190	112 (58.95)	.01^c^

Abbreviations: IHT, intensive home treatment; IT, inpatient treatment.

^a^ The best-case scenario assumes that all missing values correspond to patients who were not readmitted as inpatients.

^b^ The worst-case scenario assumes that all missing values correspond to patients who were readmitted as inpatients.

^c^ Calculated with the *z*-test.

### Secondary Outcomes

Concerning the combined readmission rate, data showed that 45.26% of the IHT group and 58.95% of the IT group were admitted to either IT, IHT, or day clinic services within 12 months after admission to the index treatment. This difference is statistically significant (mean difference, 13% [95% CI, 4%-24%]; *P* = .01). With only 5% missing values, the result of the FCA is considered reliable. We found no difference in the distribution of PS variables between missing and available values confirming the MCAR assumption for the imputation analysis. The MI analysis also found a significant group difference in favor of IHT (mean difference, 13% [95% CI, 3%-23%]; *P* = .01).

The mean (SD) inpatient days spent in the study centers during the period between the end of the index treatment and the 12-month follow-up was 14.29 (36.82) days for the IHT group and 21.11 (42.08) days for the IT group (*P* < .001 [Mann-Whitney test]). The inpatient days were confined to study centers, ensuring no missing values. No significant differences were found between groups in job integration, quality of life, psychosocial functioning, symptom severity, or recovery at 12-month follow-up (Table 4).

**Table 4.** Clinical and Social Outcomes 12 Months After Index Treatment

Outcome	Group				Full case *P* value, analysis	Multiple imputation *P* value, analysis
	IHT		IT			
	No.	Mean (SD)	No.	Mean (SD)		
HoNOS-D	179	10.21 (5.93)	171	10.91 (5.76)	.26^b^	.14^c^
PSP	178	67.65 (12.74)	171	66.13 (14.67)	.48^b^	.55^b^
EQ-5D-5L	179	0.68 (0.33)	169	0.72 (0.28)	.45^b^	.48^b^
RAS-G	179	48.42 (11.21)	168	50.45 (10.80)	.09^b^	.05^c^
Job integration	181	0.29 (0.45)	177	0.34 (0.48)	.24^a^	.29^a^

Abbreviations: EQ-5D-5L, German version of the European Quality of Life 5 Dimensions 5 Level Version questionnaire; HoNOS-D, German version of the Health of the Nation Outcome Scale; IHT, intensive home treatment; IT, inpatient treatment; PSP, German version of the Personal and Social Performance questionnaire; RAS-G, German version of the Recovery Assessment Scale.

^a^ Calculated using the *z*-test.

^b^ Calculated using the Mann-Whitney *U* test.

^c^ Calculated using the Welch test.

### Adverse Events

In each group, 6 SAEs were reported. No SAEs in the IHT group were related to the study. In the IT group, 1 SAE’s connection to the study was unclear. Eight deaths occurred (3 in IHT, 5 in IT), all unrelated to the study. The SAE analysis found no substantial safety issues related to the intervention.

## Discussion

Our study found that IHT was associated with fewer inpatient readmissions, fewer combined readmissions, and fewer inpatient days spent within 1 year compared with IT. No significant differences were observed between the groups at 12-month follow-up regarding symptom severity, job integration, quality of life, psychosocial functioning, and recovery.

Our trial overcomes limitations of earlier studies by evaluating intensive HT in a large-scale, multicenter trial, with rather standardized HT across sites, in patients at home, in the context of a contemporary mental health care system, and by assessing combined utilization of psychiatric hospital service in addition to IT as an outcome measure. We found significantly lower inpatient and combined readmission rates with IHT vs IT, a novel outcome not previously assessed to our knowledge. Lower inpatient and combined readmission rates for HT were also reported by Johnson and colleagues,^4^ although focusing on hospital and crisis house readmission rates, whereas our study defined combined readmission as readmission to IT, IHT, or day clinics. This suggests that IHT may contribute to a substantial change in the utilization of psychiatric services among users.^18^ Recent RCTs did not find a lower admission rate following HT,^12,13^ likely due to higher treatment intensity and duration in our IHT compared with the HT models in those trials. Nevertheless, these trials showed a 30% to 36% reduction in hospital bed days after IHT compared with treatment as usual, aligning with our reported 32% reduction in inpatient days within 12 months. No differences were observed in quality of life, symptom severity, or SAE between both cohorts at follow-up, consistent with findings from other RCTs on HT.^4,12,13^

This evidence shows that shifting the location of service delivery to people’s homes also in severe crises is possible and has several advantages. IHT seems able to build on resources in the community as symptoms can be contextualized and patients and relatives can immediately apply what they have experienced with the multidisciplinary team. Coping with a severe crisis without hospitalization might be 1 factor preventing inpatient admissions in the future as well as individual access to the team, communication at eye level, and networking with other service provider organizations.^31^ Relatives are more likely to support home-based treatment when the HT is reliable daily, accessible for relatives, and offers practical help in social issues besides only medical care. In this case, the family does need to shoulder the costs of relocating treatment from the hospital to the community.^32^

### Limitations

Our trial had limitations. Randomization was not possible due to IHT being part of the routine services, and the study’s 12-month follow-up limited the assessment of the long-term effects of IHT. As with other trials, our findings are limited by the study’s exclusion criteria, such as applicability for individuals requiring immediate hospitalization due to suicidality, imminent self-harm, or danger to others. Additionally, the findings may be affected by changes in mental health service delivery due to the COVID-19 pandemic.^33,34,35^

## Conclusions

In this nonrandomized clinical trial, IHT was found to have comparable outcomes with IT without compromising safety, with fewer inpatient days, and less frequent use of IT, IHT, or day clinic services. These results suggest that IHT is a viable alternative to IT. Future studies should explore the key elements of IHT and the patients who benefit most.

## Notes

Supplement 1. **eTable.** Psychiatric Hospital Participating in the Trial and Their IHT-Unit Capacities at Recruitment

Supplement 2.
Trial Protocol

Supplement 3.
Statistical Analysis Plan

Supplement 4.
Data Sharing Statement
