# Supplementary material for: Utilization of Psychiatric Hospital Services Following Intensive Home Treatment: A Nonrandomized Clinical Trial
Source: JAMA Netw Open. 2024 Nov 15;7(11):e2445042. doi: 10.1001/jamanetworkopen.2024.45042 (PMC11568461; doi:10.1001/jamanetworkopen.2024.45042)
Supplement: Supplement 1. — eTable. Psychiatric Hospital Participating in the Trial and Their IHT-Unit Capacities at Recruitment [file jamanetwopen-e2445042-s001.pdf]

Supplemental Online Content

Bechdolf A, Nikolaidis K, von Peter S, et al. Utilization of psychiatric hospital services following intensive home treatment: a nonrandomized clinical trial. *JAMA Netw Open.* 2024;7(11):e2445042. doi:10.1001/jamanetworkopen.2024.45042

eTable. Psychiatric Hospital Participating in the Trial and their IHT-Unit Capacities at Recruitment

This supplemental material has been provided by the authors to give readers additional information about their work.

© 2024 Bechdolf A et al. *JAMA Network Open*.

eTable. Psychiatric hospital participating in the trial and their IHT-unit capacities at recruitment

Name of the study hospital	Region	IHT-unit capacity	IHT-unit Implementation	Catchment area
Immanuel Clinic Rüdersdorf	Brandenburg (Northeastern Germany)	8	2019	Urban/rural
Vivantes Hospital Am Urban	Berlin<br><br>(Northeastern Germany)	21	2018	Metropolitan
Vivantes Hospital Neukölln	Berlin<br><br>(Northeastern Germany)	17	2018	Metropolitan
Charité – Berlin University Medicine	Berlin<br><br>(Northeastern Germany)	5	2020	Metropolitan
Centrum for Psychiatry Südwürttemberg- Clinic Zwiefalten	Baden-Württemberg (Southwest Germany)	15	2018	Rural
Centrum for Psychiatry Südwürttemberg-Clinic Weissenau	Baden-Württemberg (Southwest Germany)	10	2018	Urban/rural
Clinic for Psychiatry and Psychosomatics Reutlingen	Baden-Württemberg (Southwest Germany)	15	2018	Urban
Isar-Amper Hospital Munich	Bavaria (South Germany)	18	2018	Metropolitan
University Clinic Tübingen	Baden-Württemberg (Southwest Germany)	5	2019	Urban/rural
Centrum for Psychiatry - Clinic Reichenau	Baden-Württemberg (Southwest Germany)	15	2018	Urban/rural

IHT = intensive home treatment

© 2024 Bechdolf A et al. *JAMA Network Open*.
