# Supplementary material for: Utilization of Psychiatric Hospital Services Following Intensive Home Treatment: A Nonrandomized Clinical Trial
Source: JAMA Netw Open. 2024 Nov 15;7(11):e2445042. doi: 10.1001/jamanetworkopen.2024.45042 (PMC11568461; doi:10.1001/jamanetworkopen.2024.45042)
Supplement: Supplement 2. — Trial Protocol [file jamanetwopen-e2445042-s002.pdf]

Ethics Committee of the MHB

Tel. no.: 03391 39 3110

Mail: ethikkommission@mhb-fontane.de

Reason for the application: Medical study project

Rüdersdorf, 15.07.2020

Ethics Committee of the MHB

Fehrbelliner Straße 38 | House O

16816 Neuruppin

1. **Title and acronym of the research project**

Outreach crisis treatment with team-based and integrated care: Evaluation of inpatient equivalent home treatment (IEHT according to § 115d Social Code Book V) - a proof-of-concept study (AKtiV)

2. **Applicant**

Prof. Dr. Sebastian von Peter

3. **Contact details**

Immanuel Klinik Rüdersdorf, Psychiatric University Clinic of the Brandenburg Medical School Seebad 82/83, 15562 Rüdersdorf near Berlin

Phone:033638 83-501

E-Mail: Sebastian.vonPeter@mhb-fontane.de

4. Clinic, department, institution

Psychiatric University Clinic of the Brandenburg Medical School

5. Head of the clinic, department, institution

Prof. Dr. Martin Heinze, Psychiatric University Clinic of the Brandenburg Medical School

6. Cooperation partners/co-applicants involved

Module A: Dr. Johanna Baumgardt (scientific study director), Prof. Dr. Andreas Bechdolf (scientific study director), Vivantes Klinikum am Urban and Vivantes Klinikum im Friedrichshain, academic teaching hospitals Charité - Universitätsmedizin Berlin: Quantitative evaluation of patients and relatives

Module B: Prof. Dr. Sebastian von Peter (consortium leader), Dr. Julian Schwarz, University Clinic for Psychiatry and Psychotherapy Immanuel Klinik Rüdersdorf, Brandenburg Medical School: Qualitative outcome evaluation of users, relatives and employees

Module C1: Prof. Dr. Gerhard Längle, Zentrum für Psychiatrie Südwürttemberg, Klinik für Psychiatrie und Psychotherapie Zwiefalten; Gemeinnützige GmbH für Psychiatrie Reutlingen (PP.rt), academic teaching hospital of the University of Tübingen, Martin Holzke, Klinik für Psychiatrie und Psychotherapie I der Universität Ulm (Weissenau) am Zentrum für Psychiatrie Südwürttemberg: Routine data analysis and quantitative and qualitative process evaluation of StäB treatment

Module C2: Prof. Dr. Peter Brieger, Isar-Amper Klinikum München Ost: Routine data analysis and quantitative and qualitative process evaluation

Module D: Prof. Dr. Reinhold Kilian, University of Ulm: Health Economic Evaluation

Module E: Prof. Dr. Jürgen Timm, Competence Center for Clinical Studies Bremen, Department of Biometrics, University of Bremen

Study centers:

- Immanuel Klinik Rüdersdorf, Department of Psychiatry, Psychotherapy and Psychosomatics, Psychiatric University Clinic of the Brandenburg Medical School Theodor Fontane, Seebad 82/83, 15562 Rüdersdorf near Berlin
- Vivantes Klinikum Am Urban, Academic Teaching Hospital Charité - Universitätsmedizin Berlin, Department of Psychiatry, Psychotherapy and Psychosomatics, Dieffenbachstraße 1, 10967 Berlin

- Vivantes Klinikum Neukölln, Academic Teaching Hospital Charité - Universitätsmedizin Berlin, Clinic for Psychiatry, Psychotherapy and Psychosomatics, Rudower Straße 48, 12351 Berlin Neukölln
- Department of Psychiatry and Psychotherapy, Campus Charité Mitte, Charitéplatz 1, 10117 Berlin
- Center for Psychiatry Südwürttemberg, Clinic for Psychiatry and Psychotherapy Zwiefalten, Hauptstraße 9, 88529 Zwiefalten
- Isar-Amper-Klinikum München-Ost, teaching hospital of Ludwig-Maximilians-Universität München, Vockestraße 72, 85540 Haar near Munich
- Gemeinnützige GmbH für Psychiatrie Reutlingen (PP.rt), academic teaching hospital of the University of Tübingen, Wörthstr. 52/1, 72764 Reutlingen
- Clinic for Psychiatry and Psychotherapy I of the University of Ulm (Weissenau) at the Center for Psychiatry South Württemberg, Weingartshofer Str. 2, 88214 Ravensburg - Weissenau
- Center for Psychiatry Reichenau, Clinic for Social Psychiatry, academic teaching hospital of the University of Konstanz, Feursteinstraße 55, 78479 Reichenau
- University Hospital Tübingen and Medical Faculty of the University of Tübingen, Department of Psychiatry and Psychotherapy, Division of General Psychiatry and Psychotherapy, Calwerstr. 14, 72076 Tübingen

Description of the research project

7. Research question and study objective

The **overarching aim** of the proposed study is to investigate the implementation, treatment processes, clinical effectiveness and costs of inpatient equivalent home treatment (IEHT) in accordance with § 115d SCB V in comparison to conventional inpatient treatment from the perspective of users, relatives and practitioners. Using qualitative and quantitative methods of health services research as well as complementary data sources, it is aimed to create a robust, widely accepted evidence base for the further development of outreach, team-based forms of psychiatric treatment in the home environment as an alternative to inpatient care with the involvement of relevant interest groups and perspectives. The project objective is subdivided into sub-objectives, which are to be answered using methods of outcome-, process- and implementation-research as well as health economic evaluation.

Primary research question (outcome research): Are there differences in inpatient psychiatric readmission rates between inpatient-equivalent and inpatient-treated users over the 12-month period? The **primary working hypothesis** is that users who were treated using IEHT have a significantly lower inpatient psychi-

atric readmission rate 12 months after inclusion in the study than users who received conventional inpatient psychiatric treatment during the study period. Although the readmission rate is an imperfect indicator of treatment quality in psychiatry (Durbin et al. 2007), it can certainly be considered an indication of successful acute treatment, recovery and meeting needs in the community-psychiatric setting (Byrne et al. 2010; Olfson et al. 1999). In addition, this outcome parameter was used in most international studies on home treatment (DGPPN, 2019). In the present study, the full inpatient psychiatric readmission rate is supplemented by the combined readmission rate (full inpatient/partial inpatient/residential) and other outcome parameters. The following secondary research questions should also be answered in this study: Do the readmission rate (full inpatient + partial inpatient + inpatient), the total number of days spent in full inpatient psychiatric care, treatment dropouts decrease and do the health-related quality of life, psychosocial functioning level, occupational integration, treatment satisfaction, perceived involvement in treatment decisions and recovery orientation increase in users receiving inpatient equivalent treatment compared to users receiving full inpatient psychiatric care? In addition, the relatives of study patients will be asked about their treatment satisfaction and their experience of stress in the context of the respective form of care. The qualitative outcomes examined in this research project are the subjective experience of those affected, including relatives, and the benefits they perceive from IEHT. As part of a **health economic evaluation**, direct and indirect costs of the two forms of treatment will be compared and a cost-utility analysis carried out. **Process research** will analyze which target group benefits most from IEHT, which impact factors are associated with successful treatment processes, and whether and when admission should take place directly or from inpatient treatment. Due to the limited experience with outpatient treatment in Germany, the question of the target group for this form of home treatment in terms of diagnosis, severity of illness and psychosocial situation has not yet been clarified. The development, organization and satisfaction of the treatment teams, their impact on the target criterion and the impact on the regional care system (system effects) will be examined as part of the **implementation research**.

8. Study design

This project is not an efficacy study, but a multicenter proof-of-concept study. In this study, the above-mentioned form of treatment is examined with regard to clinical effects as well as subjective and care-related aspects. The study is divided into modules, each of which investigates different topics and questions using a mixed-methods design.

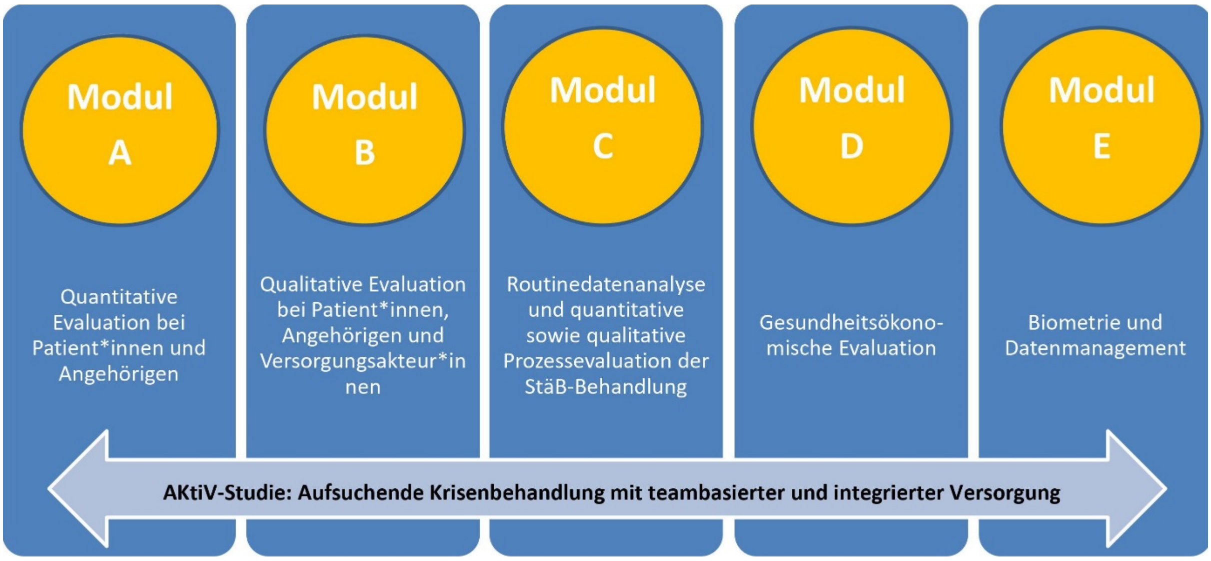

Fig. 1: Study design

Planned duration of study in months

The planned duration of the study is 36 months. A project schedule is attached (document "Timetable").

9. Type and duration of data collection and recruitment

The study participants will be recruited in ten study centers over a period of 12 months. Until the planned number of cases is reached, all IEHT patients will be asked to participate after being informed about the study. If consent is given and the inclusion criteria are met, the patients are included in the study (see 11.). Subsequently, each included person is matched with a patient who also fulfills the inclusion criteria according to the propensity score. The matched person is also informed in the same clinic and, if consent is given, is recruited as a control patient. After inclusion, a prospective survey of all patients is conducted at four points in time: Baseline = one week after admission and at the end of the index treatment, 1st follow-up = 6 months after admission, 2nd follow-up = 12 months after admission. Admission to IEHT or inpatient treatment is set as the time of admission. The survey data is supplemented by information from the attending physician and routine data from the individual clinics in accordance with §301 SCB V (e.g. for health economic analyses).

In addition, the relatives in the sense of a "person permanently living in the same household" of each study participant is asked about participation in the study. In the absence of close relatives, a close caregiver, such as a live-in caregiver, could also be approached regarding participation in the study.

The employees in the IEHT teams at the local study centers are surveyed using questionnaires on satisfaction, workload and team processes.

In addition to the quantitative survey of patients, relatives and staff, qualitative focus group and expert interviews will be conducted with a sample of IEHT patients and their relatives as part of a multi-modal, participatory research approach. A questionnaire survey of selected IEHT team members, experts and focus group participants is also planned.

10. Planned sample size

Quantitative:

N = 400 patients (= approx. 40 patients per study center): n = 200 patients each in the intervention group (IG) and in the control group (CG)

N = 400 relatives (= approx. 40 relatives per study center): n = 200 relatives each of patients in IG and in CG.

N = 100 - 150 IEHT employees (= approx. 10 - 15 IEHT employees per study center)

Qualitative: Planned are:

- 25 Participant observations (TB) (Kusenbach 2008) to investigate the implementation of IEHT as undistortedly as possible under real-life conditions in a participatory manner
- 40 individual interviews (EI) with users and relatives, which are recorded, transcribed and decoded using qualitative content analysis or grounded theory methodology (Patton 2015)
- 30 focus group interviews (FG) that were recorded, transcribed and decoded using qualitative content analysis (Mayring, 2015)
- Expert interviews are conducted with participants who have particular expertise. The interviews will be conducted using guidelines with sufficient space for the participants to set their own relevance; they will be transcribed and evaluated using qualitative content analysis. A total of approx. 30 EIs are planned across all the institutions studied. For Module C, an already tested interview guide will serve as a basis for the approx. 20 planned interviews (Heinsch, 2019).
- Discussion groups (DG) are held with employees in order to validate the preliminary results of the

patient and relatives survey in a trialogical manner, i.e. taking into account the perspective of employees.

11. Study population

All patients who are treated in the participating study centers by means of inpatient equivalent treatment according to §115d SCB V and who meet the inclusion criteria of the AKtiV study are addressed for study participation in the intervention group (IG):

- Criteria for admission to equivalent inpatient treatment:
  - Acute inpatient psychiatric crisis requiring treatment
  - Suitability of the home environment
  - Consent of the home environment
  - if underage children live in the household: No risk to the child's welfare
- Inclusion criteria of the AKtiV study:
  - No acute danger to self or others
  - Main diagnosis according to ICD 10 F0X, F1X, F2X, F3X, F4X, F5X, or F6X
  - Permanent residence in the service district of the respective study center
  - No placement order according to the respective state law
  - Capacity to consent
  - No severe organic brain diseases with cognitive deficits
  - no intellectual disability
  - Sufficient language skills for an interview in German
  - no participation in an intervention study at the time of study inclusion and during the index stay
  - Admission to the form of treatment was no longer than 7 days ago

For study participation in the control group (CG), all patients who receive regular inpatient treatment in the participating study centers, meet all inclusion criteria of the AKtiV study, meet all criteria for admission to inpatient-equivalent treatment if possible and have the most similar PS value to a study participant from the IG. Relatives to be interviewed must be at least 18 years of age, be named by the patient as the person to be interviewed, live permanently in the same household as the patient or have a comparable position in the patient's life (e.g. live-in caregiver) in order to participate in the study. In addition, employees of the respective study centers and relevant stakeholders from politics and self-administration in the care regions are interviewed.

All study participants will be informed in writing about the study and must agree to participate, including

data collection, storage and processing (see appendices). For this purpose, written consent will be obtained from each study participant (see appendices). Participation in the study can be revoked at any time without giving reasons. This is also recorded in the declaration of consent. Study patients and their relatives will be interviewed using an eCRF and the following validated special questionnaires.

12. Possible complications and risks

Physical and psychological risks that could be induced by the surveys and interviews are not to be expected. Participants are informed about the content and objectives of the survey and decide whether or not to take part in the interview or survey after being informed. Should psychological stress occur during the surveys and interviews, these would be paused or terminated completely. Research questions are asked sensitively and against the background of a trusting, appreciative attitude.

13. Dropout criteria

An interview situation can be terminated at any time at the request of the participants.

14. Risk-benefit assessment

No risks are expected within the scope of the study. The benefits of the study, on the other hand, are high, as the aim is to identify findings on the effectiveness of inpatient equivalent treatment compared to regular inpatient care. The results of the study should also facilitate the implementation process for clinics that do not yet offer inpatient equivalent treatment.

15. Further information on the methodology

<u>Module A</u>

The quantitative survey aims to answer the following questions: Are there differences between inpatient-equivalent treatment patients and those treated as full inpatients in terms of full inpatient psychiatric readmission rate (primary endpoint), combined readmission rate (full inpatient + partial inpatient + inpatient), number of full inpatient psychiatric treatment days, treatment drop-out rate, direct and indirect costs (see Module D), occupational integration, health-related quality of life, psychosocial functioning, occupational integration, recovery, treatment satisfaction and perceived involvement in treatment decisions (see Module C) (secondary endpoints)? In addition, the survey will analyze treatment satisfaction and the burden on relatives and compare it between the groups. The data collection to answer these questions will be carried out using the following, almost exclusively standardized, validated questionnaires:

	Indicator	Instrument
Patient	inpatient<br>Psychiatric readmission	1. routine data and German version of the Client Sociodemographic and Service Receipt Inventory (CSSRI) according to Chisholm et al. (2000) in the German translation (CSSRI-D) according to Roick et al. (2001)
	Continuity of treatment	2. CSSRI-D
	Quality of life	3. health-related quality of life using EQ5D-5L according to Jannsen et al (2013) in the German version according to Leidl & Reitmeir (2017)
	Social functioning level	4. course and severity of psychiatric symptoms in the inpatient setting with the Health of the Nation Outcome Scales (HoNOS) (Fankhauser, Hochstrasser, Sievers, & Soyka, 2017), Personal and Social Performance Scale PSP (Morosini, 2000)
	Professional integration	5. CSSRI-D with additional item
	Recovery orientation	6 Recovery Assessment Scale (RAS) (Corrigan et al., 1999) in the German translation (RAS-G) according to Cavelti et al. (2017)
	Evaluation and Utilization of care offered	7. CSSRI-D
	Treatment satisfaction	8. questionnaires developed by the authors
	Perceived involvement in treatment decisions	9. 9-item Shared Decision Making Questionnaire (SDM-Q-9) in the German translation according to Kriston et al. (2010)
Relative	Burden on relatives	10th Involvement Evaluation Questionnaire (IEQ-EU) in the German translation (Bernet et al., 2001)
	Treatment satisfaction	11. questionnaires developed by the authors

Questionnaires 1 to 7 are collected as part of the baseline survey (= within the first seven days after admission to the respective treatment setting) and then again 6 months (+/- 14 days) and 12 months (+/- 14 days) later. Questionnaires 8 to 11 are conducted at the end of the index episode (day of discharge +/- 7 days). The baseline survey is conducted in the patient's social environment (IG), in the clinic (CG) or by telephone (IG & CG). For the follow-up interviews, the study participants are contacted by telephone and then interviewed either by telephone, at home or in the clinic. If necessary, relatives or caregivers are included. In order to reduce the non-response rate and to be available for questions from study participants, interviews should take place face-to-face or in person wherever possible. In order to reduce the drop-out rate, patients participating in the quantitative survey will be paid an expense allowance of €50 after completion of the 12-month follow-up survey.

Module B

Module B1: Qualitative outcome evaluation of stakeholders

In this module, only hypothesis-generating or qualitative methods are used. The aim is to investigate stakeholders' experiences with IEHT, which is both beneficial and detrimental to good acute psychiatric treatment in the home environment. For this purpose, a participatory-collaborative research approach is chosen, which is an equal collaboration of researchers *with* and *without* experience expertise as users and/or relatives in psychiatry. The qualitative evaluation of the experiences of patients, relatives and care actors is carried out by means of focus groups (FG), expert interviews (EI) and participant observation (TB).

FGs are filled both trialogically (patients, relatives and staff) and by only one of these three groups. The sampling is structured according to a sampling plan (Patton, 2015). A total of ten to 15 FGs are planned across all the facilities studied. The exact number depends on the data saturation of the collected material. The FGs are recorded and transcribed.

EIs are conducted with users and relatives who have specific, practical expertise with IEHT. This includes, for example, users who have been treated for a long time, often or intensively, but also corresponding IEHT staff. The interviews were conducted using guidelines with sufficient space for the participants to set their own relevance; they were recorded, transcribed and pseudonymized. A total of 40 EIs are planned across all the institutions studied. The exact number depends on the data saturation of the collected material. TB is used in order to be able to depict everyday practices in the implementation of the IEHT as undistorted as possible (Kusenbach 2008). This approach, which originates from the repertoire of ethnographic field research, makes it possible to investigate the practice of IEHT and how it is experienced by users and relatives under real-life conditions. Five TBs are planned in at least five of the participating care

regions over the course of the project, i.e. a total of 25 TBs.

Module B2: - Extended stakeholder analysis on the relevance of the cross-sectoral aspect in IEHT

Sub-module B2 examines the extent to which the cross-sectoral and cross-jurisdictional consistency of IEHT has been implemented and what opportunities exist for its further development. As in module B1, hypothesis-generating, qualitative methods are also used in this module, including expert interviews (EI), focus groups (FG) and discussion groups (DG). Semi-quantitative data on cross-sectoral cooperation in individual model regions will also be collected. This data will be used to carry out a social network analysis, provided the data quality allows this. No users will be interviewed in this sub-project.

Extended stakeholders are defined as actors who are involved in the treatment and support of users both inside and outside the study clinics. In addition, relevant stakeholders from politics and self-administration in the care regions, as well as at state and federal level, are included in order to identify the potential for further development of the cross-sectoral aspect. To this end, the previous implementation of the cross-sector aspect in IEHT will be examined with regard to its potential for further development. The aim is to derive practical solutions and recommendations for strengthening cross-sectoral treatment within the framework of IEHT at both hospital and health system level.

Participants in the qualitative part will be paid an additional €50 to cover their expenses.

Module C

Module C1: evaluation of implementation and treatment processes across study-centers

Module C1 uses (1) routine data of the treatment processes in IEHT (§ 301 data set) (2) structural data of all participating study centers (3) additionally collected primary data on implementation, treatment processes, clinical history and sociodemographic data of the treated patients as well as (4) qualitative and quantitative primary data collected specifically from the treatment teams. (5) The treatment satisfaction of the patients (control and intervention group) is surveyed using a self-constructed questionnaire that has already been used in previous dissertations as part of the quantitative survey of Module A and evaluated by Module C. (6) The treatment satisfaction of the relatives (control and intervention group) is also surveyed using a self-constructed questionnaire, which has already been used in previous dissertations, as part of the quantitative survey of Module A and evaluated by Module C.

(1) With regard to the treatment processes, the routine data of the §301 data set is used on a person-

related basis, supplemented by the treatment data based on the OPS codes. The data in accordance with Section 301 SCB V contains the service and user data relevant for billing, which is made available to the payers. The data to be transmitted to the health insurance funds in accordance with Section 301 SCB V includes user and insured person data (age, gender), the place of service provision, general admission data as well as admission diagnoses, specialist department and follow-up diagnoses, therapy times according to individual professional groups (OPS coding) and finally also discharge data (reason for discharge, discharge diagnoses, etc.). The patient-specific routine data is linked with data from modules A and B (e.g. clinical history (number of previous inpatient psychiatric stays in the same clinic within the last 2 years); psychosocial background to treatment history). (2) In addition, primary data is collected on the individual access route to IEHT and the respective waiting time before admission to IEHT. In addition to the living environment and income, patient-specific data on the course of treatment (crisis interventions during treatment, transfer from inpatient to residential treatment and vice versa, interruption and discontinuation of treatment) are collected. This data is based, on the one hand, on the basic documentation of the individual centers and, on the other hand, on relevant parameters in the context of module C2 (type of admission to IEHT: directly or after transfer) as well as the respective hospital information systems (HIS) of the participating centers. (3) The structural data of the IEHT-practicing study centers are collected using a newly developed structural questionnaire, guideline-based expert interviews (Heinsch, 2019) and focus groups. Data collection using the structural questionnaire takes place at the same time as recruitment in the intervention group. The items contained in the structural questionnaire relate to the period of data collection or the previous year 2020. Relevant variables for the description and composition of the IEHT teams are queried. Of relevance are the team size and team composition by occupational group. To further describe the study centers, variables on general organization (on-call duty, interdisciplinary or specialist treatment, central vs. decentralized offices, etc.) and to record the structural conditions (type of clinic, description of the region, number of IEHT treatment places, maximum travel times, means of transport used, cooperation with other service providers, therapies offered) are used. The structural questionnaire is completed by senior members of the IEHT team. The information from the structural questionnaire is supplemented by (4) guided interviews and results from the focus groups. These focus on the timeline for implementation, difficulties encountered, details on staff recruitment and organization as well as changes in the process since the introduction of IEHT. In addition, the treatment processes are mapped by determining the type and content of the services in the focus groups and describing where and when they take place and who carries them out. The EIs are conducted with one or two people from each of the study centers' IEHT teams in order to record the implementation process and the current status. The focus groups are conducted with the IEHT employees at all study centers with the participation of as many professional groups of the individual teams as possible. The well-validated COPSOQ questionnaire (Burr et al.,

2019) will be used to record employee satisfaction and stress levels. In addition, selected CPAT (OIPEP, 2009) items translated into German by module employees (in consultation with the authors of the questionnaire) are used. These are supplemented by items from internal satisfaction questionnaires of the ZfP Südwürttemberg and finalized in the course of project phase 1 in joint coordination with the other study centers. An initial validation of the supplemented questionnaire will be carried out in a preliminary survey at study centers not participating in the study. (5) A questionnaire designed at the ZfP Südwürttemberg will be used to record the treatment satisfaction of patients (intervention and control group). This records the patients' satisfaction with the treatment on a 5-point Likert scale. It comprises a total of 18 items and four open questions. The questionnaire was established as part of two dissertation projects at the ZfP Südwürttemberg and the PP.rt Reutlingen (Götz, 2019; Hirschek, 2019). (6) A questionnaire designed at the ZfP Südwürttemberg is also used to record the treatment satisfaction of relatives (intervention and control group). This records the satisfaction of the relatives with the treatment on a 5-point Likert scale. It comprises a total of 13 items and one open question. The questionnaire was established as part of two dissertation projects at ZfP Südwürttemberg and PP.rt Reutlingen (Götz, 2019; Hirschek, 2019).

Module C2: Influence of the access route

In Module C2, patient characteristics (e.g. age, gender, diagnoses, level of functioning/severity of illness, structure of the social network, marital status, resources, previous psychiatric treatment) and treatment processes (e.g. team organization, frequency of contact between different professions, treatment discontinuations) are examined depending on the type of referral (direct admission / transfer to inpatient treatment from a ward). In addition (as a supplement to the patient treatment satisfaction surveyed in Module C1), the perceived involvement in medical decisions is to be surveyed comparatively for patients in inpatient and outpatient treatment. The SDM-Q-9 questionnaire in the validated German version according to Kriston et al. (2010) will be used for this purpose.

Module D

In Module D, a primary data-based cost-utility analysis (CUA) is conducted from an economic perspective using the net benefit method (Drummond, O'Brien, Stoddart, & Torrance, 1997; Glick, 2010; Salize & Kilian, 2010). The aim of the analysis is to estimate the necessary maximum willingness to pay (MWTP) for the gain of a life year in full health (QALY) by means of IEHT compared to TAU. The basis of the CUA from an economic perspective is the complete recording of the direct and indirect costs of illness and the measurement of the subjective quality of life of the study participants using a preference-based measurement method (Bernert et al., 2009; Brooks, Rabin, & Charro, 2003). Due to the different payers of mental health care services on the basis of Social Code Book (SCB) legislation, a complete assessment of the economic

consumption of resources is only possible by directly surveying the users (Beecham & Knapp, 2001; Salize & Kilian, 2010). For this purpose, the German version of the Client Socio-Demographic Service Receipt Inventory (CSSRI) is used to record the utilization of medical and psychosocial health services across the SCB as the basis for estimating the direct costs of illness (Chisholm et al., 2000; Roick et al., 2001). The cost of illness is calculated by multiplying the recorded service units by the respective costs of these service units (Bock et al., 2015; Grupp, König, & Konnopka, 2017; Salize & Kilian, 2010). Published data on unit costs in the German healthcare system (Bock et al., 2015; Grupp et al., 2017) are updated for this purpose through our own research as required. The preference-based measurement of subjective quality of life as the basis for the QALY estimate is carried out using the EQ5D-5L (Buchholz, Janssen, Kohlmann, & Feng, 2018; Janssen et al., 2013; Leidl & Reitmeir, 2017) on the basis of the current standard values for the German general population (Leidl & Reitmeir, 2017).

Module E

Module E accompanies all other modules with regard to all biometric concerns and issues throughout the entire duration of the project. The provisions of the GCP Regulation, the ICH Guidelines, data protection and the quality assurance Standard Operating Procedures (SOP) of the KKSB are implemented as follows:

a) **Creation of a data protection concept:** see 19.b

b) **Database setup, data management plan**: A data management plan will be drawn up containing technical details of the collection, distribution or transfer of data, storage and provision for analysis in accordance with the data protection concept. A database is set up for the study.

c) **Propensity score matching:** A suitable control case is determined for each IEHT case by means of propensity score matching**.**

   a. Preparation: A logistic regression analysis of the previous year's data of users who meet the inclusion criteria is carried out for each center. Participation in IEHT (YES/NO) is used as the outcome variable. Regressor variables are age group, gender, diagnosis (short form), number of previous inpatient stays at the respective study center in the past two years. For each center, the analysis provides a function for calculating the propensity score (PS) for given values of the regressor variables. In addition to these center-specific analyses, an analogous overall analysis of all evaluable cases is performed. If a center does not have sufficient data from the previous year, the PS function from this overall analysis is used in this center.

b. Matching at the center: An individual PS value is calculated for all potential control subjects using the PS function and listed according to increasing PS value (this list is updated regularly during the study). When a new IEHT case is included, its PS value is calculated using the PS function and compared with the PS list of potential control subjects. The user with the most similar PS value is included in the study as an associated control person (PS match) if informed consent is given and the inclusion criteria are met.

16. Planned evaluation methodology

Module A

The quantitative part of the study is analyzed in close collaboration with Module E (biometrics). First, descriptive statistical key figures are calculated for all baseline values. The regressor variables used for matching and the propensity score values are tested exploratively for homogeneity. Descriptive statistics are calculated for all progression parameters collected in the module. The difference in the follow-up data between IG and CG is analyzed on the basis of the following criteria (endpoints) in three stages (primary, secondary, tertiary) with the null hypothesis "no difference between IG and CG":

The **primary criterion** (deductive statistical test) is P1: The full inpatient psychiatric readmission rate within 12 months

The **secondary criteria** (exploratory tests, no alpha adjustment) are S1 to S7 after 6 and 12 months and S8 to S11 at the end of the index period (BK = baseline-corrected values):

S1: The combined readmission rate (inpatient + day-care + inpatient)

S2: The number of inpatient psychiatric treatment days within 12 months

S3: Discontinuation of inpatient psychiatric or equivalent treatment

S4: Quality of life (EQ5D-5L) Bk

S5: Psychosocial functioning level (separately for HoNOS and PSP, total score in each case) Bk

S6: Vocational integration (CSSRI-D) Bk

S7: Recovery orientation (RAS-G) Bk

S8: Treatment satisfaction (self-developed questionnaire) Bk

S9: Perceived involvement in decisions (SDM-Q-9) Bk

S10: Stressors (IEQ-EU) Bk

S11: Treatment satisfaction (self-developed questionnaire) Bk

Additionally secondary (but not processed in module A or E): lower direct and indirect costs.

Tertiary (further) evaluation: Explorative analysis of the influence of diagnosis groups, city-country, workplace, chronicity of the disease, type of treatment in addition to the classification according to IG and CG on the readmission rates.

Reference values from German or comparable populations are used to interpret the evaluation. For a differentiated description of the evaluation, see Module E.

Module B1

Focus groups and expert interviews are evaluated using qualitative content analysis. The participant observations are analyzed using grounded theory methodology. In the sense of triangulation, the results of the qualitative analysis are combined with the results of the quantitative approaches.

Module B2

A summarizing qualitative content analysis is carried out to identify the *status quo* of the cross-sector aspect in IEHT.

Module C1

The evaluation is carried out in close cooperation with Module E (biometrics). The focus is on comparing the structure, composition and organization of the treatment teams and their performance, as well as comparing the various treatment processes. The treatment provided to users is described using routine data on the treatment processes. Descriptive statistics are provided for all treatment parameters. These are compared between the individual study centers using the specific structural data of the individual centers. Descriptive statistics are again calculated by linking primary data from Module A on the effectiveness of the IEHT treatment at center level, which is described using the inpatient readmission rates and the number of days spent as an inpatient. Depending on the centre-specific organization of the IEHT team as autonomous or ward-integrated, the descriptive statistics of the effectiveness parameters are compared with each other at the different survey times. The parameters recorded on the satisfaction of employees, relatives and users of the individual centers are first described descriptively. Subsequently, correlations

are examined at the individual centers. Similarly, it is also examined whether the specific parameters describing IEHT treatment (proportion of individual professional groups involved in treatment, duration of treatment, etc.) show a stronger correlation with inpatient readmission rates than variables describing the users. If there are effects regarding the effectiveness parameters at the center level, it will be further investigated whether these can be explained by the different treatment processes at the centers rather than by variables of the different patient populations. A correlation analysis will also be conducted to test another assumption made in Module C1, namely that a low stability of the course during the index treatment is an unfavorable predictor of the risk of readmission. The change in the burden on relatives is also described descriptively between the two groups and examined using a correlation analysis.

The focus groups and expert interviews are evaluated using content analysis. In addition, the treatment processes identified in the focus groups are graphically depicted. It will be examined whether quality indicators for the implementation of IEHT can be derived in connection with patient satisfaction based on the Core Fidelity Scale, which was developed by Lloyd-Evans et al. (2016) for crisis resolution teams in the UK.

Module C2

Using the quantitative and qualitative data collected in modules A, B and C1, a comparison and analysis of direct admissions to hospital wards compared to transfers from wards will be carried out. In this way, better insights are to be gained into the indication for inpatient treatment and an improvement in process and possibly outcome quality. The work will be carried out as follows, with 1) preceding the parallel project phases 2) and 3):

1) **Identification of potential influencing factors that lead to direct admissions or diverting admissions (monocentric):** On the basis of 100 consecutively completed IEHT cases up to the start of the project, file studies will be used to extract which constellations (e.g. diagnoses, age, gender, pretreatment, admission indications) led to direct admissions and which led to diverting admissions. From this, a set of variables will be developed to underpin the prospective part of the study and which is suitable as a potential indicator to differentiate between acute and diverting admissions.

2) **Integration of these variables developed under 1) into project C1 (multicenter):** The variables identified in this way are to be reduced to a few core variables that are recorded with less complex basic documentation in all study centers and are not part of Section 301 (3) SCB V. At the same time, each case in the study centers is coded in such a way that it is clear whether it is a direct or diverting admission. This means that the variables obtained are checked on a large N and evaluated centrally.

3) **Examination of the variables developed under 1) (monocentric):** In the Munich center, the variables identified under 1) are examined in a differentiated manner. For this purpose, 140 consecutively recorded IEHT users will be compared to determine whether they were direct or external recordings. These empirical variables are supplemented by qualitative interviews at the Munich study center. In addition to the users, their relatives will also be included in the study. Finally, the treatment team will also be examined in terms of their experiences with the respective forms of treatment.

4) Due to the Center's long tradition of research on the topic of "participatory decision-making", the multicenter measurement of patient satisfaction in Module 1 will also be supplemented with a questionnaire on shared decision making (SDM-Q-9) in order to examine whether treatment decisions are made in a more participatory manner at the IEHT than under inpatient conditions.

Module D

As a statistical adjustment of the cost-benefit ratios is necessary due to the lack of randomization of the study participants, the estimation is carried out using net benefit regression models (Hoch, Briggs, & Willan, 2002; Icks et al, 2010; Salize & Kilian, 2010; Willan & Briggs, 2006; Willan, Briggs, & Hoch, 2004) and a propensity score adjustment (Austin, 2011; Kilian et al, 2016; Kilian et al, 2018). In this method, individual net benefit values (NMB) are first calculated using the formula

$NMBi = QALYi*\lambda-Ci$

where:

QALYi = quality adjusted life years of person i,

λ = threshold value for maximum willingness to pay Ci =
direct medical costs of person i.

The NMBi represents the individual monetary net benefit of the healthcare services used by person i, assuming a maximum willingness to pay λ for the gain of an additional year of life in full health (QALY). In contrast to the incremental cost-utility ratio (ICUR), the NMB is an individual measure with a parametric stochastic distribution that enables statistical analysis using multivariate regression models and thus adjustment for confounding variables to control selection bias (Hoch et al., 2002; Salize & Kilian, 2010). The propensity score for bias control is generated by estimating the conditional probabilities of belonging to

the intervention group (IEHT), based on a logistic regression model with study group membership as the dependent variable and potentially bias-relevant clinical and sociodemographic characteristics as independent variables (Austin, 2011). Propensity scores represent the individual probabilities of belonging to the intervention group, taking into account individual characteristics of bias-relevant characteristics, and therefore enable the statistical adjustment of group differences for the entirety of the characteristics included. Propensity scores can be used for both matching-based and regression-based methods of bias control (Austin, 2011). For the planned study, the method of regression-based bias control with the PS value is used. Due to the expected deviations in the distributions of the net benefit values, the stochastic uncertainty is estimated using non-parametric bootstrapping (Willan & Briggs, 2006). Based on the current discussion on the choice of threshold values for the maximum willingness to pay (Marseille, Larson, Kazi, Kahn, & Rosen, 2015; Woods, Revill, Sculpher, & Claxton, 2016), threshold values of €0, €25,000, €50,000, €75,000 and €100,000 are used for the net benefit regressions. Further differentiation of the thresholds is possible at any time.

Module E

Statistical analysis plan (SAP): Before data collection is completed, a statistical analysis plan is drawn up which, based on agreements with the modules, clarifies in detail which quantitative research questions and hypotheses of the individual modules of the study are to be processed in Module E using which statistical methods and data. The statistical analysis focuses on the data from Module A. The statistical analysis plan specifies the details for the statistical analysis and defines transformations (conversions) and, if necessary, imputations (substitute values) for missing values. The basic principles of the statistical analysis on which the SAP is to be based are described below. They are coordinated with the quantitative module and refer to the presentation of the evaluation there: All quantitative data recorded is evaluated descriptively. For categorized variables, the individual categories for IG and CG are counted separately and in total, and percentages within these groups are calculated. In the case of quantitatively recorded parameters, N (number of evaluable data), mean, standard deviation, median, minimum and maximum are also calculated separately for IG and CG and in total. The balance between IG and CG achieved with PS matching is tested with the PS regressor variables (of the logistic regressions) and the PS value.

- The primary question is to be answered deductively; for this purpose, the primary criterion is tested with a significance level of 5% (two-sided tests). The null hypothesis "no difference between IG and CG" is tested in the form "pairwise differences of the matched pairs are symmetrically distributed around 0". For this purpose, a McNemar test is used, which refers to the values of the matched pairs as connected samples.

- Secondary criteria are only tested exploratively with alpha=5%. The differences of the matched pairs are also used as a basis. The admissibility of the normal distribution assumption is checked in advance. If this assumption is valid, a paired t-test is used, otherwise the Wilcoxon signed rank test is used. For the secondary criterion S2, the time until discontinuation and, for cases without discontinuation (censor cases), the time of observation (maximum duration of 12 months) is defined as the value. The baseline correction (BK) is carried out by subtracting the baseline for each user individually.

- Tertiary (further) questions regarding the influence of important parameters are exploratively processed with a summarizing logistic regression for the target variable readmission (YES/NO) and the corresponding variables as regressors. Descriptive subgroup results are provided for significant influences.

The planned interlocking with the results of other modules, in particular the qualitative results, is described in the SAP process.

Statistical evaluation, reporting: The statistical evaluation is carried out using SAP, the calculations using the SAS program system. The results are sent to the respective module as a statistical report. The final report and subsequent publications are supported with statistical calculations.

Quality assurance: Plausibility check query management, checking the correctness of transmission, statistical programs, results and reports.

17. Has an application with the same content already been submitted to another ethics committee?

No

18. Information on recording, processing, storage and deletion of data

a) **Personal data:** The following socio-demographic data is collected as part of the study: Age, gender, signs of diagnosis, duration of treatment in years, number of inpatient and IEHT stays, previous experience with home treatment.

b) **Data protection:** The confidential information collected as part of the study is subject to medical confidentiality and the provisions of the European General Data Protection Regulation, the German Federal Data Protection Act and the German Social Security Code. A data protection concept is available for the handling of data in the study, which is decisive and binding for the conduct of the study. The data collected as part of the study is recorded and evaluated exclusively in pseudonymized form, i.e. the data

records contain no information on names and dates of birth and are kept under a neutral number. It is not possible for the analyzing scientists to draw conclusions about the individuals. Only the attending physician can assign the number of the data record to a person using a list stored confidentially in the clinic. Once the data analysis has been completed, this list will be destroyed and it will no longer be possible to assign it to individual persons.

Research diaries, interview transcripts and transcripts of the group discussion are also not recorded, processed and evaluated on a personal basis, but pseudonymized. The interviews are conducted, transcribed and formalized by a third person. During the formalization of the interviews, all personal data is eliminated. The formalized outcomes with the corresponding identification numbers are forwarded to the evaluating employee for statistical analysis. The collected data and the transcripts are stored password-protected on the designated study computers of the participating study centers. The results of the survey and audio files as well as consent forms are stored separately.

After completion of the study, all data will be archived in accordance with the currently valid regulations, stored for 10 years and then completely deleted. This also includes the destruction of data on paper, such as questionnaires. The audio files will be deleted after transcription.

The pseudonymized data will only be evaluated by the participating scientists for the scientific purposes of the study. The confidentiality of personal data is guaranteed even if the results of the study are published. The proper conduct of the study, in particular the proper collection of data and its allocation to specific groups of participants, is checked by the data protection officers of the modules. Data subjects have the right to withdraw their consent at any time and to inspect their data collected during the study. If they discover errors in their data, they have the right to have these corrected by the module managers.

c) **Confidentiality / obligation to maintain data secrecy / confidentiality:** Researchers are obliged to maintain data secrecy and are subject to a duty of confidentiality. The researchers are released from the duty of confidentiality for data that does not allow the person to be identified. The recordings and transcripts are not published and are only accessible within the project. The pseudonymized transcripts are only read by persons who are involved in the evaluation and are also subject to confidentiality. Content may be included in publications in the form of a case presentation and individual quotations, naturally without the person being identifiable.

d) **Coding list and personal code word:** The coding list is created by the study employee during the pseudonymization of the study participants with the help of the neutral numbers and is deleted after the evaluation of the data.

e) **Deletion of the data:** Participation can be revoked at any time once consent has been given. Audio files can be deleted at any time. It is not possible to delete data that has already been recorded at a later date, as transcripts can no longer be assigned to individuals. The questionnaires and transcripts are archived for ten years after the end of the study and then destroyed. The audio files are deleted after transcription.

References

Austin, P. C. (2011). An introduction to propensity score methods for reducing the effects of confounding in observational studies. *Multivariate behavioral research*, *46*(3), 399-424.

Beecham, J., & Knapp, M. (2001). Costing psychiatric interventions. *Measuring mental health needs*, *2*, 200-224.

Benchimol, E. I., Smeeth, L., Guttmann, A., Harron, K., Moher, D., Petersen, I., & RECORD Working Committee. (2015). The REporting of studies Conducted using Observational Routinely-collected health Data (RECORD) statement. *PLoS Med*, *12*(10), e1001885.

Bernert, S., Fernández, A., Haro, J. M., König, H. H., Alonso, J., Vilagut, G., ... & Anger- meyer, M. C. (2009). Comparison of different valuation methods for population health status measured by the EQ-5D in three Euro- pean countries. *Value in health*, *12*(5), 750-758.

Bernert, S., Kilian, R., Matschinger, H., Mory, C., Roick, C., & Angermeyer, M. C. (2001). The assessment of burden on relatives of mentally ill people: the German version of the involvement evaluation question- naire (IEQ-EU). *Psychiatrische Praxis*, *28*, S97.

Blettner, M., Dierks, M. L., Donner-Banzhoff, N., Hertrampf, K., Klusen, N., Köpke, S., ... & Sundmacher, L. (2018). Überlegungen des Expertenbeirats zu Anträgen im Rahmen des Innovationsfonds. *Zeitschrift für Evidenz, Fortbildung und Qualität im Gesundheitswesen*, *130*, 42-48.

Bock, J. O., Brettschneider, C., Seidl, H., Bowles, D., Holle, R., Greiner, W., & König, H. H. (2015). Ermittlung standardisierter Bewertungssätze aus gesellschaftlicher Perspektive für die ge- sundheitsökonomische Evaluation. *Das Gesundheitswesen*, *77*(01), 53-61.

Brooks, R., Rabin, R., & de Charro, F. (Eds.). (2003). *The Measurement and Valuation of Health Status Using*

EQ-5D: A European Perspective: Evidence from the EuroQol BIOMED Research Programme. New York: Springer Science & Business Media.

Deutscher Bundestag (2016a). *BT-Drucksache 18/9528 vom 05.09.2016- Gesetzentwurf der Bundesregierung zur Weiterentwicklung der Versorgung und der Vergütung für psychiatrische und psychosomatische Leistungen (PsychVVG) 2018a.* Verfügbar unter http://dipbt.bundestag.de/dip21/btd/18/095/1809528.pdf [03.05.2018].

Deutscher Bundestag (2016b). *BT-Drucksache 18/10289 vom 09.11.2016 - Beschlussempfehlung und Bericht des Ausschusses für Gesundheit (14. Ausschuss) 2018b*. Verfügbar unter http://dip21.bundestag.de/dip21/btd/18/102/1810289.pdf [03.05.2018].

Buchholz, I., Janssen, M. F., Kohlmann, T., & Feng, Y. S. (2018). A systematic review of studies comparing the measurement properties of the three-level and five-level versions of the EQ-5D. *Pharmacoeconomics*, *36*(6), 645-661.

Bühring, P. (2017). Stationsäquivalente psychiatrische Behandlung: Neues Element zur Flexibilisierung. *Deutsches Ärzteblatt*, 114(46), A-2132 / B-1795 / C-1753.

Burr, H., Berthelsen, H., Moncada, S., Nübling, M., Dupret, E., Demiral, Y., ... & Lincke, H. J. (2019). The third version of the Copenhagen Psychosocial Questionnaire. *Safety and Health at Work*, *10*(4), 482-503.

Byrne, S. L., Hooke, G. R., & Page, A. C. (2010). Readmission: a useful indicator of the quality of inpatient psychiatric care. *Journal of affective disorders*, *126*(1- 2), 206-213.

Cargo, M., & Mercer, S. L. (2008). The value and challenges of participatory research: strengthening its practice. *Annu. Rev. Public Health*, *29*, 325-350.

Cavelti, M., Wirtz, M., Corrigan, P., & Vauth, R. (2017). Recovery assessment scale: examining the factor

structure of the German version (RAS-G) in people with schizophrenia spectrum disorders. *European psychiatry*, *41*, 60-67.

Chisholm, D., Knapp, M. R. J., Knudsen, H. C., Amaddeo, F., Gaite, L. U. I. S., Van Wijngaarden, B. O. B., & EPSILON Study Group. (2000). Client socio-demographic and service receipt inventory–European version: development of an instrument for international research: EPSILON Study 5. *The British Journal of Psychiatry*, *177*(S39), s28-s33.

Corrigan, P. W., Giffort, D., Rashid, F., Leary, M., & Okeke, I. (1999). Recovery as a psychological construct. *Community mental health journal*, *35*(3), 231-239.

De Silva, M. J., Breuer, E., Lee, L., Asher, L., Chowdhary, N., Lund, C., & Patel, V. (2014). Theory of change: a theory-driven approach to enhance the Medical Research Council's framework for complex interventions. *Trials*, *15*(1), 267.

Deutsche Krankenhausgesellschaft, GKV-Spitzenverband & Verband der Privaten Krankenversicherung. (2017). *Vereinbarung zur Stationsäquivalenten Behandlung nach § 115d Abs. 2 SGB V*. Verfügbar unter https://www.dkgev.de/media/file/58271.Vereinbarung_stationsaequivalente_Behandlung_Anlage.pdf [12.06.2018].

Deutsche Krankenhausgesellschaft (2017). *Umsetzungshinweise der Deutschen Krankenhausgesellschaft zur Vereinbarung der Stationsäquivalenten Behandlung nach §115d Absatz 2 SGB V sowie ergänzende Informationen*. Verfügbar unter https://www.dkgev.de/fileadmin/default/Mediapool/2_Themen/2.3_VersorgungStruktur/2.3.8._PsychiatriePyschosomatik/2.3.8.2._Sta tionsaequivalente_psychiatrische_Behandlung/Umsetzungshinweise_stationsaequivalente_Behandlung.pdf [21.08.2019].

DIMDI (2018). *OPS Version 2018*. Verfügbar unter https://www.dimdi.de/static/de/klassifikationen/ops/kode-suche/opshtml2019/ [21.08.2019].

DGPPN (2018). *Gemeinsames Eckpunktepapier zur Stationsäquivalenten Behandlung (StäB)*.

DGPPN (2019). *S3- Leitlinie Psychosoziale Therapien bei schweren psychischen Erkrankungen*. Berlin, Heidelberg: Springer.

Drummond, M. F., Sculpher, M. J., Claxton, K., Stoddart, G. L., & Torrance, G. W. (1997). *Methods for the economic evaluation of health care programmes*. Oxford university press.

Durbin, J., Lin, E., Layne, C., & Teed, M. (2007). Is readmission a valid indicator of the quality of inpatient psychiatric care? *The journal of behavioral health services & research*, *34*(2), 137-150.

Fankhauser, S., Hochstrasser, B., Sievers, M., & Soyka, M. (2017). Assessing Change of Depressive Symptoms and Severity of Depression in an Inpatient Setting: Performance of the HoNOS (Health of the Nation Outcome Scales). *Psychotherapie, Psychosomatik, Medizinische Psychologie*, *67*(9-10), 391-400.

Fenton, W. S., Mosher, L. R., Herrell, J. M., & Blyler, C. R. (1998). Randomized trial of general hospital and residential alternative care for patients with severe and persistent mental illness. *American Journal of Psychiatry*, *155*(4), 516-522.

Flick, U. (2011). *Triangulation. Eine Einführung*. Wiesbaden: VS Verlag für Sozialwissenschaften.

Frasch, K. (2018). Stationsäquivalente Behandlung (StäB)–Ein großer Schritt in die richtige Richtung–Kontra. *Psychiatrische Praxis*, *45*(03), 123-124.

GKV (2017). *Stationsäquivalente psychiatrische Behandlung*. Verfügbar unter https://www.gkv- spitzenverband.de/krankenversicherung/krankenhaeuser/psychiatrie/stationsaequiv_psych_behandlung/st_aequ_beh.jsp [21.01.2019].

Götz, E. (2019). *Umsetzung der stationsäquivalenten Behandlung im städtischen Raum, Pilotstudie zur Implementierung einer neuen Versorgungsform in der Psychiatrie*. (Dissertation in Ausarbeitung).

Glick, H. A. (2010). *Economic evaluation in clinical trials* (Reprinted.). Handbooks in Health Economic Evaluation Series. Oxford: Oxford University Press.

Grupp, H., König, H. H., & Konnopka, A. (2017). Kostensätze zur monetären Bewertung von Versorgungsleistungen bei psychischen Erkrankungen. *Das Gesundheitswesen*, *79*(01), 48-57.

Heinsch, A. (2019). *Evaluation der Implementierungs- und Behandlungsprozesse der stations- äquivalenten Behandlung am ZfP Südwürttemberg und der PP.rt Reutlingen.* (Masterarbeit in Ausarbeitung).

Hirschek, D. (2019). *Umsetzung der stationsäquivalenten Behandlung im ländlichen Raum, Pilotstudie zur Implementierung einer neuen Versorgungsform in der Psychiatrie*. (Dissertation in Ausarbeitung).

Hoch, J. S., Briggs, A. H., & Willan, A. R. (2002). Something old, something new, something borrowed, something blue: a framework for the marriage of health econometrics and cost-effectiveness analysis. *Health economics*, *11*(5), 415-430.

Hollstein, B., Straus, F. (2006). *Qualitative Netzwerkanalyse: Konzepte, Methoden, Anwendungen*. Wiesbaden: VS Verlag für Sozialwissenschaften.

Hoult, J., Reynolds, I., Charbonneau-Powis, M., Weekes, P., & Briggs, J. (1983). Psychiatric hospital versus community treatment: the results of a randomised trial. *Australian and New Zealand Journal of Psychiatry*, *17*(2), 160-167.

Icks, A., Chernyak, N., Bestehorn, K., Brüggenjürgen, B., Bruns, J., Damm, O., & Greiner, W. (2010). Methods of Health Economic Evaluation for Health Services Research. *Das Gesundheitswesen*, *72*(12), 917-933.

Janssen, M. F., Pickard, A. S., Golicki, D., Gudex, C., Niewada, M., Scalone, L., & Busschbach, J. (2013). Measurement properties of the EQ-5D-5L compared to the EQ-5D-3L across eight patient groups: a multi-country study. *Quality of Life Research*, *22*(7), 1717-1727.

Johnson, S., Nolan, F., Pilling, S., Sandor, A., Hoult, J., McKenzie, N., & Bebbington, P. (2005). Randomised controlled trial of acute mental health care by a crisis resolution team: the north Islington crisis study. *Bmj*, *331*(7517), 599.

Kilian, R., Becker, T., & Frasch, K. (2016). Effectiveness and cost-effectiveness of home treatment compared with inpatient care for patients with acute mental disorders in a rural catchment area in Germany. *Neurology, Psychiatry and Brain Research*, *22*(2), 81-86.

Kilian, R., Frasch, K., Steinert, T., Schepp, W., Weiser, P., Jaeger, S., ... & Längle, G. (2018). Cost-effectiveness of psychotropic polypharmacy in routine schizo- phrenia care. Results of the ELAN prospective observational trial. *Neurology, Psychiatry and Brain Research*, *30*, 47-55.

Kriston, L., Scholl, I., Hölzel, L., Simon, D., Loh, A., Härter, M. (2010). The 9-item Shared Decision Making Questionnaire (SDM-Q-9). Development and psychometric properties in a primary care sample. Patient Educ Couns, 80(1),94-9.

Kusenbach, M. (2008). Mitgehen als Methode Der» Go-Along «in der phänomenologischen Forschungspraxis. In *Phänomenologie und Soziologie* (pp. 349-358). Wiesbaden: VS Verlag für Sozialwissenschaften.

Längle, G. (2018). Stationsäquivalente Behandlung (StäB)–Ein großer Schritt in die richtige Richtung–Pro. *Psychiatrische Praxis*, *45*(03), 122-123.

Längle, G., Holzke, M., & Gottlob, M. (2019). *Psychisch Kranke zu Hause versorgen: Handbuch zur Stationsäquivalenten Behandlung*. Stuttgart: Kohlhammer Verlag.

Leidl, R., & Reitmeir, P. (2017). An experience-based value set for the EQ-5D-5L in Germany. *Value in Health*, *20*(8), 1150-1156.

Lloyd-Evans, B., Bond, G. R., Ruud, T., Ivanecka, A., Gray, R., Osborn, D., & Kelly, K. (2016). Development of a measure of model fidelity for mental health Crisis Resolution Teams. *BMC psychiatry*, *16*(1), 427.

Marseille, E., Larson, B., Kazi, D. S., Kahn, J. G., & Rosen, S. (2015). Thresholds for the cost–effectiveness of interventions: alternative approaches. *Bulletin of the World Health Organization*, *93*, 118-124.

Mayring, P. (2015). *Qualitative Inhaltsanalyse: Grundlagen und Techniken*. 12. Aufl. Weinheim: Beltz.

McCrone, P., Johnson, S., Nolan, F., Pilling, S., Sandor, A., Hoult, J., & Bebbington, P. (2009). Economic evaluation of a crisis resolution service: a randomised controlled trial. *Epidemiology and Psychiatric Sciences*, *18*(1), 54-58.

McCrone, P., Johnson, S., Nolan, F., Sandor, A., Hoult, J., Pilling, S., & McKenzie, N. (2009). Impact of a crisis resolution team on service costs in the UK. *Psychiatric Bulletin*, *33*(1), 17-19

Morosini, P. L., Magliano, L., Brambilla, L., Ugolini, S., & Pioli, R. (2000). Development, reliability and acceptability of a new version of the DSM-IV Social and Occupational Functioning Assessment Scale (SOFAS) to assess routine social funtioning. *Acta Psychiatrica Scandinavica*, *101*(4), 323-329.

Murphy, S., Irving, C. B., Adams, C. E., & Driver, R. (2012). Crisis intervention for people with severe mental illnesses. *Cochrane Database of Systematic Reviews*, (5).

National Collaborating Centre for Mental Health. (2014). Psychosis and schizophrenia in adults: treatment and management. *NICE clinical guideline*, *178*, 1-59.

OIPEP (2009). Collaborative Practice Assessment Tool (CPAT). Property of the Office of Inter-professional Education and Practice, Queen's University.

Olfson, M., Mechanic, D., Boyer, C. A., Hansell, S., Walkup, J., & Weiden, P. J. (1999). Assessing clinical predictions of early rehospitalization in schizophrenia. *The Journal of nervous and mental disease*, *187*(12), 721-729.

Paton, F., Wright, K., Ayre, N., Dare, C., Johnson, S., Lloyd-Evans, B., & Meader, N. (2016). Improving outcomes for people in mental health crisis: a rapid synthesis of the evidence for available models of care. *Health Technologyl Assessment*, *20*(3).

Patton, M.Q, (2015). *Qualitative Re- search and Evaluation Methods*. 4. Aufl. Thousand Oaks, California: Sage Publications Ltd.

Proctor, E., Silmere, H., Raghavan, R., Hovmand, P., Aarons, G., Bunger, A., & Hensley, M. (2011). Outcomes for implementation research: conceptual distinctions, measurement challenges, and research agenda. *Administration and Policy in Mental Health and Mental Health Services Research*, *38*(2), 65-76.

Richards, D. A., & Hallberg, I. R. (Eds.). (2015). *Complex interventions in health: an overview of research methods*. Routledge.

Rifkin, S. B. (2014). Examining the links between community participation and health outcomes: A review of the literature. *Health Policy and Planning*, *29*, 98-106.

Roick, C., Kilian, R., Matschinger, H., Bernert, S., Mory, C., & Angermeyer, M. C. (2001). Die deutsche Version des Client Sociodemographic and Service Receipt Inventory. *Psychiatrische Praxis*, *28*(Sup. 2), 84-90.

Roick, C., Deister, A., Zeichner, D., Birker, T., König, H. H., & Angermeyer, M. C. (2005). The regional budget for mental healthcare: a new approach to combine inpatient and outpatient care. *Psychiatrische Praxis*, *32*(4), 177.

Sachverständigenrat für die Begutachtung der Entwicklung im Gesundheitswesen (2019). *Kurzfassung des Gutachtens 2018: Bedarfsgerechte Steuerung der Gesundheitsversorgung.*

Salize, H. J., & Kilian, R. (2010). *Gesundheitsökonomie in der Psychiatrie: Konzepte, Methoden, Analysen*. Stuttgart: Kohlhammer Verlag.

Sozialgesetzbuch (SGB) *Fünftes Buch- Gesetzliche Krankenversicherung.* Verfügbar unter https://de-jure.org/gesetze/SGB_V [09.08.2019].

Statistisches Bundesamt (2019). *Einrichtungen, Betten und Patientenbewegung 2017*. Verfügbar unter https://www.destatis.de/DE/Themen/GesellschaftUmwelt/Gesundheit/Krankenhaeuser/Tabellen/krankenhaeuser-fa.html.

Steinhart, I., & Wienberg, G. (2017). *Rundum ambulant. Funktionales Basismodell psychiatrischer Versorgung*

in der Gemeinde. Köln: Psychi- atrie-Verlag.

Thornicroft, G., & Tansella, M. (2003). What are the arguments for community-based mental health care. *Copenhagen: WHO regional Office for europe*.

von Peter, S. (2017). Partizipative und kollaborative Forschungsansätze in der Psychiatrie. *Psychiatrische Praxis*, *44*(08), 431-433.

Weinmann, S., Gühne, U., Kösters, M., Gaebel, W., & Becker, T. (2012). Teambasierte Gemeindepsychiatrie. *Der Nervenarzt*, *83*(7), 825-831.

Widmann, F., Bachhuber, G., Riedelsheimer, A., Schiele, A., Ullrich, S., Kilian, R., & Frasch, K. (2016). Home treatment. *Fortschritte der Neurologie· Psychiatrie*, *84*(01), 42-49.

Willan, A. R., & Briggs, A. H. (2006). *Statistical analysis of cost-effectiveness data* (Vol. 37). John Wiley & Sons.

Willan, A. R., Briggs, A. H., & Hoch, J. S. (2004). Regression methods for covariate adjustment and subgroup analysis for non-censored cost-effectiveness data. *Health economics*, *13*(5), 461-475.

Wirtz, M. A., Morfeld, M., Glaesmer, H., & Brähler, E. (2018). Normierung des SF-12 Version 2.0 zur Messung der gesundheits-bezogenen Lebensqualität in einer deutschen bevölkerungs-repräsentativen Stichprobe. *Diagnostica*, *64*(4), 215-226.

Wittchen, H. U., Jacobi, F., Rehm, J., Gustavsson, A., Svensson, M., Jönsson, B., & Fratiglioni, L. (2011). The size and burden of mental disorders and other disorders of the brain in Europe 2010. *European neuropsychopharmacology*, *21*(9), 655-679.

Woods, B., Revill, P., Sculpher, M., & Claxton, K. (2016). Country-level cost-effectiveness thresholds: initial estimates and the need for further research. *Value in Health*, *19*(8), 929-935.

Wright, M. T., Nöcker, G., Pawils, S., & Walter, U. (2013). Partizipative Gesundheitsforschung–ein neuer Ansatz für die Präventionsforschung. *Prävention und Gesundheitsförderung*, *3*(8), 119-121.

Signature of Director of Studies

Prof. Dr. med. Sebastian von Peter, MPhil

Rüdersdorf, the .09.2020
