# Supplementary material for: Utilization of Psychiatric Hospital Services Following Intensive Home Treatment: A Nonrandomized Clinical Trial
Source: JAMA Netw Open. 2024 Nov 15;7(11):e2445042. doi: 10.1001/jamanetworkopen.2024.45042 (PMC11568461; doi:10.1001/jamanetworkopen.2024.45042)
Supplement: Supplement 3. — Statistical Analysis Plan [file jamanetwopen-e2445042-s003.pdf]

STATISTICAL ANALYSIS PLAN

Clinical study

Outreach crisis treatment with team-based and integrated care: Evaluation of inpatient equivalent home treatment (IEHT according to § 115d Social Code Book V) - a proof-of-concept study

AKtiV

Supported by the Joint Federal Committee Innovation Fund

Kompetenzzentrum für Klinische Studien Bremen

Competence Center for Clinical Studies - Biometrics

Author: Lasse Fischer

Reviewed and approved by: Prof. Dr. Dr. h.c. Jürgen Timm & Paula Boskamp

Date: 28.03.2022 Version: Final No. 2

1. Table of contents

1. Table of contents 2
2. Introduction 9
2.1. Background 9
2.2. Study objective 10
2.3. References to the curriculum 12
2.4. Amendments 12
3. Study management 13
3.1. Applicant/consortium management 13
3.2. Scientific project management 13
3.3. Biometrics 14
4. Signatures 15
5. List of abbreviations 16
6. General specifications 18
6.1. Hardware and software used 18
6.2. Reporting standards and reporting language 18
6.3. General format of table, figure and list contents 18
6.4. Quality control 19
7. Details of the study design 20
7.1. General information on the design 20
7.1.1. Study population and selection of study participants 21
7.1.2. Termination criteria 21
7.1.3. Intervention 22
7.1.4. Consideration of the pre-treatment time in the definition of the index treatment 22
7.1.5. Planned treatment regimen and duration of treatment 23

7.1.6. Number of cases 23
7.2. Evaluation criteria 24
7.2.1. Demographic data and other baseline parameters 25
7.2.2. Treatment parameters 26
7.2.3. Tertiary variables 26
7.2.4. Auxiliary variables 27
7.2.5. Employee variables 27
7.2.6. Assessment of effectiveness 28
7.2.6.1. Primary target variable 28
7.2.6.2. Secondary and other target variables 28
7.2.7. Assessment of safety 29
8. Dealing with protocol violations 30
9. Evaluation collectives 31
9.1. Safety collective (SC) 31
9.2. Full analysis set (FAS) or intention-to-treat collective 31
9.3. Per-protocol collective 31
9.4. IEHT collective 31
9.5. IEHTPP collective 31
9.6. Employee collective 32
10. Data handling 33
10.1. Dealing with missing data, outliers and implausibility 33
10.1.1. Multiple imputation of missing data 33
10.1.1.1. Multiple imputation of questionnaires at the 12-month follow-up appointment 36
10.1.1.2. Multiple imputation of questionnaires at the end of the index treatment 36
10.1.1.3. Multiple imputation of the resumption 37
10.1.1.4. Combination of data sets analyzed using the Mann-Whitney test 38

10.1.2. Variation of the time intervals until the follow ups 38

10.2. Dealing with index handling aborts and drop-outs 39

10.3. Data transformation 39

10.3.1. Demographic data and other baseline parameters 39

10.3.1.1. Treatment group 39

10.3.1.2. Recording mode 39

10.3.1.3. Diagnosis 40

10.3.1.4. Age 40

10.3.1.5. Gender 40

10.3.1.6. Previous stays 40

10.3.1.7. Propensity score 40

10.3.1.8. Individual access route 40

10.3.2. Center-specific parameters 42

10.3.2.1. Team organization (hybrid vs. autonomous) 42

10.3.2.2. Structure type 42

10.3.2.3. Process type 43

10.3.3. Treatment parameters 43

10.3.3.1. Canceling the index treatment 43

10.3.3.2. Treatment duration of the index treatment in days 44

10.3.3.3. Number of contacts from the treatment team per day 44

10.3.3.4. Duration of treatment per day 44

10.3.3.5. Instability 45

10.3.4. Tertiary variables 45

10.3.4.1. Workplace **Fehler! Textmarke nicht definiert.**

10.3.4.2. Place of residence (urban-rural) 46

10.3.4.3. Relocation (yes/no) 46

10.3.4.4. Age at first full inpatient stay 46

10.3.4.5. Total previous full inpatient stays 47

10.3.4.6. Duration of pre-treatment 47

10.3.5. Auxiliary variables 47

10.3.5.1. Number of readmissions to the study clinic within 12 months of admission to the index treatment 47

10.3.5.2. Readmission outside the study clinic within 12 months of admission to the index treatment 48

10.3.5.3. Contact sufficient 48

10.3.5.4. Contact time 48

10.3.5.5. Diagnostic group (for logit model) 48

10.3.6. Employee variables 49

10.3.6.1. Job satisfaction 49

10.3.6.2. Category scores of the employee survey 49

10.3.6.3. Process evaluation 50

10.3.6.4. Experience of the team processes 50

10.3.6.5. Occupational group 50

10.3.6.6. Professional experience 50

10.3.6.7. Age of employee 51

10.3.6.8. Gender of employee 51

10.3.7. Primary and secondary target criteria 51

10.3.7.1. Resumption **Fehler! Textmarke nicht definiert.**

10.3.7.2. Number of treatment days (after discharge from index treatment) 53

10.3.7.3. Time to full inpatient readmission (after discharge from index treatment) 54

10.3.8. Scores from questionnaires 55

10.3.8.1. Health-related quality of life according to EQ5D-5L 55

10.3.8.2. Psychosocial level of functioning according to HoNOS and PSP 55

10.3.8.3. Professional integration through CSSRI-D 55

10.3.8.4. Recovery orientation according to RAS-G 56

10.3.8.5. Treatment satisfaction according to the ZFP questionnaire 56

10.3.8.6. Burden on relatives according to IEQ-EU 57

10.3.8.7. Perceived involvement in decisions according to SDM-Q-9 58

10.3.9. Dates 58

10.3.10. Pre-post variables 58

11. Statistical evaluation methods 59

11.1. Demographic data and other baseline characteristics 59

11.2. Statistics for quantitative outcome research (Module A) 60

11.2.1. Primary outcome target criterion 60

11.2.1.1. Primary analysis (deductive) 60

11.2.1.2. Sensitivity analyses (exploratory) 61

11.2.1.3. Further analyses of the primary criterion 62

11.2.2. Secondary outcome target criteria (exploratory) 62

11.2.2.1. Combined readmission rate 63

11.2.2.2. Extended readmission rate 63

11.2.2.3. Time until full inpatient readmission 63

11.2.2.4. Number of days spent as an inpatient 63

11.2.2.5. Number of inpatient + full inpatient days spent 63

11.2.2.6. Canceling the index treatment 64

11.2.2.7. Health-related quality of life 64

11.2.2.8. Psychosocial level of functioning 64

11.2.2.9. Professional integration 64

11.2.2.10. Recovery orientation 65

11.2.2.11. Perceived involvement in decisions 65

11.2.2.12. Treatment satisfaction 65

11.2.2.13. Sensitivity analyses for the secondary target criteria 65

11.2.3. Tertiary outcome target criteria (descriptive) 67

11.3. Statistics on process and implementation research (Module C) 68

11.3.1. Patient analyses in Module C (exploratory) 68

11.3.1.1. Relationship between the organizational structure and treatment 68

11.3.1.2. Influence of employee job satisfaction on the satisfaction of patients and their relatives 70

11.3.1.3. Correlation between the type of treatment and the burden on relatives 71

11.3.1.4. Influence of a hybrid/autonomous team on the readmission rate and number of days with full inpatient treatment 72

11.3.1.5. Influence of individual treatment and patient variables on the readmission rate and number of days with full inpatient treatment 72

11.3.1.6. Influence of the instability of a course on the readmission rate and the number of days with full inpatient treatment 75

11.3.2. Evaluation of the employee survey (exploratory) in Module C 76

11.3.2.1. Descriptive analysis of job satisfaction in the centers 76

11.3.2.2. Influence of occupational group affiliation on job satisfaction 76

11.3.2.3. Differences in implementation satisfaction at the study centers 77

11.3.2.4. Correlation of center-specific satisfaction with the introduction of IEHT and job satisfaction. 77

11.3.2.5. Correlation between years in the profession and job satisfaction 77

11.3.2.6. Relationship between age and job satisfaction 77

11.3.2.7. Relationship between gender and job satisfaction 78

11.3.2.8. Descriptive analysis of the ZUF-1 score 78

11.3.2.9. Influence of the structure type on job satisfaction 78

11.3.2.10. Influence of a hybrid/autonomous team on job satisfaction 78

11.3.2.11. Influence of the structure type on the experience of team processes 78
11.3.2.12. Influence of a hybrid/autonomous team on process evaluation 79
11.3.2.13. Descriptive analysis of satisfaction in the structural types 79
11.3.2.14. Descriptive analysis of the employee survey at item level 79
11.3.2.15. Descriptive analysis of demographic data 79
11.3.3. Sensitivity analyses (exploratory) in module C 79
11.3.3.1. IEHTPP collective 79
11.3.3.2. Full case analysis 80
11.3.3.3. Correlation of the independent variables 80
11.3.3.4. Correlation of the target variables 80
11.3.3.5. Extended readmission rate and number of inpatient days (in the study hospital) after discharge from the index treatment 80
11.3.4. Testing validity and reliability 81
11.4. Safety variables 81
11.5. Multicenter data 81
11.6. Dealing with multiple comparisons 81
11.7. Interim analyses 81
11.8. Stratification 82
11.9. Subgroup analyses 82
12. Bibliography 83
13. Attachments **Fehler! Textmarke nicht definiert.**
13.1. Structure and listing of the tables 84
13.2. Items of the employee survey 94
13.3. List of clinics 100
13.4. Table 2 from Ludwig et al. 101
13.5. Seeds for imputations 102

2. Introduction

2.1. Background

Mental illnesses have increasing incidence and prevalence rates and have complex, often chronically recurring courses. The inpatient stays associated with prolonged absences from home often lead to restrictions in the social participation of those affected and to high costs for society as a whole (Wittchen et al. 2011). The traditional division between outpatient, day-clinic and inpatient care often reaches its limits, particularly when it comes to the needs-based treatment of people with severe mental illnesses, as many people do not want to be treated as inpatients even in acute crises. For example, treatment outside the clinic is associated with less stigmatization. However, the treatment of such crises by general practitioners alone usually exceeds their capacities, so that the decision to admit patients to hospital often has to be made due to a lack of intensive outpatient alternatives. Therefore there have long been calls for stronger networking, overcoming cross-sector boundaries, more outreach forms of treatment and greater flexibility in care (Bühring, 2017; Roick et al., 2005). Against this backdrop, the Act on the Further Development of Care and Remuneration for Psychiatric and Psychosomatic Services (PsychVVG) introduced inpatient-equivalent psychiatric treatment as a new hospital service for mentally ill patients in 2018 (§ 115d Social Code Book V). This is defined as hospital treatment in the patient's home environment, provided by mobile, medically supervised, multi-professional treatment teams (GKV, 2017). This new form of treatment is classified as home treatment (HT) and corresponds to full inpatient treatment in terms of content, flexibility and complexity. The legislator is thus fulfilling the requirements of the S3 guideline "Psychosocial therapies for severe mental illness", which recommends outreach care approaches during acute crises on the basis of comprehensive evidence assessment (DGPPN, 2019). According to this guideline, the effects of HT have been proven, particularly with regard to a reduction in the inpatient readmission rate, an improvement in treatment satisfaction and a reduction in treatment discontinuation compared to routine treatment (DGPPN 2019). In its report on reducing inappropriate care and improving needs-based management in psychiatry, the German Council of Experts for the Assessment of Developments in Health Care also recommends the development of new, intensive outpatient and multimodal services in the outpatient sector and points out that this expansion should be linked to the development and results of the evaluation of inpatient-equivalent treatment (SVG, 2018).

IEHT is offered at 25 clinics across Germany (as at 8/2019). More were added in 2020 and a large number of the 400 or so specialist hospitals and psychiatric departments at general hospitals and university hospitals are planning to introduce this treatment module in the coming years. According to a wide range of expert opinions (SVR 2018) and initial patient-based surveys (Längle, Holzke and Gottlob, 2019, see section 4.3), in the medium term, 10-15% of all inpatient treatments can be carried out as inpatient-equivalent treatment in the home of the person being treated. According to various data on hospital statistics from 2017, this corresponds to around 100-150,000 patients in Germany per year (see Statistisches Bundesamt, 2019). Scientifically validated findings on the effectiveness and best possible implementation of this form of treatment are therefore of enormous practical relevance.

2.2. Study objective

The overarching aim of the proposed study is to investigate the implementation, treatment processes, clinical effectiveness and costs inpatient equivalent home treatment (IEHT) according to § 115d Social Code Book V in comparison to conventional inpatient treatment from the perspective of patients, relatives and practitioners. Using qualitative and quantitative methods of health services research as well as complementary data sources, a robust, widely accepted evidence base for the further development of outreach, team-based forms of psychiatric treatment in the home environment as an alternative to inpatient care is to be created with the involvement of relevant interest groups and perspectives. The project objective is subdivided into sub-objectives, which are worked on in individual modules (A-D). Methods of outcome, process and implementation research as well as health economic evaluation are applied in the modules (see Illustration 1).

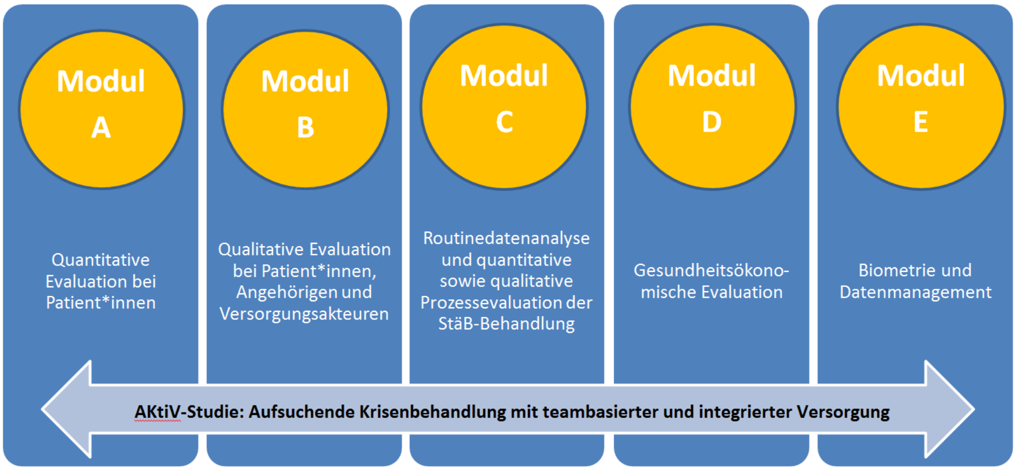

Illustration 1Breakdown of the individual project modules

This SAP comprises the statistical evaluation in Module E, which relates to the research questions of Module A and Module C. Module B is responsible for qualitative research and Module D for health economics research, each with its own competencies.

Primary research question (outcome research): Are there differences in inpatient psychiatric readmission rates over the 12-month period between patients receiving inpatient or inpatient-equivalent treatment? The **primary working hypothesis** is that patients treated by means of IEHT have a significantly lower inpatient psychiatric readmission rate 12 months after inclusion in the study than patients who received conventional inpatient psychiatric treatment during the study period. Although the readmission rate is an imperfect indicator of treatment quality in psychiatry (Durbin et al. 2007), it can certainly be considered an indication of successful acute treatment, recovery and meeting needs in the community psychiatric setting (Byrne et al. 2010; Olfson et al. 1999). In addition, this outcome parameter was used in most international studies on home treatment (DGPPN, 2019). In the present study, the full inpatient psychiatric readmission rate is supplemented by the combined readmission rate (full inpatient/partial inpatient/IEHT) and other outcome parameters. The following secondary research questions should also be answered in this study: Do the readmission rate (full inpatient + partial inpatient + IEHT), the total number of days spent in full inpatient psychiatric care and treatment discontinuations decrease and do health-related quality of life, psychosocial functioning level, occupational integration, treatment satisfaction, perceived involvement in treatment decisions and recovery orientation increase in inpatients receiving inpatient equivalent treatment compared to patients receiving full inpatient psychiatric care? In addition, relatives of study patients will be asked about their treatment satisfaction and their experience of stress in the context of the respective form of care. The qualitative outcomes

examined in this research project are the subjective experience of those affected, including relatives, and the benefits they perceive from IEHT. As part of a **health economic evaluation**, direct and indirect costs of the two forms of treatment will be compared and a cost-benefit analysis carried out. **Process research** will analyze which target group benefits most from IEHT, which impact factors are associated with successful treatment processes, and whether and when admission should take place directly or from inpatient treatment. Due to the limited experience with outpatient treatment in Germany, the question of the target group for this form of home treatment in terms of diagnosis, severity of illness and psychosocial situation has not yet been clarified. The development, organization and satisfaction of the treatment teams, their impact on the target criterion and the impact on the regional care system (system effects) will be examined as part of the **implementation research**.

2.3. References to the curriculum

The statistical analysis plan refers to the study plan (see ethics application) according to the application dated October 19, 2020. The statistical analysis plan (SAP) was prepared on the basis of the Standard Operating Procedure (SOP) ST03 (SAP) of the Competence Center for Clinical Studies Bremen (KKSB). The statistical analysis described therein is based on the requirements of the ICH Guidelines and the SOPs ST01 (collectives), ST04 (missing data) and ST05 (general statistics).

2.4. Amendments

No changes have been made to the curriculum since the final version when the SAP was created.

3. Study management

3.1. Applicant/consortium management

Applicant: Prof. Dr. Med. Sebastian v. Peter
Brandenburg Medical School

Immanuel Clinic Rüdersdorf
Seebad 82/83
15562 Rüdersdorf near Berlin

3.2. Scientific project management

Scientific project management: Prof. Dr. Andreas Bechdolf
Vivantes Hospital Am Urban
Clinic for Psychiatry, Psychotherapy and Psychosomatics
Dieffenbachstrasse 1
10967 Berlin-Kreuzberg

Dr. phil. Johanna Baumgardt
Vivantes Hospital Am Urban
Clinic for Psychiatry, Psychotherapy and Psychosomatics
Dieffenbachstrasse 1
10967 Berlin-Kreuzberg

Dr. Dr. Stefan Weinmann
Vivantes Hospital Am Urban
Clinic for Psychiatry, Psychotherapy and Psychosomatics
Dieffenbachstrasse 1
10967 Berlin-Kreuzberg

M. Sc. Psych. Konstantinos Nikolaidis
Vivantes Hospital Am Urban
Clinic for Psychiatry, Psychotherapy and Psychosomatics
Dieffenbachstrasse 1
10967 Berlin-Kreuzberg

3.3. Biometrics

Responsible biometrician: Prof. Dr. Dr. h.c. Jürgen Timm
Competence Center for Clinical Studies Bremen
University of Bremen
Linzer Str. 4
28359 Bremen

4. Signatures

Prof. Dr. Andreas Bechdolf	______________________	__________
Scientific project management<br>Vivantes Hospital Am Urban<br>Clinic for Psychiatry, Psychotherapy and Psychosomatics	Signature	Date
Dr. phil. Johanna Baumgardt	______________________	__________
Scientific project management<br>Vivantes Hospital Am Urban<br>Clinic for Psychiatry, Psychotherapy and Psychosomatics	Signature	Date
Prof. Dr. Dr. h.c. Jürgen Timm	______________________	__________
Responsible biometrician<br>Competence Center for Clinical Studies Bremen,<br>University of Bremen	Signature	Date

5. List of abbreviations

Abbreviation	Meaning
CWL	Case-Wise-Listing
FAS	Full analysis set
GCP	Good Clinical Practice
ICH	International Council for Harmonization
IG	Intervention group
ITT	Intention-To-Treat
CG	Control group
KKSB	Competence Center for Clinical Studies Bremen
Max	Maximum
Mean	Mean value
Min	Minimum
Emp-C	Employee collective
N	Number of evaluable values
N miss	Number of missing values
PctN	Percentage N
PP-	Per-protocol collective
Q1	First quartile
Q3	Third quartile
SAP	Statistical analysis plan
S-C	Safety collective
SOP	Standard Operating Procedure
IEHT	Inpatient equivalent home treatment
IEHT-C	IEHT collective

Abbreviation	Meaning
IEHT-PP-C	IEHT-per-protocol collective
Std Dev	Standard deviation
SAE	Serious adverse event

6. General specifications

6.1. Hardware and software used

The statistical analysis is carried out with the program package SAS® Version 9.4 (TS1M3) and SYSTAT Version 13. The statistical report is created using Microsoft Office Word 2019. KKSB computer equipment is used for the evaluation. The evaluation is carried out in a separate subnet of the KKSB (for a more detailed description, see the study's data protection concept).

6.2. Reporting standards and reporting language

The statistical report is written in German. It is based on the standard operating procedure (SOP) of the KKSB and is in accordance with the ICH E3 guideline (structure and content of clinical study reports). The statistical report should be prepared in the following structure:

1st title page
2. synopsis
3. table of contents
4 List of abbreviations and explanations of terms
5. ethics
6 Investigators and management structure of the study
7. Introduction
8. study objectives
9. test plan
10. study population
11 Evaluation of effectiveness
12. evaluation of security
13 Discussion and general conclusion
14. tables, charts and lists that are cited but not listed in the text
15. bibliography
16. appendix.

6.3. General format of table, figure and list content

The tables, figures and the listing of patient data are created in SAS® 9.4 and converted into Rich Text Format (RTF). A more detailed description of the content and layout of the presentation of results can be found in the appendix under 13.1.

6.4. Quality control

The clinical study is conducted in accordance with the Standard Operating Procedures (SOPs) for clinical studies of the KKSB and, where applicable, the trial centers as well as the ICH Guideline on Good Clinical Practice (ICH, GCP Good Clinical Practice). For internal quality control, the central statements of the evaluation are checked by a second biometrician.

The data management of this study follows the SOPs of the KKSB. The quality control of the incoming data is carried out via plausibility checks and queries (SOP DM05). Further details can be found in the data management plan.

The statistical analysis is carried out using the statistical analysis plan (SAP). Any deviation from the SAP is listed in the statistical report with reasons. A quality check is carried out to verify the consistency of the analysis and the SAP. In addition, the programs are validated in accordance with the KKSB Standard Operations Procedure (SOP ST08). The primary evaluation is checked independently by a second biometrician using different statistical software (R).

7. Details of the study design

7.1. General information on the design

This project is not an efficacy study, but a multicenter proof-of-concept study. In this study, the above-mentioned form of treatment is examined with regard to clinical effects as well as subjective and care-related aspects. The study is divided into modules, each of which investigates different topics and questions using a mixed-methods design.

The study participants will be recruited in ten study centers over a period of 12 months. Until the planned number of cases is reached, all IEHT patients will be asked to participate after being informed about the study. If consent is given and the inclusion criteria are met, the patients are included in the study. Each included person is then matched with a patient according to the propensity score. If the matched person meets the inclusion criteria, they are also informed in the same clinic and, if they agree, recruited as a control patient. After inclusion, a prospective survey of all patients is conducted at four points in time: Baseline (up to 7 days after admission and at the end of the index treatment), 6-month follow-up (6 months after admission), 12-month follow-up (12 months after admission). Admission to the index treatment is set as the time of admission. The survey data is supplemented by information provided by the attending physician or by entries made by the treatment team in the hospital information system and routine data from the individual clinics in accordance with §301 Social Code Book V (e.g. for health economic analyses).

In addition, the next of kin in the sense of a "person permanently living in the same household" of each study participant is asked about participation in the study. In the absence of close relatives, a close caregiver, such as a live-in caregiver, could also be approached regarding participation in the study.

The employees in the IEHT teams at the local study centers are surveyed using questionnaires on satisfaction, workload and team processes.

In addition to the quantitative survey of patients, relatives and staff, qualitative focus group and expert interviews will be conducted with a sample of IEHT patients and their relatives as part of a multi-modal, participatory research approach. A questionnaire survey of selected IEHT team members, experts and focus group participants is also planned.

7.1.1. Study population and selection of study participants

Inclusion criteria:

1. Relevant psychiatric crisis with existing psychiatric diagnosis group F0X, F1X, F2X, F3X, F4X, F5X or F6X
2. 18 years or older
3. No acute danger to self or others that would require hospitalization
4. Permanent residence in the service area of the respective clinic
5. (Potential) suitability of the social and/or residential environment
6. (Potential) lack of risk to the child's welfare
7. (Potential) consent of all adults living in the patient's home
8. Voluntariness

Exclusion criteria:

1. Placement order according to the respective state laws
2. Severe organic brain diseases with cognitive deficits
3. Intellectual disability
4. Lack of capacity to consent at the time of study inclusion
5. Participation in another intervention study at the time of study inclusion and during the index stay
6. Insufficient language skills for an interview in German

7.1.2. Termination criteria

In the AKtiV study, only the active wish of the participants to withdraw their consent to participate in the study verbally or in writing is envisaged as a criterion for study discontinuation. If the participant withdraws their consent (verbally or in writing), then we speak of a drop-out. In this case, the further collection of data ends. The drop-out and date are documented in the CRF. If the participant also withdraws his/her consent to the processing of the previously collected data, the data will be deleted in accordance with the data protection concept.

7.1.3. Intervention

Intervention in the intervention group (IG) takes place within the framework of the service description according to § 115d Social Code Book V, the federal framework agreement of the self-governing partners and the regulations laid down in the OPS: Patients are admitted via various channels (telephone contact, referral by doctors or community psychiatric providers, social psychiatric services, crisis services, wards, rescue centers, etc.). After checking the IEHT inclusion criteria (see 7.1.1), a needs assessment is carried out by the multi-professional IEHT team. Based on this, a treatment plan is drawn up, which is also based on discussions with patients, relatives, carers and/or other people in the social environment and takes into account the triggers of the current crisis, previous history, previous treatments, current needs and personal preferences. The treatment plan contains treatment goals to be achieved by the patient with the support of the IEHT team, as well as the support provided (medication, conversations, contact with other people, activities, etc.). In addition to the treatment plan, a therapy and medication plan is drawn up for each week. The patient continues to live in his/her social environment and receives at least one personal contact every day in the form of a home visit or a visit to the clinic. The discussions/treatment measures take place in the patient's home, in the clinic or in another place where the patient feels comfortable.

The intervention in the control group (CG) consists of full inpatient treatment within the framework of the legal requirements.

7.1.4. Consideration of possible full inpatient pre-treatment time in the definition of index treatment

Overall, the IG and CG can each be divided into two groups according to the recording mode (see 10.3.1.2) in the treatment, resulting in the following 4 groups:

- Direct admissions to the IEHT (IG)
- Transfer from full inpatient to IEHT (IG+)
- Full inpatient control subjects for IEHT direct admissions (CG)
- Full inpatient control subjects for patients who were transferred from full inpatient to IEHT (CG+)

In all cases, the discharge day from the index treatment is set as the discharge day from the IEHT or full inpatient treatment (item A3_03 in the eCRF). For IG and CG, the day of admission to the index treatment is defined as the day of admission to IEHT or full inpatient treatment (item A3_02). For IG+ and CG+ patients, the day of admission to the index treatment is

documented in the monitoring table and entered in the eCRF (item A504_01). For CG+ patients, the day of admission is defined as the day from which the patient can be addressed.

7.1.5. Planned treatment regimen and duration of treatment

Once a week, a specialist visit takes place in which the achievement of treatment goals to date is assessed, therapies including medication and their dosages are reflected upon and the further duration and type of treatment are determined. Each patient is discussed in detail at least once a week in a team meeting. Pharmacological, psychotherapeutic and psychosocial care is provided in accordance with the standards of the respective clinics. Discharge management is carried out in the same way as inpatient stays. Further specifications regarding team composition, professions involved, processes, etc. are not made for the study centers in order to be able to examine the variance in the implementation of IEHT.

7.1.6. Number of cases

The case number calculation for the primary study endpoint (readmission rate 12 months after study inclusion) is based on international studies included in the current Cochrane Review *Crisis intervention for people with severe mental* illnesses (Murphy et al. 2012) as well as the analysis of IEHT routine data from the participating study centers. In a first step, the mean readmission rate from the publications by Hoult et al. (1983), Fenton et al. (1998) and Johnson et al. (2005) was estimated to be around 72% (CG) to 42% (IG), weighted according to the respective numbers of participants in the studies (sample sizes). To safeguard against possible problems when transferring these figures, the somewhat more pessimistic assumption of 45% for the IG and 70% for the CG was used. This assumption is in line with the current S3 guideline "Psychosocial therapies for severe mental illness", which postulates a difference in the full inpatient readmission rate of 25% in the 12-month period between IG and CG (DGPPN, 2019). This means that a ratio of approximately one third (exactly 0.3571) is expected between the readmission rate of IG and CG.

In a second step, data was collected and analyzed from nine of the study centers participating in the AKtiV study with a total of N=37,007 patients who were treated as inpatients over a period of twelve months. The analysis showed that the readmission rate for patients treated as inpatients was around 52.1 %, which is around 20 % lower than previously assumed.

The analysis in a small retrospective pilot study (Weinmann et al.) with N=86 comparing the readmission rate of IEHT patients and matched inpatients treated at the Berlin Vivantes Hospital Am Urban study center showed an even smaller difference between the readmission rate of IEHT patients (37.2%) and the readmission rate of inpatients (46.5%) at the 12-month follow-up (i.e. a reduction of the ratio by about one fifth). According to this data, a readmission rate ratio between IG and CG of one third (as initially expected) seems too optimistic. Therefore, a new and more realistic approach, taking into account the data described above, was needed. A readmission rate of 52.1% in the CG and a 0.282 lower readmission rate in the IG (weighted mean with a 10% higher weight for the ratio found in the literature) result in an expected readmission rate after IEHT of 37.4%.

With an α error of 5% and a power of 80%, a total of 360 patients in the overall cohort (IG=180, CG=180) would be required in the two-sided test (chi-square test) according to the case number calculation with the nQuery Advisor 7.0 program in order to be able to show such a difference. If the non-responder rate is estimated at 10%, a total of approx. 400 patients would have to be approached regarding participation in the study. Assuming an average duration of inpatient-equivalent treatment of 23.5 days (range=19-37) and an average number of 6.5 IEHT places (range: 6-7) in each of the ten participating study centers, this means that a maximum of 84 IEHT patients could be recruited per year in each study center or 847 for the study as a whole. Recruiting the same number of control patients is unproblematic given the capacities of the ten centers, meaning that a potential 1695 patients could be recruited for the study. This ensures that the required number of 400 patients can be recruited.

It is also planned to interview 400 relatives (200 relatives of patients in IG and 200 relatives of patients in CG).

In addition, 100-150 IEHT employees (approx. 10-15 IEHT employees per study center) are to be surveyed.

7.2. Evaluation criteria

This chapter lists the variables used in the analysis. A description of the calculation of the variables can be found in the chapter 10.3. on data transformation.

7.2.1. Demographic data and other baseline parameters

According to the study application, the following demographic and diagnostic parameters of the patients are collected upon inclusion in the study:

1. Treatment group
2. Recording mode
3. Diagnostic group (FX)
4. Age
5. Age group
6. Gender
7. Pre-stay
8. Propensity score
9. Individual access route
10. Health-related quality of life according to EQ5D-5L
11. Psychosocial functioning level according to HoNOS and PSP
12. Professional integration through CSSRI-D
13. Recovery orientation according to RAS-G
14. Income
15. Marital status
16. Highest school-leaving qualification
17. Highest vocational training
18. Mother tongue
19. Previous full inpatient treatment
20. Time since the last full inpatient treatment
21. Housing situation
22. Employment

The variable Individual access route is only collected for IEHT patients.

These other center-specific parameters are also recorded:

1. Team organization (hybrid vs. autonomous)
2. Structure type
    a) Organization of the department
    b) Team size
    c) Type of clinic

    d) Supply region
3. Process type

The following baseline parameters are collected from the patients' relatives:

1. Baseline burden on relatives according to IEQ-EU

7.2.2. Treatment parameters

The baseline parameters are supplemented by the following patient-specific treatment parameters, which relate to the index treatment period.

a) for all patients:

1. Cancelation of the index treatment
    a. Reasons for discontinuing treatment
2. Treatment duration of the index treatment in days

b) for IEHT patients:

1. Number of contacts from the treatment team per day
2. Treatment duration in minutes per day across all occupational groups
3. Treatment duration in minutes per day per occupational group
4. More than just a relocation
5. Interruption of treatment
6. Serious complications
7. External aggression
8. Substance abuse
9. Tracing message
10. Stability details (score)
11. Stability of treatment (score)

7.2.3. Tertiary variables

1. Diagnosis (FXY)
2. Place of residence (urban-rural)
3. Employment situation
4. Relocation (yes/no)

5. Age at first full inpatient stay
6. Total full inpatient stays
7. Duration of pre-treatment

The auxiliary variables described in the following chapter are only used as support for various analyses (e.g., for imputation).

7.2.4. Auxiliary variables

1. Number of full inpatient readmissions to the study clinic within 12 months of admission to the index treatment
2. Number of extended readmissions (full inpatient + IEHT) in the study clinic within 12 months of admission to the index treatment
3. Number of combined readmissions (full inpatient + partial inpatient + IEHT) in the study clinic within 12 months of admission to the index treatment
4. Full inpatient readmission outside the study clinic within 12 months of admission to the index treatment
5. Extended readmission outside the study clinic within 12 months of admission to the index treatment
6. Combined readmission outside the study clinic within 12 months of admission to the index treatment
7. Sufficient contact before treatment (relatives)
8. Sufficient contact during treatment (relatives)
9. Contact time before treatment (relatives)
10. Contact time during treatment (relatives)
11. Diagnostic group (for logit model)

7.2.5. Employee variables

1. Job satisfaction
2. Category scores of the employee survey
3. Experiencing the team processes
4. Process evaluation
5. Professional group
6. Professional experience

7. Professional experience in outreach services
8. Professional experience IEHT
9. Age of employee
10. Gender of employee

7.2.6. Assessment of effectiveness

7.2.6.1. Primary target variable

Full inpatient readmission within 12 months of admission to index treatment

7.2.6.2. Secondary and other target variables

1. Full inpatient readmission within 6 months of admission to index treatment
2. Combined readmission (full inpatient + partial inpatient + IEHT) within 6 and 12 months of admission to index treatment
3. Extended readmission (full inpatient + IEHT) within 6 and 12 months of admission to index treatment
4. Full inpatient readmission within 6 months of discharge from index treatment
5. Time to full inpatient readmission after discharge from index treatment
6. Number of days spent as an inpatient (in the study hospital) after discharge from the index treatment within 6 and 12 months after admission to the index treatment
7. Number of days spent in IEHT + full inpatient care (in the study clinic) after discharge from the index treatment within 6 and 12 months after admission to the index treatment
8. Number of days spent as an inpatient (outside the study hospital) after discharge from the index treatment within 12 months of admission to the index treatment
9. Number of days spent as an inpatient (outside the study clinic) after discharge from the index treatment within 12 months of admission to the index treatment
10. Cancelation of the index treatment
11. Health-related quality of life according to EQ5D-5L at 6 and 12 months after inclusion in the index treatment
12. Psychosocial functioning level according to HoNOS and PSP at 6 and 12 months after admission to index treatment
13. Occupational integration through CSSRI-D after 6 and 12 months following admission to index treatment

14. Recovery orientation according to RAS-G after 6 and 12 months after admission to index treatment
15. Treatment satisfaction immediately after discharge from the index treatment according to the ZFP questionnaire
16. Treatment satisfaction of relatives immediately after discharge from the index treatment according to the ZFP questionnaire
17. Perceived involvement in decisions immediately after discharge from index treatment according to SDM-Q-9
18. Difference in the burden on relatives according to IEQ-EU
19. Number of contacts with treatment team per day
20. Treatment duration of the index treatment in days
21. Treatment duration per day across all occupational groups
22. Treatment duration per day per occupational group
23. Treatment continuity 6 and 12 months after admission to index treatment
24. Direct costs
    are evaluated separately by Module D
25. Indirect costs
    are evaluated separately by Module D

7.2.7. Safety assessment

All serious adverse events (SAEs) are included in the evaluation.

8. Dealing with protocol violations

If the protocol cannot be adhered to, the corresponding protocol violations are documented by the investigator and forwarded to data management. Prior to the statistical analysis, protocol violations are discussed for each individual patient and classified as major or minor. The following violations are considered major:

- Violation of the inclusion and exclusion criteria
- Error in treatment group assignment
- Missing information on the primary endpoint (for the definition of missing values for the primary criterion, see **Fehler! Verweisquelle konnte nicht gefunden werden.**).

9. Evaluation collectives

9.1. Safety collective (SC)

All patients included in the study form the safety collective (SC). All safety analyses are based on the SC. In this study, it corresponds to the FAS (see below) and is therefore only listed here for the sake of completeness.

9.2. Full analysis set (FAS) or intention-to-treat collective

All patients included in the study form the full analysis set (FAS). This definition corresponds to the intention-to-treat (ITT) approach. The statistical evaluation of efficacy uses the FAS.

9.3. Per-protocol collective

The per-protocol collective (PPC) includes all patients for whom no significant protocol deviations have occurred. If there is a significant protocol violation, the patient or patient data is excluded from the PP analysis. For the definition of clinically significant protocol violations, see chapter 8. The PPC is used in the statistical analysis to perform sensitivity analyses.

9.4. IEHT collective

All IEHT cases in the FAS form a subgroup, the IEHT collective (IEHTC). The IEHTC is the basic collective for the analyses according to Module C.

9.5. IEHTPP collective

All IEHT cases in the PPC form a subgroup, the IEHTPP collective (IEHTPPC). The IEHTPPC is used for sensitivity analyses according to Module C.

9.6. Employee collective

All employees of the 10 centers for which quantitative data was collected are combined to form the employee collective (Emp-C). The Emp-C is taken into account in the evaluation in accordance with Module C.

10. Data Handling

10.1. Dealing with missing data, outliers and implausibility

Implausible data is checked by queries. Outlier tests are not carried out.

If a survey time is omitted, the patient is contacted again shortly before the next survey time in an attempt to organize a survey appointment for the upcoming survey time. The missing data will be treated as "missings".

Subjects who cannot be followed up, either because they can no longer be contacted or because they are unable to participate in a particular phase of the study for whatever reason, are first documented as "missings". These subjects are then contacted by telephone if possible and the primary endpoint is queried. At the end of registration, a "lost to follow-up" is documented in the e-CRF with the reason and the last contact date.

Missing data for the target variables required for the analysis are replaced by multiple imputation. The imputation methods used are described in the statistical report and reference is made to the results obtained with imputed data (for a detailed description of multiple imputation, see 10.1.1).

Due to different treatment durations of the index treatment and follow-up appointments that deviate from the plan, the time intervals until the follow-ups can also vary (see 10.1.1.4).

10.1.1. Multiple imputation of missing data

If more than 5% of the data for a variable are missing, this can lead to a substantial bias in the results of the statistical analysis (Jakobsen et al., 2017). In these cases, the mechanism underlying the missingness may influence the results, including those obtained by applying multiple imputation. Therefore, the procedure described in the next paragraph is carried out for the variables to be imputed (see below).

To check whether the missing data is MCAR (missing completely at random), the following procedure is carried out separately for full inpatients and IEHT patients. First, the patients are divided into two groups. The first group contains the patients for whom the value of the variable under consideration is missing. The other group contains the patients for whom the value is not missing. Homogeneity tests are then carried out in the two groups with regard to the

variables age, gender, previous stays and diagnosis group (FX). For this purpose, Mann-Whitney tests for age and previous stays, a chi-square test for gender and a Fisher test for the diagnostic groups (FX) are carried out exploratively with $\alpha = 5\%$. If there is a significant difference, it is assumed that the data is not MCAR and assume MAR (missing at random). This means that the missingness of the data does not depend on the missing variable itself, but may depend on other variables.

It is important to note here that in the case of the MAR assumption, it cannot be ruled out that the missingness is dependent on the missing variable itself, i.e. that the missing data is MNAR (missing not at random). In this case, the requirements of multiple imputation would be violated and this could cause a distortion of the results. This should be pointed out in the statistical report.

Regardless of the mechanism underlying the missingness, all missing values of questionnaire scores (for the individual scores see 10.3.8) and of the variables full inpatient readmission, extended readmission and combined readmission are multiply imputed, if between 5% and 50% of the data are missing for the variable and it is used as the target variable in an analysis. If less than 5% of the data is missing, a full case analysis is performed; if more than 50% of the data is missing, the planned analysis is not performed except for the descriptive statistics. Scores from surveys of relatives are excluded from multiple imputation; only complete data sets are analyzed here (full case analysis).

The following steps are carried out separately for patients in the CG and the IG. A regression model is created for each variable (for the different models, see 10.1.1.1-10.1.1.3).

Regression parameters (including their distribution) and the residual variance are now estimated on the basis of the complete data sets and the following procedure is carried out 100 times.

A set of regression coefficients and a variance (corresponding to the estimated variance, from a chi-squared distribution) are randomly generated from the distributions. The generated regression coefficients are used to calculate a forecast for missing values. A normally distributed random variable with an expected value of 0 and the generated variance is added to this forecast. In SAS, this procedure is implemented with the MI procedure. The seeds to be selected for generating the random numbers can be found in Table 20 in the appendix 13.5. It should be noted that different cases are considered for some variables (see 10.1.1.1 and 10.1.1.3) and therefore several seeds are necessary. Overall, the seeds are also made up of

the chapter number (without the preceding 11) and the position of the analysis within the chapter.

In the case of a logistic regression model, only a probability is generated. To obtain a binary variable, a random value is drawn from a Bernoulli distribution with the corresponding probability parameter.

A total of 100 imputations are obtained for each missing value and 100 complete data sets for each variable. Each data set is analyzed individually and the result parameters are combined using the following formulas (Little & Rubin, 2002).

Let β be the parameter of interest and V its variance. From the analysis performed on the 100 different data sets, we then obtain the estimators $\widehat{\beta_1}, \dots, \widehat{\beta_{100}}$ with the corresponding variance estimates $\widehat{V_1}, \dots, \widehat{V_{100}}$. Then the combined estimator $\widehat{\beta_{\mathrm{MI}}}$ can be calculated by

$$\widehat{\beta_{MI}} = \frac{1}{100} \sum_{k=1}^{100} \widehat{\beta_k}$$

The total variance is estimated with

$$\widehat{V_{MI}} = \left(1 + \frac{1}{100}\right) \hat{B} + \widehat{W}$$

where $\widehat{W}$ describes the estimation of the within-variance

$$\widehat{\mathrm{W}} = \frac{1}{100} \sum_{k=1}^{100} \widehat{V_k}$$

and $\hat{B}$ the estimation of the between-variance

$$\hat{B} = \frac{1}{100 - 1} \sum_{\mathrm{k}=1}^{100} \left(\widehat{\beta_k} - \widehat{\beta_{MI}}\right)^2$$

For a detailed description of the pooling of the individual values in the case of an analysis using the Mann-Whitney test, see 10.1.1.4.

10.1.1.1. Multiple imputation of questionnaires at the 12-month follow-up appointment

For each questionnaire collected at the 12-month follow-up appointment, the 12-month values are imputed as follows. A regression model is created for each of the following four different types of missing values:

1. The baseline, 6-month and 12-month values of the variable under consideration are missing.
2. The baseline value is available, but the 6-month and 12-month values of the variable under consideration are missing.
3. The 6-month value is available, but the baseline and 12-month values of the variable under consideration are missing.
4. The baseline and 6-month values are available, but the 12-month value of the variable under consideration is missing.

In the regression models, the variables age, previous stays, diagnostic group (FX) and gender as well as (if available) the baseline and 6-month value are then taken into account as covariates.

The variables described in this section include the corresponding scores of the EQ5D-5L, HoNOS, PSP, RAS-G and CSSRI-D questionnaires (see 10.3.8).

The values for occupational integration according to CSSRI-D are imputed using logistic regression instead of linear regression. It should be noted that only the 12-month value of the occupational integration score (see 10.3.8.3) is imputed and the variable diagnosis group (for logit model) is used as a covariate instead of diagnosis group (FX). The score for the change in occupational integration is then calculated using the imputed 12-month value and the baseline value. If the baseline value is also missing, a 0 is set here for the score.

10.1.1.2. Multiple imputation of questionnaires at the end of the index treatment

A linear regression model with the covariates age, previous stays, diagnostic group (FX) and gender is created for each of the variables.

The variables to be imputed include treatment satisfaction and perceived involvement in decisions (see 10.3.8).

10.1.1.3. Multiple imputation of the resumption

This chapter describes the multiple imputation for the variables full inpatient readmission, extended readmission and combined readmission.

As can be seen from the data transformation (**Fehler! Verweisquelle konnte nicht gefunden werden.**), imputation is required in two different cases. Firstly, if the values from the 6- and 12-month follow-up are missing. Secondly, if the patient was not admitted after 6 months and the value of the 12-month follow-up is missing (in both cases, imputation is only necessary if no readmission was registered in the HIS).

Due to the use of HIS information, all readmissions in the study hospital are documented. Possibly missing is the information whether a readmission took place outside the study hospital. This variable is imputed and set as target variable y in the following regression models (for the calculation of the variable see 10.3.5.2). The imputed value of the variable "readmission in another clinic" is then used for the missing value of the variable "readmission".

In the first of the cases described above, all patients with an available 12-month follow-up value are used and the following regression model is estimated (abbreviated notation):

y= constant + number of readmissions in the study clinic + age

+ previous stays + gender + diagnosis group (for logit model)

In the second case, all patients are used who have not yet been readmitted to another clinic after 6 months and for whom the 12-month follow-up value is also available. The following model is then estimated (abbreviated notation):

y= constant + number of readmissions in the study clinic + age

+ previous stays + gender + diagnosis group (for logit model)

If a perfect prediction is made, a data expansion method is used. This is described in Chapter 5.2 of the work by White, Daniel & Royston (2010). In SAS, this method is carried out using the suboption LIKELIHOOD=AUGMENT.

In addition, the population is examined for heterogeneity with regard to the variable number of readmissions to the study clinic. For this purpose, the relationship to the target variable is analyzed graphically.

10.1.1.4. Combination of data sets analyzed using the Mann-Whitney test

Many of the scores from the analyzed questionnaires are not normally distributed, which is why the Mann-Whitney test is used as a non-parametric test for the analysis. As carrying out a Mann-Whitney test in combination with multiple imputation is not entirely trivial, the procedure is described and explained again in this section.

First, 100 complete data sets are created using the procedure described above. Then the required Mann-Whitney test is applied to each of the data sets. The resulting Mann-Whitney test statistics are calculated using Rubin's formulas (see 10.1.1). As estimators for the variances $V_k, k = 1, \dots, 100$ of the individual test statistics are the approximate variances resulting from the asymptotic normal distribution (under the null hypothesis) of the Mann-Whitney test statistics (see John & Onu 2018).

This gives us the estimator $\widehat{\beta_{MI}}$ with associated variance $\widehat{V_{MI}}$. Now we can again take advantage of the fact that the Mann-Whitney test statistic is asymptotically normally distributed under the null hypothesis in order to calculate a p-value using this representation. For a justification of the procedure, see Mehrotra & Mogg (2007).

In SAS, the individual results are pooled via PROC MIANALYSE.

10.1.2. Variation of the time intervals from the end of the index treatment to the follow ups

A different length of the time interval from the end of the index treatment to the 12-month follow-up can be caused by different treatment durations of the index treatment (see also 7.1.4) and follow-up dates that deviate from the plan. To take this into account, the duration of the index treatment is tabulated with the number of evaluable values (N), number of missing values (N miss), mean value (Mean), standard deviation (Std Dev), minimum (Min), first quartile (Q1), median, third quartile (Q3) and maximum (Max) according to the treatment groups, taking into account the different admission modes (see Table 6 in the appendix 13.1). In addition, questionnaire data (see 10.3.8) of the 12-month follow-up are only accepted if the 12-month follow-up appointment took place at least 14 days before and at most 14 days after the precisely calculated time (365 days after inclusion in the index treatment). If the 12-month follow-up is not within this interval, the value in question is considered missing. For questionnaire data (see 10.3.8), which is to be collected immediately after discharge from the index treatment, this limit is 7 days after the discharge date.

Excluded from this are variables that relate to hospital stays (**Fehler! Verweisquelle konnte nicht gefunden werden.**-10.3.7.3). The decisive factor here is whether the days spent in a clinic are within the 12 months after admission to the index treatment and not when this data was recorded.

10.2. Dealing with index handling aborts and drop-outs

If the index treatment is aborted or dropped out, the reasons are documented and evaluated. The resulting missing data is replaced using the imputation methods in accordance with section 10.1. The respective patient remains assigned to the ITT or FAS. A list of patients with drop-out, discontinuation of index treatment and protocol violations is created in the appendix of the statistical report in accordance with the ICH-E3 guideline.

10.3. Data transformation

10.3.1. Demographic data and other baseline parameters

This section describes how the baseline variables (7.2.1) are calculated from the individual items. It should be noted that the variables calculated from scores from questionnaires are described in the separate chapter 10.3.8 are described in a separate chapter.

10.3.1.1. Treatment group

The treatment group is recorded via the eCRF item ID_Group. Patients with ID_Group=1 (IG-direct) and ID_Group=2 (IG-from transfer) are assigned to IG (IEHT treatment). Patients with ID_Group=3 (direct inpatient treatment) and ID_Group=4 (inpatient treatment for transfer) are assigned to inpatient treatment.

10.3.1.2. Recording mode

The admission mode is recorded via the ID_Group item and divides the patients into the following four groups: IG (ID_Group=1), IG+ (ID_Group=2), CG (ID_Group=3) and CG+ (ID_Group=4).

10.3.1.3. Diagnosis

The diagnosis (FXY) is recorded with eCRF item PS03. A coarser breakdown is often used in the evaluation by omitting the second digit. This is referred to below as the diagnosis group (FX).

10.3.1.4. Age

The exact age is recorded with eCRF item A3_01 and used in the further evaluation. In addition, the age group is recorded using item PS_age group, which is only used to calculate the PS score. The following coding was used: 1 = up to 19, 2 = 20-29, 3 = 30-39, 4 = 40-49, 5 = 50-59, 6 = 60-69 and 7 = >=70.

10.3.1.5. Gender

Is recorded via eCRF item PS_gender (1=male, 2=female).

10.3.1.6. Pre-stay

Is recorded via eCRF item PS_InpatientStays. The number of previous inpatient stays (IEHT + full inpatient) in the study clinic (in the period 2 years before admission to the index treatment) is specified here.

10.3.1.7. Propensity Score

Is specified in eCRF item PS_Score.

10.3.1.8. Individual access route

The variable individual access route (IEHT transfer or IEHT direct) is recorded by the eCRF item A2_staeb3 (1= IEHT transfer, 2= IEHT direct) and is only determined for hospital patients.

10.3.1.9. Income

The variable income is recorded by the eCRF item css034a (1=salary/wage, 2=pension, 3=social benefits, 4=family support, 5=other).

10.3.1.10. Marital status

The variable marital status is recorded by the eCRF item A1_Marital status (1=single, 2=partnered, 3=married, 4=no response).

10.3.1.11. Highest school-leaving qualification

The variable highest school-leaving qualification is recorded by the eCRF item A1_Graduation (1=unknown, 2=no school-leaving qualification, 3=Volks-/Hauptschule or POS, 4=Mittlere Reife/ Realschule or POS, 5=Fachhochschulreife, 6=Abitur or EOS, 7=other, 8=not specified).

10.3.1.12. Highest professional training

The variable highest vocational training is recorded by the eCRF item A1_Vocational training (1=no training, 2=apprenticeship, 3=vocational college, 4=study, 5=doctorate, 7=other, 8=no answer).

10.3.1.13. Mother tongue

The variable mother tongue is formed as a binary variable (German vs. other). If one of the mother tongues is German (eCRF item A104_01=2), the variable is set to "German". If other is answered (eCRF item A1_Mother_language_other=2) and German is not (eCRF item A104_01=1), then the variable is set to "other".

10.3.1.14. Previous full inpatient treatment

The variable Previous inpatient treatment is recorded by the eCRF item A1_Pre-treatment. A 1 stands for "yes" (patient has previously received inpatient psychiatric treatment) and a 2 for "no".

10.3.1.15. Time since the last full inpatient treatment

The variable Time since last inpatient treatment indicates the difference between eCRF item A3_02 (date of admission) and A1_date_last_stat_treatment in months. It should be noted that the variable is only available if the patient has already been treated as an inpatient (previous inpatient treatment=1, see 10.3.1.14).

10.3.1.16. Housing situation

The housing situation variable is recorded by the eCRF item css002a. The response options are summarized into the following 4 categories:

1. Private (if 1 to 5)
2. Municipality (for information 6 to 9)
3. Hospital (if 10 to 14)
4. Other (for information 15 to 17)

10.3.1.17. Employment

The employment variable is recorded by the eCRF item css010a. The response options are summarized into the following 7 categories:

1. 1. labor market (css010a1, css010a2)
2. 2. labor market (css010a4, css010a11)
3. Unemployment (css010a5)
4. Disability or occupational disability (css010a6)
5. Old-age pension / pension / early retirement (css010a7)
6. Training / retraining (css010a8)
7. Other (css010a12, css010a10, css010a9)

In the eCRF, the coding 1=not selected and 2=selected applies.

10.3.2. Center-specific parameters

The center-specific parameters used in the evaluation are described below.

10.3.2.1. Team organization (hybrid vs. autonomous)

The team organization variable is surveyed using the structural questionnaire. Based on the expert interviews, multiple answers are "corrected" so that each center can be assigned one of the organizational forms ward-integrated, department-bound, hybrid or autonomous. However, the expert interviews showed that only the hybrid and autonomous categories were used by the centers in the study and the variable is therefore binary.

10.3.2.2. Structure type

Based on the results of the qualitative survey, a maximum of 3-4 structural types were to be created using an expert panel in module C1.

However, the preliminary evaluation of the structural questionnaires showed that there is extreme heterogeneity between the study centers, which does not allow them to be grouped into 3-4 structural types. As part of the expert panel, four important criteria were therefore selected, which divide the ten study centers into two groups:

- Organization of the department
    a) Specialist expertise is provided from within the department
    b) Specialist expertise is available in isolation in the IEHT team
- Team size

   a) Total sales > 10
   b) Total sales price < 10
- Type of clinic
   a) University Hospital
   b) Other types of clinics/departments at general hospitals
- Supply region (metropolis with millions vs. the rest)
   a) Million-strong Metropolis
   b) Remaining centers

These four binary variables are used in the evaluation instead of a characterization by a structure type. They are listed in 7.2.1 already listed.

10.3.2.3. Process type

The process type of a center is determined qualitatively by focus groups from Module C.

10.3.3. Treatment parameters

The following section describes the data transformation of the 7.2.2 is described below. The variables from sections 10.3.3.3-10.3.3.5 are only calculated for IEHT patients.

10.3.3.1. Canceling the index treatment

The discontinuation of the index treatment is recorded as a binary variable (yes=1/no=2) using eCRF item A401_07 for IEHT and A501_07 for full inpatients. In addition, the various reasons for discontinuation are recorded as the classified variable reason for discontinuation (eCRF item A402 for IEHT and A503 for full inpatients). The variable reason for discontinuation is recorded with the following values:

1. As a result of serious external events that affect the patient outside the clinic or the clinic setting (e.g. relocation, loss of home, childcare due to the pandemic situation, treatment in a somatic clinic) and prevent the continuation of psychiatric treatment
2. Patient's request against medical advice
3. Disciplinary reasons or non-compliance
4. Death of the patient
5. Not further ascertainable
6. Reasons other than those mentioned above, namely:

Module A is sent a list of the reasons given in point 6. Using expert assessments, an attempt is then made to assign the information to reasons for discontinuation 1-5.

10.3.3.2. Treatment duration of the index treatment in days

When calculating the duration of the index treatment, the different recording modes must be taken into account (see 10.3.1.2). The treatment duration of the index treatment is then calculated as the simple difference between the discharge day from the index treatment and the admission day to the index treatment (for the definitions of the discharge day and admission day of the various admission modes, see section 7.1.4). The discharge day and admission day are each counted as half days.

10.3.3.3. Number of contacts from the treatment team per day

For the number of contacts from the treatment team per day, the number of contacts from all professional groups (eCRF items A604_01 to A607_07) is added together and divided by the number of treatment days (eCRF item A602_01). It should be noted here that two contacts from the same occupational group on one day are recorded as one contact with a total treatment duration.

10.3.3.4. Treatment duration per day

The duration of treatment per day per professional group is calculated in minutes for the professional groups physicians (eCRF items A604_01 to A604_07), psychologists (items A605_01 to A605_07), special therapists (items A606_01 to A606_07) and nursing staff (items A607_01 to A607_07). The codes of the OPS services mean (using the example for the professional group of doctors):

- 9701.00 Up to 30 minutes per day (A604_01)
- 9701.01 More than 30 to 60 minutes per day (A604_02)
- 9701.02 More than 60 to 90 minutes per day (A604_03)
- 9701.03 More than 90 to 120 minutes per day (A604_04)
- 9701.04 More than 120 to 180 minutes per day (A604_05)
- 9701.05 More than 180 to 240 minutes per day (A604_06)
- 9701.06 More than 240 minutes per day (A604_07)

The number of contacts from the corresponding time interval is now entered in the eCRF. In order to calculate the (average) treatment duration per day for one of the occupational groups, the treatment duration in minutes of the respective group is calculated first. To do this, the number of services from the time intervals specified above is multiplied by the mean value of the respective time interval (for services lasting more than 240 minutes per day, 270 is selected as the mean value). The sum of all treatment minutes of an occupational group is then divided by the number of treatment days (eCRF item A602_01).

The treatment duration per day across all occupational groups is calculated as the sum of the treatment durations per day of the individual occupational groups.

10.3.3.5. Instability

The following self-generated scores are used to determine the instability of a treatment course of the index treatment. The validity of these scores is based on the apparent validity from the expert group of Module C.

Unstable courses during the index treatment are characterized by the following dichotomous variables (A401_01-A401_07 in eCRF):

1. More than just a relocation
2. Interruption of treatment
3. Serious complications
4. External aggression
5. Substance abuse
6. Tracing message
7. Canceling the index treatment

In the eCRF, these are coded as 1=occurred and 2=did not occur. A recoding is carried out here so that the following applies for variables 1-6: 1=occurred and 0=did not occur and the following applies for the termination of the index treatment: 6=occurred and 0=did not occur.

The stability details variable is now calculated as the sum of variables 1-6. The stability of the treatment is then used to determine a further score to describe the stability of a treatment course of the index treatment. This is calculated as the sum score of variables 1-7 with a maximum score of 12 when all events occur.

10.3.4. Tertiary variables

To determine the diagnosis variable (FXY), see 10.3.1.3.

10.3.4.1. Employment situation

The employment situation variable is formed from the eCRF item css010a (for the baseline survey) from the CSSRI-D as a binary variable. The following coding applies in the eCRF:

1. Self-employed / Occupation in the open labor market - full-time (css010a1)
2. Self-employed / Occupation in the open labor market - part-time (css010a2)

3. Marginal employment (css010a4)
4. Unemployed / jobseeker (css010a5)
5. Disability or occupational disability (css010a6)
6. Old-age pension / pension / early retirement (css010a7)
7. Training / retraining (css010a8)
8. Housewife / Househusband (css010a9)
9. Voluntarily (unpaid) employed (css010a10)
10. Protected activity (css010a11)
11. Other (css010a12)

In the eCRF, 1 stands for "not selected" and 2 for "selected".

Answer options 4, 5 and 6 form the category "not in employment". All other answer options indicate employment. If at least answer options 1, 2, 3, 7, 8, 9, 10 or 11 indicate that this is the case, the employment situation is coded as 1, otherwise as 0.

10.3.4.2. Place of residence (urban-rural)

The patients' place of residence is determined indirectly by taking into account the location of the clinic (urban and rural study centers). The centers Vivantes Hospital Am Urban, Vivantes Hospital Neukölln, Charité – Berlin University Medicine, Isar-Amper Hospital Munich and Clinic for Psychiatry and Psychosomatics Reutlingen are assigned to the urban category and the remaining centers to the rural category (for a complete list of clinics, see Table 18 in the appendix 13.3). The patient's study clinic is determined by the eCRF variable ID_Study center.

10.3.4.3. Relocation (yes/no)

Item css004 of the eCRF is used to record whether the patient has lived somewhere other than the hospital and the previously specified accommodation in the last 6 months. If this is answered with "yes" (=1 in the eCRF) at the 6- or 12-month follow-up appointment, the patient has moved (coded as 1). If the answer is "no" (=2 in the eCRF) at both appointments or if the question was not answered (e.g. in the event of a survey failure), there is no clear indication of a move (coded as 0).

10.3.4.4. Age at first full inpatient stay

The age at the first full inpatient stay in a psychiatric/psychosomatic clinic is recorded by the eCRF item A1_age_first_treatment. If the patient has not previously received full inpatient

psychiatric treatment (previous full inpatient treatment=2, see 10.3.1.14), then the variable is set to the current age (see 10.3.1.4).

10.3.4.5. Total full inpatient stays

The total number of previous psychiatric inpatient stays is recorded by the eCRF item A1_number_inpatient_stays. If the patient has not previously received full inpatient psychiatric treatment (previous full inpatient treatment=2, see 10.3.1.14), then the variable Total previous full inpatient stays should be set to 0.

10.3.4.6. Duration of pre-treatment

The duration of the pre-treatment is calculated as the difference between the date of inclusion in the index treatment (see section 7.1.4) and item A3_02 of the eCRF. It should be noted that the difference only differs from 0 for CG+ and IG+ patients.

10.3.5. Auxiliary variables

10.3.5.1. Number of readmissions to the study clinic within 12 months of admission to the index treatment

The number of full inpatient readmissions to the study hospital within 12 months of admission to the index treatment is determined by the CSSRI. The eCRF items css040c-css045c, C019_05-C019_28 and C023_03-C023_08 each contain the admission date of a full inpatient psychiatric stay. For the variable described here, all stays in the study clinic are counted for which the admission date is after the discharge date from the index treatment (eCRF item A3_03). To determine whether a stay in the study clinic took place, the entry in the clinic column (eCRF items css040ab-css045ab, C018_05-C018_28 and C022_03-C022_08) is compared with a list of names (compiled in collaboration with the study clinics).

The same procedure is used for the number of extended (full inpatient + IEHT) and combined (full inpatient + partial inpatient + IEHT) readmissions to the study hospital within 12 months of admission to the index treatment. The clinic names of the IEHT stays can be found in the eCRF items css056ab-css059ab and C040_05-C040_16 and the admission dates in css056c-css059c and C041_05-C041_16. The clinic names of the partial inpatient stays can be found in the eCRF items css051ab-css052ab and C035_06-C035_07 and the admission data in css051c-css052c and C036_06-C036_07.

10.3.5.2. Readmission outside the study clinic within 12 months of admission to the index treatment

Full inpatient readmission outside the study clinic within 12 months of admission to the index treatment is recorded in a similar way to the primary criterion (see **Fehler! Verweisquelle konnte nicht gefunden werden.**). The difference is that a stay is only taken into account if the admission did not take place in the study clinic. For this purpose, the entry in the Clinic column (eCRF items css040ab-css045ab, C018_05-C018_28, C022_03-C022_08 and KS1a_0-KS1a_29) is compared with a list of names (created in collaboration with the study clinics). It should also be noted that only admissions to the study clinic are documented in the HIS. This information can therefore not be used here.

The same procedure is used for extended (full inpatient + IEHT) and combined (full inpatient + partial inpatient + IEHT) readmission outside the study hospital within 12 months of admission to the index treatment. The clinic designations of the IEHT stays can be found in the eCRF items css056ab-css059ab, C040_05-C040_16 and KS3a_0-KS3a_15 and for the partial inpatient stays in css051ab-css052ab, C035_06-C035_07 and KS2a_0-KS2a_8.

10.3.5.3. Contact sufficient

If, in the general part of the IEQ questionnaire, question 6 (eCRF: item AB06 for the period before treatment and item AB49 for the period during treatment) indicates that contact (in person or by telephone) with the patient concerned took place for less than one hour per week in the period under consideration (AB06=1 or AB49=1), the entire IEQ-EU may not be evaluated (see 10.3.8.6). For the corresponding period, contact sufficient = 0, otherwise contact sufficient = 1.

10.3.5.4. Contact time

Contact time is recorded by the eCRF item AB06 for the period before treatment and the eCRF item AB49 for during treatment. The answers "1 to 4 hours per week" (=2 in the eCRF) to "more than 32 hours per week" (=6 in the eCRF) are coded 1-5 to form an ordinal score. If "less than one hour per week" (=1 in the eCRF) is given, this variable is counted as missing and is not analyzed.

10.3.5.5. Diagnostic group (for logit model)

The auxiliary variable diagnosis group (for logit model) is initially set analogously to the diagnosis group (FX) from 10.3.1.3 is determined. However, since a logistic regression model

could result in complete separation due to the many different diagnosis groups, patients with the rare diagnosis groups 0 and 5 are instead assigned to a suitable category on the basis of a secondary diagnosis (A311x1-A311x20). If more than one secondary diagnosis was specified, Module A selects the most medically relevant of these.

10.3.6. Employee variables

10.3.6.1. Job satisfaction

Job satisfaction is described by two different scores.

For the first score, items 1-7 from the B11 job satisfaction category of the COPSOQ (German standard version) are used (items B11_1 to B11_7 of the employee survey). The answers are coded with the values 0, 25, 50, 75, 100 from "very dissatisfied" (=5 in the questionnaire) to "very satisfied" (=1 in the questionnaire). The score is then calculated as the average of the individual items. If no information was provided for 4 or more items, the score is not calculated.

The second score (ZUF-1) is calculated using items from the Centrum for Psychiatry Südwürttemberg satisfaction questionnaire. The individual answers are also coded with 0, 25, 50, 75, 100 from "strongly disagree" (=1 in the questionnaire) to "strongly agree" (=5 in the questionnaire). The ZUF-1 is then determined as the mean value of questions Z6:6, Z7:17, Z8:24, Z9:29, Z10:33, Z11:43, Z12:44 and Z13:52 (for the individual questions, see Table 17 in the appendix 13.2). If no information was provided for 5 or more items, the score is not calculated.

10.3.6.2. Category scores of the employee survey

In the following, the category scores of the employee survey are the scores from Table 17 in the appendix 13.2 are referred to below. These are calculated as follows for the COPSOQ and the ZFP questionnaire.

In the COPSOQ, the category scores are the scores of the subscales (see Appendix 13.2 Table 17e.g. Quantitative requirements). Here the answer options are coded from left (=1 in the questionnaire) to right (=5 in the questionnaire) with 0, 25, 50, 75, 100. An exception is item B12, which is coded in steps of 10 (0,10, 20,..., 90, 100). For questions B7:01-B7:04 and B08:01-B08:10 there is an additional answer option 6 ("do not have a supervisor/colleagues"). If this option is selected, the respective item is set to "Missing". If for a subscale in the column

Instrument from Table 17 column, the coding is done from right to left instead. The category score is then calculated as the mean value of the individual items of the subscale.

In the ZFP questionnaire, the category scores are the scores in thick print from Table 17 (e.g. general work situation). Here, too, the individual response options are coded from left to right with 0, 25, 50, 75, 100 (except for items for which the column Interpretation is rescaled, here the coding is from right to left). Sub-scores are then calculated as mean values of the individual items (see thinly printed scores in Table 17 e.g. work content). The category scores are then calculated as the mean value of the sub-scores.

If more than half of the required information is missing when calculating an average value, it will not be calculated.

For each of the calculated category scores, the interpretation column of the Table 17 indicates whether high values are to be interpreted as positive or negative after the described transformations.

10.3.6.3. Process evaluation

The category score (for calculation see 10.3.6.2) of Z14 from Table 17 in the appendix 13.2 is the process evaluation.

10.3.6.4. Experiencing the team processes

The experience of the team processes is calculated as the mean value of the category scores (see 10.3.6.2) of Z6-Z13 from Table 17 in the appendix 13.2 calculated.

10.3.6.5. Professional group

The professional group is recorded using item A4 from the employee survey. The following coding applies: doctors = 1, psychologists = 2, nursing service = 3, social services = 4, occupational therapy/art therapy = 5, music therapy = 6, movement therapy = 7, recovery counselors = 8, other = 9.

10.3.6.6. Professional experience

Professional experience, professional experience of outreach services and professional experience of IEHT are determined by the items Z1 (<1 year = 1, 1-3 years = 2, 3-5 years = 3, 5-7 years = 4, 7-9 years = 5, >9 years = 6), Z2 and Z5 (<1 month = 1, 2-6 months = 2, 7-12 months = 3, 1-2 years = 4, >2 years = 5) from the employee survey.

10.3.6.7. Age of employee

The age of employees is recorded using item A3 (up to 24 years = 1, 25-34 years = 2, 35-44 years = 3, 45-54 years = 4, 55 years and older = 5) from the employee survey.

10.3.6.8. Gender Employee

The gender of employees is recorded using item A2 (Male = 1, Female = 2, Diverse = 3) from the employee survey.

10.3.7. Primary and secondary target criteria

This chapter describes how the functions described in 7.2.6 are calculated. It should be noted that the scores of questionnaires are discussed in the separate chapter 10.3.8. The treatment duration of the index treatment in days, treatment duration per day across all occupational groups, treatment duration per day per occupational group, number of contacts from the treatment team and the discontinuation of the index treatment have already been described in chapter 10.3.3.

10.3.7.1. Readmission

The primary criterion of full inpatient readmission within 12 months of admission to the index treatment is recorded by the CSSRI. The eCRF items css040ab-css045ab, C018_05-C018_28 and C022_03-C022_08 contain the clinic names of the full inpatient psychiatric admissions. If an entry is included here and the corresponding admission date (eCRF items css040c-css045c, C019_05-C019_28 and C023_03-C023_08) is after the discharge date from the index treatment (eCRF item A3_03), the variable is set to 1 for the respective follow-up date. If no admission date is specified for the stay, the variable is also set to 1 (should only be the case if the stay was requested by telephone). Only stays from the areas "Psychiatric or psychotherapeutic ward or clinic" and "Psychosomatic ward or clinic" are taken into account. If values are missing in the CSSRI, the criterion is determined using the following scheme:

Table 1Scheme for determining full inpatient readmission in the event of missing values in the CSSRI

6 months	12 months	HIS	Full inpatient readmission
missing	0	NN	0
missing	1	NN	1
0	missing	0	missing

0	missing	1	1
1	missing	NN	1
missing	missing	0	missing
missing	missing	1	1

A 0 stands for no full inpatient readmission, a 1 for at least one full inpatient readmission and NN for not necessary. The 6 (12) month column indicates the CSSRI value from the 6 (12) month follow-up. It should be noted that the CSSRI summarizes the patient's information, additional data from the HIS and information obtained by telephone because the patient did not show up for the follow-up appointment. The CSSRI always refers to the period since the last survey. If the 6-month follow-up is missing, the patient is also interviewed about the first 6 months after admission to the index treatment at the 12-month follow-up. If a patient does not appear at the 12-month follow-up appointment and cannot be reached by telephone, the admission data recorded in the HIS is documented in items KS1c_0-KS1c_29 of the eCRF (a comparison with the clinic names is not necessary here, as the HIS information is always available in full). This information is shown in Table 1 represented by the HIS column. The last column indicates the value for the primary variable "full inpatient readmission rate" for the respective case.

Analogous to full inpatient readmission, the variables extended readmission (IEHT + full inpatient) and combined readmission (IEHT + full inpatient + partial inpatient) are also calculated within 12 months of admission to the index treatment. The IEHT admission data is determined by the items css056c-css059c and C041_05-C041_16 and a partial inpatient stay by the items css051c-css052c and C036_06- C036_07 of the CSSRI (or KS3c_0-KS3c_15 and KS2c_0-KS2c_8 for the HIS information). The hospital names of the IEHT stays can be found in the items css056ab-css059ab and C040_05-C040_16 and the day-case stays in css051ab-css052ab and C035_06- C035_07.

The same procedure is used for the variables for readmission within 6 months of inclusion in the index treatment. In addition, the date of the first readmission (min{css040c-css045c, C019_05-C019_28, C023_03-C023_08, KS1c_0-KS1c_29} for full inpatient, min{css056c-css059c, C041_05-C041_16, ks_31c-ks_34c, KS3c_0-KS3c_15} for IEHT and min{css051c-css052c, C036_06-C036_07, KS2c_0-KS2c_8} for partial inpatient readmissions) (only data after the discharge date may be taken into account) to determine whether the readmission took place in the first 6 months. For this purpose, it is checked whether the date of the first readmission is less than or equal to the date of signature of the consent form (eCRF item

EK03_01) + 6 months. If only a clinic is specified in a telephone survey, but no admission date, it only counts as a readmission if this was asked at the 6-month follow-up appointment. Otherwise, a Missing must be set here.

The procedure for readmission 6 months after discharge from the index treatment is analogous. The difference is that the date of the first readmission is compared with the discharge date from the index treatment (eCRF item A3_03) + 6 months.

10.3.7.2. Number of treatment days (after discharge from index treatment)

The number of days spent as an inpatient (in the study clinic) after discharge from the index treatment within 6 or 12 months after admission to the index treatment is also determined by the CSSRI. Here too (see **Fehler! Verweisquelle konnte nicht gefunden werden.**), only those full inpatient psychiatric stays are considered for which the admission date (eCRF items css040c-css045c, C019_05-C019_28 and C023_03-C023_08) is after the discharge date from the index treatment (eCRF item A3_03). In addition, the entry in the Clinic column (eCRF items css040ab-css045ab, C018_05-C018_28 and C022_03-C022_08) is compared with a list of names (created in collaboration with the study clinics) so that only stays in the study clinic are taken into account. For each of these stays, the difference between the discharge date (eCRF items css040d-css045d, C020_05-C020_28 and C024_03-C024_08) and the admission date is then calculated. The days spent in the various hospital stays are then totaled. It should be noted that if periods of different hospital stays overlap, the "double" days are only counted as one day. If a patient does not appear for the 12-month follow-up, the admission data (KS1c_0-KS1c_29) and discharge data (KS1d_0-KS1d_29) from the HIS are used instead for the missing period.

Similarly, the number of IEHT + full inpatient days spent (in the study clinic) after discharge from the index treatment within 6 or 12 months after admission to the index treatment is calculated. The clinic names, admission data and discharge data for IEHT stays are the eCRF items css056a-css059d and C040_05-C042_16 (or KS3a_0-KS3d_15 for the HIS information).

In addition, the variable number of days spent as an inpatient (outside the study clinic) after discharge from the index treatment within 12 months of admission to the index treatment is determined for a descriptive analysis. The calculation using the CSSRI data is carried out analogously, whereby only the stays that did not take place in the study clinic are taken into account. As the HIS data does not provide any information on this in most cases, missing

values may occur due to "lost to follow-up". An analogous procedure is used for IEHT + days spent as an inpatient (outside the study hospital).

10.3.7.3. Time until full inpatient readmission (after discharge from index treatment)

The time in days between discharge from the index treatment (eCRF item A3_03) and the first readmission to an inpatient psychiatric ward or clinic is calculated here.

The date of the first full inpatient readmission is determined via the minimum of the eCRF items css040c-css045c, C019_05-C019_28, C023_03-C023_08 and KS1c_0-KS1c_29. It should be noted that only stays for which the admission date is after the discharge date from the index treatment (eCRF item A3_03) are taken into account (cf. **Fehler! Verweisquelle konnte nicht gefunden werden.**). If no full inpatient readmission could be determined by the 12-month follow-up date, this is to be regarded as a right censoring. If the documentation was terminated earlier (e.g. in the event of death), the censoring should be set to this point in time.

10.3.7.4. Continuity of treatment

Treatment continuity after 6 or 12 months indicates whether outpatient psychiatric/psychotherapeutic services were used in the last 3 months at the respective point in time. The variable is set to 1 if one of the following services was used and an entry is therefore available in the respective eCRF item.

- Psychiatrist in private practice (css067ab)
- Psychological psychotherapist (css068ab)
- Psychiatric outpatient clinic (psychiatrist / psychotherapist) (css069ab)
- Psychotherapeutic outpatient clinics (css070ab)
- Social psychiatric service (css076ab)
- Day centers (incl. associated occupational therapy) (css077ab)
- Occupational therapy (css078ab)
- Crisis service (css079ab)
- Online consulting (css080ab)
- Self-help groups (css081ab)
- Community nurse (css082ab)
- Employee of a social center (css084ab)
- Psychiatric day care (css086ab)
- Outpatient addiction support (css092ab)
- Vocational rehabilitation (css094ab)

If there is no entry, the variable is set to 0.

10.3.8. Scores from questionnaires

The questionnaires 10.3.8.1-10.3.8.4 are collected immediately (baseline) and 6 and 12 months after admission to the index treatment. The questionnaires 10.3.8.5-10.3.8.7 are collected immediately after discharge from the index treatment.

10.3.8.1. Health-related quality of life according to EQ5D-5L

Health-related quality of life is determined using EQ5D-5L (Leidl & Reitmeir, 2017) (eCRF items EQ5D_1-EQ5D_5).

The EQ5D-5L is evaluated on the basis of the current standard values for the German general population. For this purpose, the measured values are linked with utility value estimates resulting from surveys of the general population. The "health profile" is explicitly determined descriptively and then model 3b (see Table 19 in the appendix 13.4 from Ludwig et al.) is used to calculate the index value. The index value (-0.661-1) is then used for the further evaluations.

If at least one of the 5 questions of the EQ5D-5L is not answered, the index value is considered missing (Simons C.L., Rivero-Arias O., Yu L-M. & Simon J., 2014).

10.3.8.2. Psychosocial functioning level according to HoNOS and PSP

The level of psychosocial functioning is determined using HoNOS (Frankhauser, Hochstrasser, Sievers & Soyka, 2017) and PSP (Morosini 2000). The two questionnaire scores are considered separately in the evaluation.

In the HoNOS (eCRF items honos01-honos12), the responses "no problem" to "severe to very severe problem" are coded 0-4. The HoNOS-D total burden (0-48) is then calculated as the sum of these values. An "unknown / not applicable" (coded as 9) is not included in the score and should therefore be assigned as rarely as possible (< 3).

With PSP, only the total score (0-100) from the eCRF item psp_total is used for the analysis.

10.3.8.3. Professional integration through CSSRI-D

Item css010a from the CSSRI is used to determine professional status, with the following possible answers:

1. Self-employed / Activity in the open labor market - full-time (css010a1)
2. Self-employed / Activity in the open labor market - part-time (css010a2)
3. Marginal employment (css010a4)
4. Unemployed / jobseeker (css010a5)

5. Disability or occupational disability (css010a6)
6. Old-age pension / pension / early retirement (css010a7)
7. Training / retraining (css010a8)
8. Housewife / Househusband (css010a9)
9. Voluntarily (unpaid) employed (css010a10)
10. Protected activity (css010a11)
11. Other (css010a12)

Answer options 1 and 2 form the category of the primary labor market. All other answer options indicate only conditional professional integration. If at least answer option 1 or 2 indicates that this applies, professional integration is coded as 1, otherwise as 0.

The change (between the baseline survey and the 12-month follow-up) is then calculated by dividing the 12-month value by the baseline value. This gives us an (ordinal) score that can take the values -1, 0, 1. This score is used in the further evaluation.

10.3.8.4. Recovery orientation according to RAS-G

Recovery orientation is determined using the RAS (Corrigan etr al., 1999) in the German translation (RAS-G) according to Cavelti et al. (2017) (eCRF items RAS_01-RAS_14).

The answers "Not true at all" to "Completely true" are coded 1-5. The total score is then calculated as the sum of the individual items. The value of the total score of each patient can therefore be a minimum of 14 and a maximum of 70.

If individual questions are omitted by a patient, the mean value from the answered questions is used instead of the missing value. If 8 or more answers are missing, no score is calculated and the variable is considered missing.

10.3.8.5. Treatment satisfaction according to the ZFP questionnaire

This section summarizes the ZFP questionnaires on treatment satisfaction with regard to the index treatment of patients (eCRF items Behazuf_staeb_01- Behazuf_staeb_18 for IEHT patients and Behazuf_stat_01- Behazuf_stat_18 for inpatients) and relatives (AZ02_01-AZ02_13).

The answers are coded from "strongly disagree" (=1 in the eCRF) to "strongly agree" (=5 in the eCRF) with 0, 25, 50, 75, 100. The overall satisfaction of a patient or a relative is then calculated as the mean value across the individual satisfaction items of a questionnaire.

If more than 50% of the questions in a questionnaire are not answered, the score is not calculated and is considered missing.

10.3.8.6. Burden on relatives according to IEQ-EU

The items are each to be marked on a 5-point Likert scale, the answers are coded 0-4 from "never" (=1 in the eCRF) to "almost always" (=5 in the eCRF).

In total, the main part of the IEQ-EU consists of 4 scales made up of 27 items:

Table 2Allocation of the IEQ-EU items to the various subscales

Subscale	N Items	Items	Score Range
1. Tension:	9	20, 21, 22, 23, 24, 25, 26, 33, 34	0-36
2. Control:	5	13, 14, 15, 16, 17	0-20
3. Concern:	5	28, 29, 30, 31, 32	0-20
4. Push:	8	7, 8, 9, 10, 11, 12, 18, 19	0-32
Sum score	27	7 - 26 and 28 - 34.	0-108

A score is calculated for each of the individual subscales by summing the associated item values. The total score can then be determined as the sum of the subscale values. If individual questions are omitted by a patient, the mean value from the answered questions is used instead of the missing value. If 14 or more answers are missing, no score is calculated and the variable is considered missing.

If it is stated in the general part of question 6 that there was less than one hour per week of contact (in person or by telephone) with the patient concerned during the period under review (sufficient contact = 0, see 10.3.5.3), the questions relating to current help and encouragement cannot be evaluated. This applies to items 7 - 27, in which case it is only possible to calculate a value for the "Concern" subscale.

The burden on relatives is assessed with the IEQ-EU immediately after discharge from the index treatment, once with reference to the last four weeks during the index treatment (eCRF items AB47-AB79) and once with reference to the last four weeks before admission to the index treatment (eCRF items AB09-AB44). The variable Baseline burden on relatives according to IEQ-EU indicates the score for the questionnaire that relates to the four weeks before admission to the index treatment. For the variable Reduction of relatives' stress according to IEQ-EU, the baseline IEQ score is subtracted from the IEQ score that relates to the last four weeks during the index treatment.

10.3.8.7. Perceived involvement in decisions according to SDM-Q-9

Perceived involvement in decisions is determined using SDM-Q-9 (eCRF items SDQ9_01-SDQ9_09) in the German translation according to Kriston et al. (2010).

The answers to the 9 items are coded from 0 to 5 from "strongly disagree" (=1 in the eCRF) to "strongly agree" (=6 in the eCRF). The scores of the individual items are added together to obtain an overall score with a range of 0 to 45. This score is then multiplied by $\frac{20}{9}$ to obtain a final score between 0 and 100 and thus simplify interpretation. A higher score thus indicates greater perceived involvement in treatment decisions.

If individual questions are omitted by a patient, the mean value from the answered questions is used instead of the missing value. If 3 or more answers are missing, no score is calculated and the variable is considered missing.

The additional question "Who made important medical decisions (e.g. about medication) as part of your treatment" is not included in the calculation of the overall score.

10.3.9. Dates

For dates (unless otherwise specified), the difference to the inclusion in the index treatment is calculated in days.

10.3.10. Pre-post variables

For pre-post variables, the pre-value is subtracted from the post value.

11. Statistical evaluation methods

The study pursues the research questions addressed in detail in Modules A to D, which are roughly described in the following list:

- Module A: Outcome research with quantitative evaluation
- Module B: Qualitative outcome and process research
- Module C: Process and implementation research
- Module D: Health economic research

This SAP comprises the statistical evaluation in Module E, which relates to the research questions of Module A and Module C. Module B is responsible for qualitative research and Module D for health economics research, each with its own competencies.

The statistical analysis is intended to examine the questions formulated in the (working) hypotheses of modules A and C. For this purpose, null hypotheses are formulated in each case, which are tested using statistical tests.

The number of observations, the test statistic and the p-value are calculated for each test performed. For regressions, the estimated value, the standard deviation and the p-value are given for each parameter. For linear and Cox regression, two-sided 95% confidence intervals are tabulated for the regressors and for logistic regression for the odds ratios. For correlations, unless otherwise described, only the strength of the correlation is reported. Other variables to be determined are stated directly in the description of the analyses concerned.

In addition to the evaluations described in SAP, a case wise listing (person-related digital list) is created with all recorded items. This can be used for further ad hoc analyses.

11.1. Demographic data and other baseline characteristics

Descriptive analyses are carried out for the demographic data and other baseline characteristics. This includes the variables from section 7.2.1 and the tertiary variables (see 7.2.3) and auxiliary variables (see 7.2.4) that are present at the baseline. The continuous variables are tabulated with the number of evaluable values (N), number of missing values (N miss), mean value (Mean), standard deviation (Std Dev), minimum (Min), first quartile (Q1), median, third quartile (Q3) and maximum (Max) according to the IEHT and full inpatient treatment (see Table 3 in the appendix 13.1). The categorical data is also tabulated according to the occupancy of the categories (including the missing category) in absolute terms and as

a percentage according to the IEHT and full inpatient treatment (see Table 4 in the appendix 13.1). For categorical data available at center level, the centers are assigned to the respective categories in a table (see Table 5 in Appendix 13.1). The variables of the structural type (see 10.3.2.2) are not included in the table for data protection reasons.

The quality of PS recruitment is tested by comparing the distributions of IEHT and control cases with regard to the variables age, gender, previous stays and diagnosis group (FX). For this purpose, Mann-Whitney tests for age and previous stays, a chi-squared test for gender and a Fisher test for the diagnostic groups with α = 5% are performed. If there is a significant difference, the respective variable is included in the models of the primary and secondary tests for Module A as a sensitivity analysis.

11.2. Statistics for quantitative outcome research (Module A)

The statistical evaluation of the research questions of Module A follows its two working hypotheses. The evaluation collective is the FAS. For all target criteria and covariates (which do not belong to the baseline variables), descriptive analyses are carried out analogous to the descriptive analyses for the baseline variables (see 11.1).

11.2.1. Primary outcome target criterion

First, the primary (working) hypothesis is that patients who were treated with IEHT as part of the AKtiV study have a significantly lower inpatient psychiatric readmission rate 12 months after admission to the index treatment than patients who received conventional inpatient treatment during the index stay.

11.2.1.1. Primary analysis (deductive)

The null hypothesis is that the two forms of treatment lead to the same full inpatient readmission rate 12 months after admission to the index treatment. As this is the only primary question, it is tested deductively with α = 5% (two-sided).

The primary target criterion of full inpatient readmission within 12 months of admission to the index treatment, together with the type of treatment (IEHT vs. full inpatient), forms a four-field table. It was therefore originally planned to test the null hypothesis with the chi-square test and, in the case of a small population (expected value of a field below 5), with the Fisher test. Since missing values of the primary criterion are now multiply imputed and this requires an (at

least) asymptotically normally distributed test statistic, the normal approximation of the 2-sample binomial test is used instead of the chi-square test.

A result with p below the significance level of $\alpha = 5\%$ is considered significant evidence of different readmission rates. In addition, the 95% confidence interval for the difference in readmission rates is calculated.

11.2.1.2. Sensitivity analyses (exploratory)

A series of sensitivity analyses are carried out for the primary target criterion in order to support the interpretation of the results.

First, the primary analysis is repeated with the per-protocol collective and the collective of survivors (all patients who are not known to have died before the 12-month follow-up date).

Then the primary analysis is performed again, but the missing data is not multiply imputed, but three possible scenarios are tested:

1. For all missing data from inpatients, it is assumed that they were readmitted as inpatients and for the IEHT patients that they were not readmitted (best case)
2. For all missing data from IEHT patients, it is assumed that they were readmitted as inpatients and for inpatients that they were not readmitted (worst case)
3. Only those patients are used for the evaluation for whom the variable full inpatient readmission is not missing (full case)

Prognostic factors are then taken into account in a logistic regression. The FAS is again used here. The following models are tested in abbreviated form (y refers to the readmission rate):

Model 1: y= constant + IEHT + PS value

Model 2: y= constant + IEHT + age + previous stays + gender

+ Diagnostic group (for logit model)

Model 2 considers variables that are significantly heterogeneous with respect to the treatment groups.

Model 3: y= constant + IEHT + center

For the center variable, only the overall significance of the center influence is evaluated. The effect sizes of the individual centers are not examined.

In addition, the PS pairs are assigned to two groups. The first group contains the pairs in which the true PS value is not close enough to each other (e.g. age entered incorrectly, incorrect diagnosis, etc.), the pairs in which no control patient was found for the IG patient after 5 months and an "emergency control patient" was recruited, and the pairs in which a protocol violation occurred in one of the two patients. The second group contains all pairs in which none of the cases listed occurred. If we denote this variable with X (X=1, if in the first group; X=0, if in the second group), the following logit model is tested:

Model 4: y= constant + IEHT + X + IEHT * X

The secondary analysis 11.2.2 also takes into account different observation times.

11.2.1.3. Further analyses of the primary criterion

Further analyses of the full inpatient readmission rate criterion result from the context of the research questions from Module C and are therefore presented in Section 11.3 is dealt with.

11.2.2. Secondary outcome target criteria (exploratory)

The evaluation of the secondary target criteria is based on the following research question of Module A: "Over the course of 12 months after admission to index treatment, do the combined readmission rate (full inpatient + partial inpatient + IEHT), the total number of days spent in full inpatient psychiatric care, treatment drop-outs decrease and do health-related quality of life, psychosocial functioning level, occupational integration, treatment satisfaction, perceived involvement in treatment decisions and recovery orientation increase in users receiving inpatient equivalent treatment compared to users receiving full inpatient psychiatric care?". In addition, hypotheses were formulated that emerged after consultation with Module A and Module C (see 11.2.2.2, 11.2.2.3 and 11.2.2.5).

The evaluation for the secondary criteria, which are recorded 6 months after admission to the index treatment, is purely descriptive (for the variables see 10.3.8). For this purpose, continuous variables with N, N miss, Mean, Std Dev, Min, Q1, Median, Q3 and Max are tabulated according to IEHT and full inpatient treatment. The categorical data are also tabulated according to IEHT and inpatient treatment in absolute and percentage terms after the categories have been filled (including the missing category) (analogous to Table 3 and Table 4 in the appendix 13.1). In addition, the variables full inpatient readmission 6 months

after discharge from the index treatment and treatment continuity 6 and 12 months after admission to the index treatment (see 10.3.7) are included in the descriptive evaluation.

In addition, the following explorative evaluations (two-sided with α = 5%) are planned.

11.2.2.1. Combined readmission rate

The null hypothesis is tested that the combined readmission rate 12 months after admission to the index treatment is the same in both treatment groups. The normal approximation of the 2-sample binomial test is used for this purpose. In addition to the usual variables, the 95% confidence interval for the difference in the combined readmission rates is reported.

11.2.2.2. Extended readmission rate

The null hypothesis is tested that the extended readmission rate 12 months after admission to the index treatment is the same in both treatment groups. The normal approximation of the 2-sample binomial test is used for this purpose. In addition to the usual variables, the 95% confidence interval for the difference in the combined readmission rates is reported.

11.2.2.3. Time until full inpatient readmission

The null hypothesis that the time to full inpatient readmission after discharge from the index treatment is the same in both treatment groups is tested. First, the time to readmission is analyzed graphically using a Kaplan-Meier estimate. To test the null hypothesis, a Cox model is created with the treatment group as a covariate. The null hypothesis is specified in this model as regression parameter=0.

11.2.2.4. Number of days spent as an inpatient

The null hypothesis is tested that the number of full inpatient days in the study hospital after discharge from the index treatment (see 10.3.7.2) 12 months after admission to the index treatment is the same in both treatment groups. The non-parametric Mann-Whitney test is used.

As a descriptive analysis, the number of full inpatient stays outside the study clinic after discharge from the index treatment within 12 months of admission to the index treatment (see 10.3.7.2), a table analogous to Table 3 in the appendix 13.1 has been created.

11.2.2.5. Number of IEHT + full inpatient days spent

The null hypothesis is tested that the number of IEHT + full-inpatient days in the study hospital after discharge from the index treatment (see 10.3.7.2) 12 months after admission to the index

treatment is the same in both treatment groups. The non-parametric Mann-Whitney test is used.

As a descriptive analysis, the number of IEHT + full-inpatient days outside the study clinic after discharge from the index treatment within 12 months after admission to the index treatment (see 10.3.7.2) a table analogous to Table 3 in the appendix 13.1 has been created.

11.2.2.6. Canceling the index treatment

The null hypothesis that the dropout rate of the index treatment is the same in both treatment groups is tested. The chi-square test or Fisher test is used for small field populations (expected value of a field less than 5).

In a sensitivity analysis, the reasons for dropping out of the index treatment are also analyzed. First, a table is created with the number of discontinuations broken down by the various reasons for the descriptive analysis (see Table 11 in the appendix 13.1).

In addition, a contingency table (2 x number of different reasons) with the treatment groups as rows and the reasons for discontinuing the index treatment as columns is exploratively tested for independence using the chi-square test or Fisher test for small field populations (expected value of a field less than 5)

11.2.2.7. Health-related quality of life

The null hypothesis is tested that the health-related quality of life according to EQ5D-5L (see 10.3.8.1) is the same in both treatment groups 12 months after inclusion in the index treatment. The non-parametric Mann-Whitney test is used for this purpose.

11.2.2.8. Psychosocial level of functioning

The null hypothesis to be tested is that the psychosocial functioning level according to HoNOS and PSP (see 10.3.8.2) is the same in both treatment groups 12 months after admission to the index treatment. We use the Welch test for the HoNOS and the non-parametric Mann-Whitney test for the PSP. The Welch test is used for HoNOS because data from other studies (see Luka 2013) suggest a normal distribution and the Welch test is robust to small deviations from the normal distribution.

11.2.2.9. Professional integration

The null hypothesis to be tested is that the change in occupational integration through CSSRI-D (see 10.3.8.3) 12 months after admission to the index treatment is the same in both treatment

groups. For this purpose, an ordinal logistic regression is carried out with the change in occupational integration as the target variable and the treatment group as the covariate.

As a sensitivity analysis, the number of patients with a specific occupational status on admission and 12 months after admission to the index treatment is tabulated according to the two treatment groups (see Table 7 in the appendix 13.1).

11.2.2.10. Recovery orientation

The null hypothesis is tested that the recovery orientation according to RAS-G (see 10.3.8.4) is the same in both treatment groups 12 months after admission to the index treatment. The Welch test is used for this purpose. The normal distribution assumption is justified by the central limit theorem (the score is formed by summing many items). In addition, the Welch test is robust to small deviations from the normal distribution.

11.2.2.11. Perceived involvement in decisions

The null hypothesis is tested that the perceived involvement in decisions according to SDM-Q-9 (see 10.3.8.7) is the same in both treatment groups immediately after discharge from the index treatment. The non-parametric Mann-Whitney test is used.

11.2.2.12. Treatment satisfaction

The null hypothesis is tested that the treatment satisfaction of patients according to the ZFP questionnaire (see 10.3.8.5) with regard to the index treatment immediately after discharge from the index treatment is the same in both treatment groups. The Welch test is used for this purpose. The normal distribution assumption is justified using the central limit theorem (and robustness of the Welch test to small deviations).

11.2.2.13. Sensitivity analyses for the secondary target criteria

All analyses are performed again with the PP-collective. In addition, the same analysis is carried out again as a full case analysis for all hypotheses in which the target variable is imputed. As a further sensitivity analysis, the analyses 11.2.2.7-11.2.2.10 and 11.2.2.11-11.2.2.12 were carried out again, whereby values that were considered missing because the follow-up date was outside the +-14 day or +7 day interval were included as true values this time.

In addition, for all secondary target criteria, analogous to model 2 from 11.2.1.2the regression models described below are created for all secondary target criteria. Due to the nature of the target variables, a distinction must be made between the following cases.

A logit model is created for the target criteria combined readmission, extended readmission, change in occupational integration and discontinuation of the index treatment (occupational integration is an ordinal logit model).

A Cox model is used for the period until resumption.

For the number of days spent as an inpatient in the study clinic after discharge from the index treatment, the patients are divided into five groups and an ordinal logit model is created:

1. Number of days spent as a full inpatient equals 0
2. Number of days spent as a full inpatient greater than 0 and less than or equal to 15
3. Number of days spent as a full inpatient greater than 15 and less than or equal to 32
4. Number of days spent as a full inpatient greater than 32 and less than or equal to 76
5. Number of days spent as a full inpatient greater than 76

The grouping was based on the quantiles from the 2019 data of the centers. These were collected independently of this study.

The following categorization is selected for the number of IEHT + full inpatient days (in the study clinic) and an ordinal logit model is created:

1. Number of IEHT + full inpatient days spent equals 0
2. Number of IEHT + fully inpatient days greater than 0 and less than or equal to 19
3. Number of IEHT + full inpatient days spent greater than 19 and less than or equal to 41
4. Number of IEHT + full inpatient days spent greater than 41 and less than or equal to 85
5. Number of IEHT + full inpatient days spent greater than 85

The logit models were chosen instead of linear models, as the data mentioned suggest that the number of (IEHT +) days spent in full inpatient care is not normally distributed.

A linear model is created for treatment satisfaction.

A linear model is created for the psychosocial functioning level (according to HoNOS) and the recovery orientation. In addition, the baseline value of the corresponding target variable is taken into account as a covariate in the model.

For health-related quality of life and psychosocial functioning (according to PSP), the patients are grouped according to the 12-month values of the corresponding variable and an (ordinal) logit model is created. The limits for the groups are calculated as follows on the basis of the baseline values:

1. Group: Score less than or equal to 25% quantile
2. Group: Score greater than 25% Quantile less than or equal to median
3. Group: Score greater than median less than or equal to 75% quantile
4. Group: Score greater than 75% quantile

The baseline value of the corresponding target variable is taken into account as an additional covariate in the model.

The same approach is used for perceived inclusion in decisions, whereby the data collected immediately after discharge from the index treatment is used to determine the group boundaries and for group allocation. In addition, there is no baseline value that could be included in the model.

11.2.3. Tertiary outcome target criteria (descriptive)

In a tertiary evaluation, the influence of the tertiary variables (see 10.3.4) and the PS variables (age, previous stays, gender and diagnosis group (FX)) on the full inpatient readmission rate in addition to the classification into the treatment groups is to be analyzed.

First, the full inpatient readmission rate in the individual groups is tabulated for the categorical variables according to the type of treatment (see Table 9 in the appendix 13.1). For the diagnosis (FXY), all diagnoses to which no patient was assigned are omitted. For the continuous variables, N, Mean, Std Dev and Median are tabulated according to the treatment groups and the primary criterion (see Table 8 in the appendix 13.1).

In addition, the variables are considered in a logistic regression. The following model is tested in abbreviated form:

Full inpatient readmission rate = constant + treatment group + age + previous stays + gender + diagnosis group (for logit model) + place of residence (urban-rural) + employment situation

+ relocation (yes/no) + age at first full inpatient stay + total previous full inpatient stays + duration of previous treatment

Only the regression coefficient and the odds ratio (incl. p-value and 95% confidence interval) for the treatment group variable are calculated and interpreted.

In addition, the following logit model with interaction terms is tested for each of the covariates from the previous model (except the IEHT variable):

Full inpatient readmission rate = constant + treatment group + variable + variable * treatment group

11.3. Statistics on process and implementation research (Module C)

In all further evaluations concerning the target criteria of the process and implementation research (Module C), only the IEHT patients are considered, i.e. the IEHT collective is taken as the basis for the patients. As Module C provided a series of (working) hypotheses to be tested, this chapter is not divided according to target criteria, but according to hypotheses.

The employee evaluations are described separately in chapter 11.3.2 are described separately. The employe collective is used as the basis there.

As the primary research question in Module A is already tested deductively and Module C partly examines the same target variables, all analyses relating to Module C are conducted exploratively. Therefore, no α-adjustment is necessary and testing is always carried out at the level $\alpha = 5\%$ (two-sided). The exploratory nature of the results must be pointed out in the report.

For all target criteria and covariates (which are not baseline variables), descriptive analyses are performed analogous to the descriptive analyses for the baseline variables (see 11.1). If a variable is only recorded for IEHT patient, the line for full inpatients is omitted. Employee variables are excluded from this analysis. Here, the descriptive analyses to be performed are described in the sections themselves.

11.3.1. Patient analyses in Module C (exploratory)

11.3.1.1. Relationship between the organizational structure and treatment

It is based on the (working) hypothesis that there is a close connection between the organizational structure of the treatment teams and the treatment received by the patients.

The organizational structure of a treatment team is defined by the four variables of the structure type (see 10.3.2.2). The treatment provided to a patient is divided into the target variables treatment duration per day across all professional groups, treatment duration per day per professional group, number of contacts per day and treatment duration of the index treatment in days and is recorded at patient level.

The following null hypotheses are tested:

1. The duration of treatment per day is the same in all organizational structures of the treating teams
2. The number of contacts per day is the same in all organizational structures of the treating teams
3. The duration of treatment per day by physicians is the same in all organizational structures of the treating teams
4. The duration of treatment per day by psychologists is the same in all organizational structures of the treating teams
5. The duration of treatment per day by specialized therapists is the same in all organizational structures of the treating teams
6. The duration of treatment per day by nursing staff is the same in all organizational structures of the treating teams
7. The treatment duration of the index treatment in days is the same in all organizational structures of the treating teams

Hypotheses 1-6 can be tested using multiple linear regression (the normal distribution assumption is justified by the central limit theorem and the robustness of linear regression in the case of small deviations from the normal distribution). In the models, in addition to the four binary variables that describe the structure types (see 10.3.2.2), the treatment duration of the index treatment is taken into account as an additional covariate. In short form, the following model is tested for each of the 6 target variables:

y= constant + organization of the department + team size + type of clinic + care region + treatment duration of the index treatment

When interpreting the results of the second hypothesis, it should be noted and pointed out in the report that in the data to be analyzed, several contacts of the same occupational group on one day are only shown as one contact with a total treatment duration.

Since the target variable of the 7th hypothesis is not normally distributed according to the 2019 data, the patients are divided into groups and a logit model is created. The following groups were calculated using the quantiles:

1. All patients with a maximum index treatment duration of 15 days
2. All patients with an index treatment duration of more than 15 and a maximum of 30 days
3. All patients with a treatment duration of the index treatment of more than 30 and a maximum of 50 days
4. All patients with a treatment duration of more than 50 days

In order to compare the effect sizes of the four variables of the structural type with each other, the regression coefficients with standard deviation are shown graphically for each of the seven models.

11.3.1.2. Influence of employee job satisfaction on the satisfaction of patients and their relatives

It is based on the (working) hypothesis that job satisfaction in the IEHT teams correlates with treatment satisfaction among patients and relatives in a study center-specific manner.

Job satisfaction in the IEHT teams is described by two different scores, which are derived from parts of the COPSOQ and an ZFP questionnaire (for the exact calculation of the scores, see 10.3.6.1). Treatment satisfaction is determined by the ZFP questionnaire from previous dissertations with modifications of Module C.

The following null hypotheses are tested:

1. Patient treatment satisfaction is independent of average job satisfaction at the treating center
2. The treatment satisfaction of relatives is independent of the average job satisfaction in the treating center

To test the hypotheses, a multiple linear regression model is created with the treatment satisfaction of the patients (or relatives) as the target variable and the average job satisfaction in the treating center as a covariate (one covariate for each of the two job satisfaction scores). The normal distribution assumption for the treatment satisfaction scores is based on the central limit theorem (and robustness of the linear regression to small deviations).

As a sensitivity analysis, the same analysis is carried out again, whereby patient satisfaction values that were considered missing because the follow-up appointment was outside the +7 day interval are this time taken into account as true values.

11.3.1.3. Relationship between the type of treatment and the burden on relatives

The (working) hypothesis is that the burden on relatives of IEHT patients will be reduced more between the two study periods than the burden on relatives in the control group.

The burden on relatives is determined using the Involvement Evaluation Questionnaire (IEQ-EU) (see 10.3.8.6), the reduction by subtracting the baseline value.

As the (working) hypothesis assumes a difference between the two treatment groups, the FAS collective is used as an exception for the patients in this evaluation.

The null hypothesis is that the reduction in the burden on relatives is the same in both treatment groups.

It is to be expected that the baseline burden on relatives also has a considerable influence on the reduction in burden. In addition, the same differences can be interpreted very differently if they are based on very different prevalence values. For this reason, the baseline burden on relatives is included in the model as an additional variable.

In addition, the treatment satisfaction of the relatives (see 10.3.8.5) is taken into account as a covariate in the model.

As the IEQ-EU total score (see 10.3.8.6) is formed as a sum score of 27 variables, we assume that the score (and thus also the reduction in the burden on relatives) fulfills the normal distribution assumption (moreover, the linear regression is robust to small deviations). Therefore, the following model (in short form) is tested for the total score of the IEQ-EU using linear regression:

Reduction in burden on relatives = constant + IEHT + baseline burden on relatives + treatment satisfaction

As a sensitivity analysis, the same linear model is used again for each of the four subscales of the IEQ-EU (see 10.3.8.6) is descriptively tested as a target variable. Accordingly, the baseline value of the corresponding subscale is included in the model as a covariate (instead of the baseline burden on relatives).

In addition, the correlation between the treatment group and the relatives-variable contact sufficient is analyzed (see 10.3.5.3) using the chi-square test (one test for before treatment and one test for during treatment). If one of the tests is significant at $\alpha = 5\%$, the main analysis on the burden on relatives is carried out again as a sensitivity analysis, whereby the variable contact time (see 10.3.5.4) is included as a covariate in the model for the significant period.

In addition, the patients are divided into two groups (IEQ-EU score missing; IEQ-EU score present) and a homogeneity test is carried out with regard to treatment group, age, gender, previous stays and diagnosis group (FX). For this purpose, Mann-Whitney tests for age and previous stays, a chi-squared test regarding treatment group and gender and a Fisher test regarding the diagnosis groups with $\alpha = 5\%$ are performed.

11.3.1.4. Influence of a hybrid/autonomous team on the readmission rate and number of days with full inpatient treatment

The (working) hypothesis is based on the assumption that the implementation of IEHT as part of a ward-integrated team compared to a detached/autonomous IEHT-team is associated with a greater reduction in the inpatient readmission rate and the number of days spent as an inpatient during the study period.

As can be seen from the working hypothesis, the original plan was to consider the binary variable ward-integrated vs. autonomous team. However, it was concluded from the expert interviews that the classification into hybrid vs. autonomous team makes more sense (see 10.3.2.1).

The following null hypotheses are to be tested in detail:

1. Hybrid and autonomous IEHT-teams lead to the same full inpatient readmission rate.
2. Hybrid and autonomous IEHT-teams lead to the same number of days spent as inpatients (in the study hospital) after discharge from the index treatment.

The first null hypothesis is examined using normal approximation of the 2-sample binomial test. The non-parametric Mann-Whitney test is used for the second hypothesis.

11.3.1.5. Influence of individual treatment and patient variables on the readmission rate and number of days with full inpatient treatment

It is based on the (working) hypothesis that aspects of individual treatment show a stronger correlation to the full inpatient readmission rate and the number of days with full inpatient treatment than patient variables.

All analyses performed in this section are carried out as full-case analyses.

Aspects of individual treatment include the following variables:

1. Number of contacts from the treatment team per day
2. Treatment duration of the index treatment in days
3. Treatment duration per day across all occupational groups
4. Treatment duration per day per occupational group (a single variable for each occupational group)
5. Stability of treatment (overall score)
6. Average process evaluation in the treating center
7. Average job satisfaction in the treating center (one variable each for ZUF-1 and COPSOQ B11, see 10.3.6.1)

The patient variables include these variables:

8. Diagnostic group (FX)
9. Age
10. Gender
11. Stability of treatment (overall score)
12. Individual access route (IEHT transfer or IEHT direct)

It should be noted that the stability of the treatment is included in both aspects. This can be explained by the fact that the stability of the treatment can depend both on the practitioners and the associated treatment, as well as on the patients themselves.

First, a descriptive method is used to gain an overview of the extent to which the individual variables, as well as the individual treatment and the patient variables as a whole, influence the readmission rate.

For this purpose, a logit model is created for each of the variables 1-12 with the readmission rate as the target variable and the respective variable as the regressor. It should be noted that the diagnostic group (for logit model) is used in the models instead of the diagnostic group (FX). The corrected coefficient of determination R^2 and the Akaike information criterion (AIC) are then calculated for each of these models and the results are presented in a table. Two multiple logit models are then created with the readmission rate as the target variable. In one, variables 1-7 act as covariates (model 1) and in the other, variables 8-12 (model 2). In addition,

a model is created with variables 1-5 as covariates. The corrected coefficient of determination R^2 and the AIC are also calculated for these models.

The R^2 and AIC values obtained in this way can then be interpreted and compared with each other. The higher the R^2 value of a regression, the better the model can explain the fluctuations in the data. A higher R^2 value therefore indicates a greater correlation between the covariates and the model's target variable. With AIC, on the other hand, the model with the lowest AIC value is preferred.

In addition to this descriptive method, the Vuong test (Vuong, 1989) is also used. This is used to test the null hypothesis that models 1 and 2 are equally close to the true distribution exploratively (two-sided) at the level $\alpha = 5\%$. This test is similar to the likelihood ratio test, but can be used for non-hierarchical models.

In addition, all variables (1-12) are taken into account in another model. Here, too, the R^2 and the AIC are compared with the values of the other models. In addition, the model is compared with models 1 and 2 using the likelihood ratio test.

The same analysis (but with ordinal logit models) is repeated for the number of days with full inpatient treatment (after discharge from the index treatment) instead of the readmission rate. For this purpose, the patients are divided into the following groups and ordinal logit models are created:

1. Number of days spent as an inpatient (in the study hospital) equals 0
2. Number of days spent as an inpatient (in the study hospital) greater than 0 and less than or equal to 16
3. Number of days spent as an inpatient (in the study hospital) greater than 16 and less than or equal to 32
4. Number of days spent as an inpatient (in the study hospital) greater than 32 and less than or equal to 84
5. Number of days spent as an inpatient (in the study hospital) greater than 84

The grouping is based on the 2019 data, so that groups 2-5 are roughly the same size.

As an extension, the entire analysis is carried out again descriptively, with the difference that the process type of the treating center (see 10.3.2.3) is considered instead of variables 1-7. The underlying (working) hypothesis is that center effects with regard to the effects (full inpatient readmission rate and number of days spent in full inpatient care) can be explained by

differences in the treatment processes in the various centers rather than by their different case mix (diagnosis group, age, duration of illness).

11.3.1.6. Influence of the instability of a course on the readmission rate and the number of days with full inpatient treatment

It is based on the (working) hypothesis that unstable courses during index treatment are unfavorable predictors for outpatient treatment and the risk of full inpatient readmission.

The following null hypotheses can be derived from the (working) hypothesis:

1. The instability of the course of treatment has no influence on the readmission rate
2. The instability of the course of treatment has no influence on the number of days with full inpatient treatment after discharge from the index treatment

Unstable courses during the index treatment are described by the variable stability of the treatment (total score) with the two subscores stability details and discontinuation of the index treatment (for the determination of the variables see 10.3.3.).

Both null hypotheses are first tested using (ordinal) logistic regression (the variable number of days with full inpatient treatment is not normally distributed). In both models, the stability of treatment serves as a covariate. For the second model, the patients are divided into the following groups:

1. Number of days spent as a full inpatient (in the study hospital) equals 0
2. Number of days spent as a full inpatient (in the study hospital) greater than 0 and less than or equal to 16
3. Number of days spent as a full inpatient (in the study hospital) greater than 16 and less than or equal to 32
4. Number of days spent as a full inpatient (in the study hospital) greater than 32 and less than or equal to 84
5. Number of days spent as a full inpatient (in the study hospital) greater than 84

The grouping is based on the 2019 data, so that groups 2-5 are roughly the same size.

As a sensitivity analysis, the influence of the two subscores (stability details and discontinuation of the index treatment) on the two target variables is examined again individually in a descriptive manner.

For the influence of stability details on the two target variables (readmission rate and number of days with full inpatient treatment after discharge from the index treatment), (ordinal) logistic regression is also used (analogous to the models above). The correlation between the readmission rate and the discontinuation of the index treatment is tested with the normal approximation of the 2-sample binomial test. The influence of discontinuation of the index treatment on the number of days spent as an inpatient (in the study hospital) after discharge from the index treatment is determined using the Mann-Whitney test.

As a further sensitivity analysis, two multiple logit models with the individual instability variables that make up the treatment stability variable (see 10.3.3) as covariates and the two target variables from the hypotheses.

11.3.2. Evaluation of the employee survey (exploratory) in module C

11.3.2.1. Descriptive analysis of job satisfaction in the centers

The ZUF-1, the job satisfaction according to COPSOQ B11 (see 10.3.6.1) and the individual category scores of the employee survey (see 10.3.6.2) are tabulated according to the centers (see Table 12 in the appendix 13.1).

The center names are to be replaced by pseudonyms. An assignment of the pseudonyms to the individual study centers should only be available to the research group of module C1. Here it is determined which of the employees require access to this list and can therefore assign the respective job satisfaction to the individual study centers.

11.3.2.2. Influence of occupational group affiliation on job satisfaction

The null hypothesis to be tested is that job satisfaction in IEHT does not depend on occupational group affiliation.

For this purpose, small occupational groups are initially summarized in order to protect the data privacy of the employees: If the items of a category score or satisfaction score were answered by fewer than 5 employees in an occupational group, this occupational group is combined with the group "other". If there are still fewer than 5 employees in the "other" group, the occupational group with the fewest employees is also combined with the "other" group. In general, it should be noted that only as few groups are combined as necessary.

The null hypothesis is then tested using linear regression. One regression is carried out for the ZUF-1 and one for the COPSOQ B11 as the target variable (for the calculation of the scores,

see 10.3.6.1). The normal distribution assumption is justified by the fact that the scores are calculated as the sum of several items and the linear regression is robust to small deviations from the normal distribution.

In addition, the mean category and satisfaction scores (with the number of values from which the mean value is calculated) are tabulated by occupational group (see Table 13 in the appendix 13.1).

11.3.2.3. Differences in implementation satisfaction at the study centers

The null hypothesis that there are no differences in implementation satisfaction at the study centers needs to be tested.

Implementation satisfaction is measured by the process evaluation score (see 10.3.6.3). The null hypothesis is then tested using linear regression. The normal distribution assumption is justified with the central limit theorem (and robustness of the linear regression to small deviations).

11.3.2.4. Correlation of center-specific satisfaction with the introduction of IEHT and job satisfaction.

The correlation between job satisfaction (in each case for ZUF-1 and COPSOQ) and process evaluation per center is calculated using Spearman rank correlation (for calculation of the scores, see 10.3.6.1 and 10.3.6.3). The correlations are tabulated by center together with the number of observations considered (similar to Table 12).

11.3.2.5. Correlation between years in the profession and job satisfaction

The null hypothesis that the number of years in the profession has no influence on the satisfaction of employees at IEHT needs to be tested.

A linear regression is carried out for the ZUF-1 and COPSOQ B11 as target variables. The regressors used are professional experience, professional experience in outreach services and professional experience in IEHT (see 10.3.6.6).

11.3.2.6. Relationship between age and job satisfaction

The null hypothesis that age does not correlate with job satisfaction needs to be tested.

The correlation is calculated using Spearman's Rho (in each case for the ZUF-1 and the COPSOQ B11). In addition, a p-value is determined for the correlation.

11.3.2.7. Connection between gender and job satisfaction

The null hypothesis that gender has no influence on job satisfaction needs to be tested.

If fewer than 5 employees belong to the diverse group, they are excluded from the evaluation.

The Welch test is used to test the null hypothesis (one test each for the ZUF-1 and the COPSOQ B11).

11.3.2.8. Descriptive analysis of the ZUF-1 score

N, N miss, Mean, Std Dev, Min, Q1, Median, Q3 and Max of the individual items (across all employees) from which the ZUF-1 is formed and the total score are tabulated (see Table 14 in the appendix 13.1).

11.3.2.9. Influence of the structure type on job satisfaction

The null hypothesis that the structure type has no influence on job satisfaction needs to be tested.

For this purpose, a multiple linear model is created for both the ZUF-1 and the COPSOQ B11, in which the four variables that describe the structure type (see 10.3.2.2) serve as covariates.

In order to compare the effect sizes of the four variables with each other, the regression coefficients with standard deviation are also shown graphically.

11.3.2.10. Influence of a hybrid/autonomous team on job satisfaction

The null hypothesis that job satisfaction is just as high in a hybrid team as in an autonomous team needs to be tested.

The Welch test is used for this purpose (one test each for the ZUF-1 and the COPSOQ B11).

11.3.2.11. Influence of the structure type on the experience of team processes

The null hypothesis that the structure type has no influence on the experience of the team processes is to be tested (for the calculation of the score see 10.3.6.4).

To test the null hypothesis, a multiple linear model is created in which the four variables describing the structure type (see 10.3.2.2) serve as covariates. The normal distribution assumption is justified with the central limit theorem (and robustness of the linear regression to small deviations).

In order to compare the effect sizes of the four variables with each other, the regression coefficients with standard deviation are also shown graphically.

11.3.2.12. Influence of a hybrid/autonomous team on process evaluation

The null hypothesis that in a hybrid team the process evaluation (see 10.3.6.3) is just as high as in an autonomous team.

The Welch test is used for this purpose.

11.3.2.13. Descriptive analysis of satisfaction in the structure types

As with the center variable (see 11.3.2.1), the descriptive analysis of satisfaction in the structure types is based on the Table 12 (see Appendix 13.1) for each of the four variables of the structure type (see 10.3.2.2).

11.3.2.14. Descriptive analysis of the employee survey at item level

For each of the categories (see 10.3.6.2), a table is created with the mean values of the individual items and the category score according to the centers (analogous to Table 12 in the appendix 13.1). Instead of the center names, the pseudonyms from 11.3.2.1 are to be used.

In addition, a table of item mean values by occupational group is presented for each category analogous to Table 13 (see Appendix 13.1) for each category. Here, the occupational groups are organized as in 11.3.2.2 are summarized.

11.3.2.15. Descriptive analysis of demographic data

For each of the questions A1-A6 as well as Z1, Z3 and Z5 of the employee questionnaire, a table with the response frequencies by center is created (see Table 15 in the appendix 13.1).

For question Z2 and the two items of Z4, a table with N, N miss, Mean, Std Dev, Min, Q1, Median, Q3 and Max is created for each center (see Table 16 in the appendix 13.1).

11.3.3. Sensitivity analyses (exploratory) in module C

The following sensitivity analyses are carried out equally for each of the tested (working) hypotheses.

11.3.3.1. IEHTPP collective

All analyses from 11.3.1 are carried out again with the IEHTPP collective.

11.3.3.2. Full Case Analysis

For all hypotheses in which the target variable is imputed, the same analysis is performed again as a full case analysis.

11.3.3.3. Correlation of the independent variables

All independent variables of a tested (working) hypothesis are examined in pairs for correlations using Spearman rank correlation. One exception is the hypothesis on the relationship between the type of treatment and the burden on relatives. Here, the variables treatment type and baseline burden are not examined for a correlation.

11.3.3.4. Correlation of the target variables

All target variables of a tested (working) hypothesis are examined in pairs for correlations using Spearman rank correlation.

11.3.3.5. Extended readmission rate and number of days spent as an IEHT + full inpatient (in the study hospital) after discharge from the index treatment

All analyses in which the full inpatient readmission rate or the number of full inpatient days (in the study hospital) after discharge from the index treatment are examined are carried out analogously for the extended readmission rate and the number of IEHT + full inpatient days (in the study hospital) after discharge from the index treatment. If the patients are grouped for analysis according to the number of IEHT + full inpatient days, the following classification (according to the 2019 data) is selected:

1. Number of IEHT + full inpatient days (in the study hospital) equals 0
2. Number of IEHT + full inpatient days (in the study hospital) greater than 0 and less than or equal to 22
3. Number of IEHT + full inpatient days (in the study hospital) greater than 22 and less than or equal to 47
4. Number of IEHT + full inpatient days (in the study hospital) greater than 47 and less than or equal to 101
5. Number of IEHT + full inpatient days (in the study hospital) greater than 101

11.3.4. Testing validity and reliability

Cronbach's alpha is used to determine the internal consistency of the questionnaires used. This is calculated for the items of each subcategory from the employee survey (see 10.3.6), the items of the ZUF-1, the categories of the ZFP employee questionnaire, the items of the treatment stability score (10.3.3.5) and the treatment satisfaction items (10.3.8.5).

To check the validity of the ZUF-1, Spearman's rank correlation between the ZUF-1 and the COPSOQ B11 score is calculated. A p-value and 95% confidence interval are reported.

11.4. Safety variables

All SAEs are considered safety variables. For descriptive analysis of these events, the SAEs are tabulated according to the treatment groups (see Table 10 in the appendix 13.1).

11.5. Multicenter data

Multicenter data are taken into account in Module A by means of logistic regression in the sensitivity analysis of the primary evaluation. In Module C, multicenter data are considered in some analyses. In the descriptive analysis for Module C, variables are (partially) tabulated according to the centers.

11.6. Dealing with multiple comparisons

It is only tested deductively for the primary target criterion in module A, which is why there is no multiple comparison situation and therefore no α-adaptation is necessary.

Further tests should only be interpreted in an exploratory sense. They are formally carried out with $\alpha = 5\%$.

11.7. Interim analyses

Interim analyses are not planned.

11.8. Stratification

The study was implicitly stratified by PS recruitment according to diagnostic group FX and PS value. The diagnostic groups and PS values are taken into account by means of logistic regression in the evaluation of the primary criterion (sensitivity analyses).

11.9. Subgroup analyses

As further relevant questions will also arise in the course of the AKtiV research project, which cannot be considered in this list at the present time, these will also be included and examined retrospectively. Since the AKtiV study, as a large-scale research project in the context of health services research, is itself designed to generate hypotheses, this must be taken into account in the course of the study.

12. Bibliography

Daniel R., White I.R. & Royston P. (2010). *Avoiding Bias Due to Perfect Prediction in Multiple Imputation of Incomplete Categorical Variables.* Computational Statistics and Data Analysis, 54, 2267-2275.

Gluud C., Jakobsen J.C., Wetterselv J., Winkel P. (2017). *When and how should multiple imputation be used for handling missing data in randomized clinical trials - a practical guide with flowcharts.* BMC Medical Research Methodology*, 17,* 162.

John O. & Onu J. (2018). *Application of asymptotic distribution of Mann-Whitney statistic to determine the difference between the systolic blood pressure of men and women over 45 years of age.* European Journal of Mathematics and Computer Science, 5(2), 9-13*.*

Kriston L., Scholl I., Holzel L., Simon D., Loh A. & Harter M. (2010). *The 9-item Shared Decision Making Questionnaire (SDM-Q-9*). Development and psychometric properties in a primary care sample*.* Patient Educ. Couns. 80(1), 94-99.

Little R. & Rubin D. (2002). *Statistical Analysis with Missing Data*. Hoboken, USA: Wiley & Sons.

Luka S. (2013). *Behandlungsergebnis stationärer psychiatrischer Behandlung aus verschiedenen Beurteilerperspektiven*.

Ludwig K., et al. (2018). *German Value Set for the EQ-5D-5L.* PharmacoEconomics, 36*,* 663-674.

Mehrotra D.V. & Mogg R. (2007). *Analysis of antiretroviral immunotherapy trials with potentially non-normal and incomplete longitudinal data*. Statistics in Medicine, 26, 484-497.

Simons C.L., Rivero-Arias O., Yu L-M. & Simon J. (2014). *Multiple imputation to deal with missing EQ-5D-3L data: Should we impute individual domains or the actual index?* Basel: Springer international Publishing.

Vuong Q.H. (1989). *Likelihood Ratio Tests for Model Selection and Non-Nested Hypotheses*. Econometrica, 57(2), 307-333.

Weinmann, S., Spiegel, J., Baumgardt, J., Bühling-Schindowski, F., Pfeiffer, J., Kleinschmidt, M., & Bechdolf, A. (2022). Stationsäquivalente Behandlung (StäB) im Vergleich mit vollstationärer Behandlung: 12-Monats-Follow-up einer gematchten Kohortenstudie. *Psychiatrische Praxis*, *49*(08), 405-410.

13. Appendix

13.1. Structure and listing of the tables

Table 3: Example of the descriptive table of a continuous variable

Age	N	N miss	Mean	Std Dev	Min	Q1	Median	Q3	Max
IEHT									
Fully stationary									
Total									

Table 4: Example of a descriptive table for a categorical variable

Diagnostic group (FX)	Missing		F0X		F1X		F2X		F3X		F4X		F5X		F6X		F7X	
	N	(%)	N	(%)	N	(%)	N	(%)	N	(%)	N	(%)	N	(%)	N	(%)	N	(%)
IEHT																		
Fully stationary																		
Total																		

Table 5: Example of a descriptive table for a categorical variable at center level. The category to which the corresponding center belongs is marked in the cells.

	Center										Total	
	CHA	KAU	KNK	ZWI	REI	HOW	KBO	TUB	REU	RUD	N	Pct N
**Departmental organization**												
Specialist expertise from department												
Specialist expertise isolated in the IEHT team												
**Team size**												
Total sales > 10												
Total sales price < 10												
**Supply region**												
Million strong Metropolis												
Other												
**Type of clinic**												
University Hospital												
No university hospital												

	Center											Total
						HO						Pct
	CHA	KAU	KNK	ZWI	REI	W	KBO	TUB	REU	RUD	N	N
**Team organization**												
hybrid												
autonomous												

Table 6: Descriptive analysis of the duration of the index treatment

Duration of the index treatment	N	N miss	Mean	Std Dev	Min	Q1	Median	Q3	Max
IG									
IG+									
CG									
CG+									

Table 7: Occupational status before and after the index treatment in both treatment groups

Professional status	IEHT		Fully stationary	
	Baseline	12-month FU	Baseline	12-month FU
Self-employed / working in the open labor market - full-time				
Self-employed / working in the open labor market - part-time				
Marginal employment				
Unemployed / job seeker				

Professional status	IEHT		Fully stationary	
	Baseline	12-month FU	Baseline	12-month FU
Disability or occupational disability				
Old-age pension / early retirement				
Training / retraining				
Housewife / Househusband				
Voluntarily (unpaid) employed				
Protected work				
Miscellaneous				

Table 8: Example of a table for the tertiary evaluation of a continuous variable

	IEHT				Fully stationary			
	N	Mea n	Std Dev	Media n	N	Mea n	Std Dev	Media n
Full inpatient readmission								
Yes								
no								

Table 9: Example of a table for the tertiary evaluation of a categorical variable, where n=number of patients, k=number of patients who were readmitted, %=readmission rate

Employment situation	IEHT				Fully stationary			
	n	k	%	n miss	n	k	%	n miss
In the employment relationship								
Not in an employment relationship								

Table 10: List of SUEs for one of the treatment groups

Patient ID	Description SUE (free text)	Conse-quences of the SUE	Date	Study period	Refe-rence to the study	Reason	Conse-quence regarding the study	Further information

Table 11: Number of discontinuations of the index treatment in both treatment groups broken down according to the various reasons

Number of index treatment discontinuations due to...	IEHT	Fully stationary
Serious external events		
Patient's request against medical advice		
Disciplinary reasons or non-compliance		
Death of the patient		
Reason cannot be determined further		

Number of index treatment discontinuations due to...	IEHT	Fully stationary
Other reasons		
Total		

Table 12: Mean satisfaction and category scores of employees per center. The center names should be pseudonymized.

	Center					Total
	1	2	…	9	10	
ZUF-1			…			
Job satisfaction according to COPSOQ			…			
COPSOQ: Quantitative requirements			…			
COPSOQ: Emotional requirements			…			
COPSOQ: Hide emotions			…			
COPSOQ: Work-privacy conflicts			…			
…			…			
COPSOQ: Inability to switch off			…			
ZFP: General work situation			…			
ZFP: Situation in the team			…			
ZFP: Information flow/communication			…			
ZFP: Supervisor			…			
ZFP: Dealing with conflicts			…			
ZfP: Organization/processes			…			
Workload according to ZFP FB			…			

	Center					Total
	1	2	...	9	10	
ZFP: Summarized assessment			...			
ZFP: Process evaluation			...			

Table 13: Mean values of the satisfaction and category scores of the employees per occupational group and number of values

	Occupational group								Total	
	Doctors		Psychologists		...		other			
	N	Mean	N	Mean	N	Mean	N	Mean	N	Mean
ZUF-1					...	...				
Job satisfaction according to COPSOQ					...	...				
COPSOQ: Quantitative requirements					...	...				
...					...	...				
COPSOQ: Inability to switch off					...	...				

	Occupational group								Total	
	Doctors		Psychologists		...		other			
	N	Mean	N	Mean	N	Mean	N	Mean	N	Mean
ZFP: General work situation					...	...				
...					...	...				
ZFP: Process evaluation					...	...				

Table 14: Mean values (with standard deviation and number of values) of the items from which the ZUF-1 is formed

	N	N miss	Mean	Std Dev	Min	Q1	Median	Q3	Max
ZUF-1									
Z6_6									
Z7_17									
Z8_24									
Z9_29									
Z10_33									
Z11_43									
Z12_44									
Z13_52									

Table 15: Response frequencies for question A2 of the employee questionnaire in the individual centers

A2: Gender	Center										
	1	2	3	4	5	6	7	8	9	10	Total
Missing											
Male											
Female											
diverse											

Table 16: Descriptive evaluation of question Z4 : IEHT members of the employee questionnaire

Z4: Proportion of IEHT work (%)	N	N miss	Mean	Std Dev	Min	Q1	Median	Q3	Max
Center									
1	18								
2	18								
3	18								
4	18								
5	18								
6	18								
7	18								
8	18								
9	18								
10	18								
Total	180								

13.2. Employee survey scales

In the Table 17 shows the scales of the employee survey with the associated questions, number of items, instrument and interpretation. CPAT stands for the German translation of the Collaborative Practice Assessment Tool (Holzke), MA-ZfP for the employee survey from Centrum for Psychiatry Südwürttemberg of the Metrik research group (2012), IPT for the questionnaire on the quality of our teamwork from the Institute for Psychology Transfer

Bamberg and TeamPuls for test procedures for team diagnosis developed by TU Dresden and Management Innovation Dresden in the TeamPuls specialist group (2011).

Table 17: Scales of the employee survey

Scale	Questions	Quantity	Instrument	Interpretation
**Requirements**			**Score: mean value**	
Quantitative requirements	B1: 1-5	5	COPSOQ, inverted	**100 = negative**
Emotional requirements	B1: 6-7	2	COPSOQ, inverted	**100 = negative**
Hide emotions	B1: 8-9	2	COPSOQ, inverted	**100 = negative**
Work-privacy conflicts	B2: 1-4	4	COPSOQ, inverted	**100 = negative**
Dissolution of boundaries	B2: 5-6	2	COPSOQ, inverted	**100 = negative**
**Influence and development opportunities**			**Score: mean value**	
Influence on the work	B3: 1-3	3	COPSOQ, inverted	**100 = positive**
Scope for breaks and vacation	B3: 4-5	2	COPSOQ, inverted	**100 = positive**
Development opportunities	B4: 1, B5: 1-2	3	COPSOQ, inverted	**100 = positive**
Importance of the work	B5: 3-4	2	COPSOQ, inverted	**100 = positive**
Attachment to the workplace	B5: 5-6	2	COPSOQ, inverted	**100 = positive**
**Social relationships and leadership**			**Score: mean value**	
Predictability of the work	B6: 1-2	2	COPSOQ, inverted	**100 = positive**
Role clarity	B6: 3-5	3	COPSOQ, inverted	**100 = positive**
Role conflicts	B6: 6-8	3	COPSOQ, inverted	**100 = negative**
Leadership quality	B7: 1-4	4	COPSOQ, inverted	**100 = positive**
Support at work	B8: 1-4	4	COPSOQ, inverted	**100 = positive**
Feedback / Feedback	B8: 5-6	2	COPSOQ, inverted	**100 = positive**
Amount of social contacts	B8: 7	1	COPSOQ, inverted	**100 = positive**
Sense of community	B8: 8-9	2	COPSOQ, inverted	**100 = positive**
Unfair treatment	B8: 10	1	COPSOQ, inverted	**100 = negative**
Trust and justice	B8a: 1-4	4	COPSOQ, inverted	**100 = positive**
Appreciation	B8a: 5	1	COPSOQ, inverted	**100 = positive**
**Other factors**			**Score: mean value**	
Working environment / phys. Requirements	B8b: 1-6	6	COPSOQ, inverted	**100 = negative**
Job insecurity	B9: 1-3	3	COPSOQ, inverted	**100 = negative**
Uncertainty of working conditions	B9: 4-6	3	COPSOQ, inverted	**100 = negative**
**Effects**			**Score: mean value**	

Scale	Questions	Quantity	Instrument	Interpretation
Thinking of changing career / job	B10: 1-2	2	COPSOQ	**100 = negative**
Job satisfaction	B11: 1-7	7	COPSOQ, inverted	**100 = positive**
Commitment to work	B14: 1-3	3	COPSOQ, inverted	**100 = positive**
General state of health	B12	1	COPSOQ	**100 = positive**
Burnout symptoms	B13: 1-3	3	COPSOQ, inverted	**100 = negative**
Presenteeism	B13: 4	1	COPSOQ, inverted	**100 = negative**
Inability to switch off	B13: 5	1	COPSOQ, inverted	**100 = negative**
**General work situation**			**Score: mean value**	**100 = positive**
Work content	Z6: 1	1	MA- ZFP (3)	
Working time regulation	Z6:2	1	MA- ZFP (41)	
Resources	Z6: 3-5	3	MA- ZFP(45); new; new	
Overall satisfaction Work situation	Z6: 6	1	new	
**Situation in the multi-professional, cross-professional team**			**Score: mean value**	**100 = positive**
Team decisions	Z7: 7	1	CPAT (responsibility and autonomy 38)	
Mutual reliability	Z7:8	1	CPAT (General relations 15)	
Team communication	Z7: 9	1	New	
Team spirit	Z7: 10	1	MA- ZFP (8)	
Team collaboration	Z7: 11	1	MA- ZFP (47 page4)	
Trust and respect in the team	Z7: 12	1	MA- ZFP (E2 page7)	
Position in the team	Z7: 13	1	MA- ZFP (E4 page7)	
Distribution of tasks	Z7: 14-16	3	New;<br>new<br>MA- ZFP (further topics/ f) p.7)<br>negatively polarized	Yes, rescale
Satisfaction Cooperation	Z7: 17	1	new	
**Information flow / communication**			**Score: mean value**	**100 = positive**
Passing on information	Z8: 18	1	TeamPulse(13, S2)	

Scale	Questions	Quantity	Instrument	Interpretation
Meeting structure	Z8: 19-23	5	No.19 IPT(3)<br>new<br>No.21 TeamPuls(8, S1)<br>new<br>new	
Satisfaction Information flow	Z8: 24	1	new	
**Supervisor**				**100 = positive**
Instructions	Z9: 25	1	MA- ZFP (14, S2)	
Accessibility	Z9: 26	1	new	
Feedback	Z9: 27	1	new	
Transparency	Z9: 28	1	MA- ZFP (17, S2)	
Supervisor satisfaction	Z9: 29	1	new	
**Dealing with conflicts**			**Score: mean value**	**100 = positive**
Openness	Z10: 30	1	MA- ZFP (22, S3)	
Participation in conflict resolution	Z10: 31	1	MA- ZFP (23, S3)	
Ability to deal with conflict	Z10: 32	1	CPAT (Decision Making and Conflict Management 49) NR. 32 negatively polarized	Yes, rescale
Satisfaction Conflicts	Z10: 33	1	new	
**Organization / Processes**			**Score: mean value**	**100 = positive**
Processes	Z11: 34	1	MA- ZFP (29, S3)	
Regulation of responsibility	Z11: 35	1	MA- ZFP (30, S3)	
Regulation of cooperation with other areas	Z11: 36	1	MA- ZFP (31, S3)	
Familiarization	Z11: 37	1	MA- ZFP (a, S7)	
Substitution rules	Z11: 38	1	TeamPulse (11,S1)	
Coordination of the work	Z11: 39-40	2	New:<br>CPAT (responsibility and autonomy 33)	

Scale	Questions	Quantity	Instrument	Interpretation
Freedom of choice	Z11: 41-42	2	MA- ZFP (21,S3); new	
Organization satisfaction	Z11: 43	1	new	
**Workload**			**Score: mean value**	**100 = positive**
General workload	Z12: 44	1	new	
Mental workload	Z12: 45	1	new	
Physical workload	Z12: 46	1	MA- ZFP (d,S7)	
Experience of violence	Z12: 47	1	MA- ZFP (l,S7) negatively polarized	Yes, rescale
Experience of humiliation / discrimination	Z12: 48	1	MA- ZFP (m,S7) negatively polarized	Yes, rescale
**Summarized evaluation**			**Score: mean value**	**100 = positive**
Renewed choice of job	Z13: 49	1	MA- ZFP (4,S2)	
Increase in job satisfaction	Z13: 50	1	MA- ZFP (49,S4)	
Recommendation of the work [illegible]	Z13: 51	1	MA- ZFP (40,S4)	
Satisfaction with job	Z13: 52	1	new	
**Process evaluation**			**Score: mean value**	**100 = positive**
Information in advance	Z14: 1; 3; 16	3	No.1 new<br>No.3 new<br>No.3 new (negative polarity)<br>No.16 new	Yes, rescale (item 3)
Participation opportunities	Z14: 2;9-11	4	No.2 new<br>No.9-11 new<br>No.9 negative polarity	Yes, rescale (Item9)
Transparency of the project	Z14: 4-5	2	No.4 new<br>No.5 new	
Voluntary choice of workplace	Z14: 6	1	new	
Evaluation of the processes	Z14: 7; 14-15	3	new	
Satisfaction with the project	Z14: 8; 13	2	new	
Satisfaction Implementation	Z14:12	1	new	

13.3. List of clinics

Table 18List of centers

Clinic abbreviation	Clinic name	Project management
**CHA**	Charité – Berlin University Hospital	Project management North-East
**KAU**	Berlin-Vivantes Hospital am Urban	Project management North-East
**KNK**	Berlin-Vivantes Hospital Neukölln	Project management North-East
**RUD**	Immanuel Clinic Rüdersdorf	Project management North-East
**KBO**	Isar-Amper Hospital Munich	Project Management South-West
**REU**	Reutlingen Clinic	Project Management South-West
**HOW**	Center for Psychiatry Südwürttemberg-Clinic Weissenau	Project Management South-West
**ZWI**	Centrum for Psychiatry Südwürttemberg-Clinic Zwiefalten	Project Management South-West
**REI**	Center for Psychiatry Südwürttemberg-Clinic Südwürttemberg-Clinic Reichenau	Project Management South-West
**TUB**	University Hospital Tübingen	Project Management South-West

13.4. Table 2 from Ludwig et al.

Table 19: Table 2 from Ludwig et al.

Table 2 Parameter estimates for main effects models

Independent variables of the model[a,b]	Model 1: Tobit (cTTO model)		Model 2: Conditional Logit (DCE model)			Model 3a: Hybrid censoring at -1 (cTTO + DCE model)[c]		Model 3b (Value Set): Hybrid censoring at -1 and correcting for heteroskedasticity (cTTO + DCE model)[d,e]	
	β (SE)	*p*	*β* (SE)	*p*	rescaled *β*	*β* (SE)	*p*	*β* (SE)	*p*
**MO2: slight problems**	0.028 (0.015)	0.062	0.135 (0.057)	0.019	0.023	0.028 (0.008)	0.000	0.026 (0.006)	0.000
**MO3: moderate problems**	**0.015** (0.017)	0.379	0.370 (0.069)	0.000	0.063	0.051 (0.009)	0.000	0.042 (0.009)	0.000
**MO4: severe problems**	0.130 (0.018)	0.000	0.834 (0.069)	0.000	0.141	0.139 (0.009)	0.000	0.139 (0.009)	0.000
**MO5: unable**	0.207 (0.017)	0.000	1.349 (0.077)	0.000	0.228	0.216 (0.009)	0.000	0.224 (0.009)	0.000
**SC2: slight problems**	0.035 (0.014)	0.013	0.408 (0.063)	0.000	0.069	0.058 (0.008)	0.000	0.050 (0.006)	0.000
**SC3: moderate problems**	0.050 (0.018)	0.006	**0.393** (0.070)	0.000	**0.067**	0.062 (0.009)	0.000	0.056 (0.008)	0.000
**SC4: severe problems**	0.174 (0.017)	0.000	1.034 (0.072)	0.000	0.175	0.174 (0.009)	0.000	0.169 (0.009)	0.000
**SC5: unable**	0.244 (0.016)	0.000	1.520 (0.071)	0.000	0.257	0.248 (0.008)	0.000	0.260 (0.008)	0.000
**UA2: slight problems**	0.034 (0.015)	0.024	0.119 (0.059)	0.044	0.020	0.025 (0.008)	0.001	0.036 (0.006)	0.000
**UA3: moderate problems**	0.069 (0.016)	0.000	0.232 (0.066)	0.000	0.039	0.049 (0.009)	0.000	0.049 (0.008)	0.000
**UA4: severe problems**	0.121 (0.017)	0.000	0.669 (0.070)	0.000	0.113	0.117 (0.009)	0.000	0.129 (0.008)	0.000
**UA5: unable**	0.203 (0.016)	0.000	1.130 (0.073)	0.000	0.191	0.191 (0.009)	0.000	0.209 (0.008)	0.000
**PD2: slight problems**	0.061 (0.013)	0.000	0.421 (0.063)	0.000	0.071	0.066 (0.008)	0.000	0.057 (0.006)	0.000
**PD3: moderate problems**	0.098 (0.018)	0.000	0.739 (0.070)	0.000	0.125	0.119 (0.009)	0.000	0.109 (0.009)	0.000
**PD4: severe problems**	0.423 (0.016)	0.000	2.264 (0.079)	0.000	0.383	0.397 (0.010)	0.000	0.404 (0.010)	0.000
**PD5: extreme problems**	0.558 (0.017)	0.000	3.516 (0.098)	0.000	0.595	0.577 (0.010)	0.000	0.612 (0.011)	0.000
**AD2: slight problems**	0.036 (0.014)	0.012	0.183 (0.067)	0.007	0.031	0.033 (0.008)	0.000	0.030 (0.005)	0.000
**AD3: moderate problems**	0.106 (0.017)	0.000	0.439 (0.070)	0.000	0.074	0.085 (0.009)	0.000	0.082 (0.008)	0.000
**AD4: severe problems**	0.250 (0.016)	0.000	1.378 (0.078)	0.000	0.233	0.236 (0.009)	0.000	0.244 (0.008)	0.000
**AD5: extreme problems**	0.345 (0.016)	0.000	1.985 (0.080)	0.000	0.336	0.334 (0.009)	0.000	0.356 (0.009)	0.000
**Observations included in model**	10,867		8,106			18,973		18,973	
Continuous uncensored (cTTO)	9,818		-			9,818		9,818	
Continuous left-censored (cTTO)	1,049		-			1,049		1,049	
Dichotomous observations (DCE)	-		8,106			8,106		8,106	

13.5. Seeds for imputations

Table 20: Seeds for imputations

Chapter	Target variable	Brief description Analysis	Seeds
11.2.1.1	Full inpatient readmission rate	Primary analysis	211011, 211012
11.2.1.2	Full inpatient readmission rate	Sensitivity analysis Model 1	212011, 212012
11.2.1.2	Full inpatient readmission rate	Sensitivity analysis model 2	212021, 212022
11.2.1.2	Full inpatient readmission rate	Sensitivity analysis model 3	212031, 212032
11.2.1.2	Full inpatient readmission rate	Sensitivity analysis model 4	212041, 212042
11.2.1.2	Full inpatient readmission rate	Sensitivity analysis collective of survivors	212051, 212052
11.2.2.1	Combined readmission rate	Secondary analysis	221011, 221012
11.2.2.2	Extended readmission rate	Secondary analysis	222011, 222012
11.2.2.7	Health-related quality of life	Secondary analysis	227011, 227012, 227013, 227014
11.2.2.8	Psychosocial functioning level (HoNOS)	Secondary analysis	228011, 228012, 228013, 228014
11.2.2.8	Psychosocial functioning level (PSP)	Secondary analysis	228021, 228022, 228023, 228024
11.2.2.9	Professional integration	Secondary analysis	229011, 229012, 229013, 229014
11.2.2.10	Recovery orientation	Secondary analysis	2210011, 2210012, 2210013, 2210014
11.2.2.11	Perceived involvement in decisions	Secondary analysis	2211011
11.2.2.12	Treatment satisfaction	Secondary analysis	2212011
11.2.2.13	Health-related quality of life	Sensitivity analysis +- 14 days	2213011, 2213012, 2213013, 2213014
11.2.2.13	Psychosocial functioning level (HoNOS)	Sensitivity analysis +- 14 days	2213021, 2213022, 2213023, 2213024
11.2.2.13	Psychosocial functioning level (PSP)	Sensitivity analysis +- 14 days	2213031, 2213032, 2213033, 2213034
11.2.2.13	Change in professional integration	Sensitivity analysis +- 14 days	2213041, 2213042, 2213043, 2213044
11.2.2.13	Recovery orientation	Sensitivity analysis +- 14 days	2213051, 2213052, 2213053, 2213054
11.2.2.13	Perceived involvement in decisions	Sensitivity analysis + 7 days	2213061
11.2.2.13	Treatment satisfaction	Sensitivity analysis + 7 days	2213071
11.2.2.13	Combined readmission rate	Sensitivity analysis logit model	2213081, 2213082
11.2.2.13	Extended readmission rate	Sensitivity analysis logit model	2213091, 2213092
11.2.2.13	Change in professional integration	Sensitivity analysis logit model	2213101, 2213102, 2213103, 2213104
11.2.2.13	Treatment satisfaction	Sensitivity analysis linear model	2213111
11.2.2.13	Psychosocial functioning level (HoNOS)	Sensitivity analysis linear model	2213121, 2213122, 2213123, 2213124
11.2.2.13	Recovery orientation	Sensitivity analysis linear model	2213131, 2213132, 2213133, 2213134
11.2.2.13	Health-related quality of life	Sensitivity analysis logit model	2213141, 2213142, 2213143, 2213144

Chapter	Target variable	Brief description Analysis	Seeds
11.2.2.13	Psychosocial functioning level (PSP)	Sensitivity analysis logit model	2213151, 2213152, 2213153, 2213154
11.2.2.13	Perceived involvement in decisions	Sensitivity analysis logit model	2213161
11.2.3	Full inpatient readmission rate	Tertiary analysis large model	23011, 23012
11.2.3	Full inpatient readmission rate	Tertiary analysis Age	23021, 23022
11.2.3	Full inpatient readmission rate	Tertiary analysis Previous stays	23031, 23032
11.2.3	Full inpatient readmission rate	Tertiary analysis Gender	23041, 23042
11.2.3	Full inpatient readmission rate	Tertiary analysis Diagnostic group	23051, 23052
11.2.3	Full inpatient readmission rate	Tertiary analysis Place of residence	23061, 23062
11.2.3	Full inpatient readmission rate	Tertiary analysis Employment situation	23071, 23072
11.2.3	Full inpatient readmission rate	Tertiary analysis Relocation	23081, 23082
11.2.3	Full inpatient readmission rate	Tertiary analysis Age at first stay	23091, 23092
11.2.3	Full inpatient readmission rate	Tertiary analysis of inpatient stays	23101, 23102
11.2.3	Full inpatient readmission rate	Tertiary analysis Duration Pretreatment	23111, 23112
11.3.1.4	Full inpatient readmission rate	Influence of hybrid/autonomous teams	314011, 314011
11.3.1.4	Extended readmission rate	Influence of hybrid/autonomous teams (sensitivity analysis)	314021, 314021
11.3.1.6	Full inpatient readmission rate	Influence of treatment stability	316011, 316012
11.3.1.6	Full inpatient readmission rate	Influence on stability Details	316021, 316022
11.3.1.6	Full inpatient readmission rate	Influence of index treatment termination	316031, 316032
11.3.1.6	Full inpatient readmission rate	Influence of individual instability variables	316041, 316042
11.3.1.6	Extended readmission rate	Influence of treatment stability (sensitivity analysis)	316051, 316052
11.3.1.6	Extended readmission rate	Influence of stability Details (sensitivity analysis)	316061, 316062
11.3.1.6	Extended readmission rate	Influence of discontinuation of index treatment (sensitivity analysis)	316071, 316072
11.3.1.6	Extended readmission rate	Influence of individual instability variables (sensitivity analysis)	316081, 316082
