# Supplementary material for: Utilization of Psychiatric Hospital Services Following Intensive Home Treatment: A Nonrandomized Clinical Trial
Source: JAMA Netw Open. 2024 Nov 15;7(11):e2445042. doi: 10.1001/jamanetworkopen.2024.45042 (PMC11568461; doi:10.1001/jamanetworkopen.2024.45042)
Supplement: Supplement 4. — Data Sharing Statement [file jamanetwopen-e2445042-s004.pdf]

Data Sharing Statement

Bechdolf. Utilization of Psychiatric Hospital Services Following Intensive Home Treatment. *JAMA Netw Open*. Published November 14, 2024. doi:10.1001/jamanetworkopen.2024.45042

Data
Additional Information: ClinicalTrials.gov identifier: NCT04745507
Data available: Yes
Data types: Deidentified participant data, Data dictionary
How to access data: Request for data to corresponding author, Prof. Andreas Bechdolf, Andreas.bechdolf@charite.de
When available: With publication

Supporting Documents
Document types: None

Additional Information
Who can access the data: Researchers whose proposed use of the data has been approved.
Types of analyses: For specified purposes.
Mechanisms of data availability: With investigator support, after approval of a proposal.
